# Crying in the first 12 months of life: A systematic review and meta‐analysis of cross‐country parent‐reported data and modeling of the “cry curve”

**DOI:** 10.1111/cdev.13760

**Published:** 2022-04-19

**Authors:** Arnault‐Quentin Vermillet, Katrine Tølbøll, Samouil Litsis Mizan, Joshua C. Skewes, Christine E. Parsons

**Affiliations:** ^1^ Interacting Minds Center Aarhus University Aarhus Denmark; ^2^ Department of Clinical Medicine Aarhus University Aarhus Denmark; ^3^ Department for Linguistics, Cognitive Science, and Semiotics Aarhus University Aarhus Denmark

## Abstract

Crying is an ubiquitous communicative signal in infancy. This meta‐analysis synthesizes data on parent‐reported infant cry durations from 17 countries and 57 studies until infant age 12 months (*N* = 7580, 54% female from *k* = 44; majority White samples, where reported, *k* = 18), from studies before the end Sept. 2020. Most studies were conducted in the United States, the United Kingdom, and Canada (*k* = 32), and at the traditional cry “peak” (age 5–6 weeks), where the pooled estimate for cry and fuss duration was 126 mins (*SD* = 61), with high heterogeneity. Formal modeling of the meta‐analytic data suggests that the duration of crying remains substantial in the first year of life, after an initial decline.

**Abbreviations**
CPQ: Crying Pattern Questionnaire
DIC: deviance information criterion
RCT: randomized controlled trial
SMD: standardized mean difference

## INTRODUCTION

In 1962, Brazelton reported a seminal analysis of infants’ cry durations over the first 12 weeks of life, recorded by 80 mothers attending a private pediatric practice in Massachusetts, USA (Brazelton, 1962). In what has now come to be known as the “infant cry curve,” mothers reported gradual increases in their infants’ cry duration until 6 weeks, where crying and fussing had a “peak” average duration of 2.75 h per day. The infant cry curve has since become widely recognized by parents and clinicians as describing a predictable pattern, peaking at 6 weeks, then declining steadily until 12 weeks. Numerous studies in Western countries have replicated the early pattern of a peak and decline (Hunziker & Barr, 1986; Rebelsky & Black, 1972), and the cry curve has been described as the “most robust feature of infant crying” (Barr et al., 1987). Depictions of the infant “cry curve” feature centrally in psychoeducational materials for parents on infant crying (Barr, n.d.) and in clinician‐facing training materials (e.g., Royal Children's Hospital, 2019).

While the infant cry curve provides an intuitive depiction, it was originally derived from data until 12 weeks postpartum, and points to a steep decline in cry duration and minimal cry durations in later months. The accuracy of the cry curve depiction is important, because parents’ and indeed clinicians’ understanding of normative cry patterns will shape perceptions of normal or excessive crying. When crying is perceived as excessive in duration or intensity, or difficult to console, it is associated with negative consequences for parent and infant alike (Barr, 1990a). “Excessive” crying is associated with early termination of breastfeeding (Howard et al., 2006), parental distress, and symptoms of depression (Kurth et al., 2011; Murray & Cooper, 2001).

While Brazelton’s (1962) analysis is arguably best‐known for its description of “average” infant cry patterns, he also reported non‐trivial variability in crying between infants. The variability in infant cry patterns, and the reasons for such variability, have been the focus of considerable research. Evidence for the environmental determinants of infant crying behavior has emerged from observational studies of parenting behaviors, from trials intervening to change parenting behaviors, and from cross‐country descriptions. For example, higher maternal responsiveness to crying has been associated with greater subsequent cry duration (van IJzendoorn & Hubbard, 2000). Intervening to increase the amount of maternal carrying of the infant has also been associated with alterations to the “cry curve” (Hunziker & Barr, 1986). Anthropological studies of non‐Western and less industrialized societies also suggest radically different approaches to infant crying and reported infant cry durations. The Zhun/twa mothers from southern Africa, for example, have been described as “making every effort to anticipate hunger,” and pre‐emptively feeding, rather than engaging in cry‐signaled feeding typical in Western countries (Konner, 1972).

Given the numerous environmental factors that might affect infant cry duration, a 2017 review aimed to synthesize the available data from parent‐completed diary‐based studies carried out until infant age 12 weeks. Including data from nine countries until 2015, this analysis suggested that “the duration of infant fussing or crying drops significantly after 8–9 weeks of age” (Wolke et al., 2017), and the authors noted “no statistical evidence for a universal crying peak at 6 weeks.” The authors further reported that feeding type was related to cry durations, where, for example, samples that included breastfed infants had significantly higher fuss/cry durations at 3–4 weeks of age than the overall average. Finally, an association was also noted between the country of the infants’ families, with the most consistent finding being that Danish infants cried less across multiple time points than infants in other countries. These cross‐country differences, particularly related to Denmark, were widely cited in the media (e.g., O’Connell, 2017; Ramaswamy, 2017).

While the most dramatic changes in infant cry patterns occur in the first 3–4 months, crying and fussing remain a substantial component of infants’ communicative repertoire throughout the first year of life. Beyond the 12‐week period examined by Wolke et al. (2017), persistent crying can remain a challenge for parents, and after 6 months of age, may be associated with risk for additional infant regulatory difficulties (von Kries et al., 2006). In this preregistered review, we, therefore, synthesized data until 12 months, using both diary‐based and validated parent‐reported questionnaires to provide a comprehensive, updated mapping of available data.

In an exploratory analysis of the data added to the preregistered review, we developed a formal mathematical model of the continuous change in crying over the first 12 months of life, based on the classic “cry curve.” We use the data from our 12‐month meta‐analytic synthesis to compare our model's performance to that of three alternative statistical models, one based on a linear decay of cry duration, one on an exponential decay, and one assuming a period of high constant crying followed by a period of exponential decay. In addition to providing a formalization of the continuous change in cry duration over time, we can explicitly infer the age at which peak cry duration occurs. Our modeling approach also allows us to make inferences about other characteristics of the crying curve, such as the magnitude of the peak (e.g., maximum time spent crying per day), and the minimum cry duration, along with estimates of uncertainty.

## METHOD

The study was conducted following the PRISMA recommendations for reporting systematic reviews and meta‐analyses (Liberati et al., 2009) and preregistered with PROSPERO (Booth et al., 2012), registration number (CRD42020210934). We searched the databases Scopus, PsycINFO, and PubMed using the keywords (and MeSH terms): (i) “infant" AND ("crying” OR "fussing") AND (diary OR questionnaire OR duration). Search dates were from “unrestricted” until Sept 2020. We excluded review articles (narrative and systematic reviews, meta‐analyses, editorials, and non‐peer‐reviewed sources; see Supporting Information for the specification for each database used). We restricted inclusion to English language articles only.

We included all study designs that reported on infant cry duration data until the infant age of 12 months. This included randomized controlled trial, cross‐sectional studies (e.g., birth cohort studies), longitudinal designs (e.g., prospective cohort studies). We excluded studies that included only experimental interventions, and no non‐experimental control group (i.e., treatment as usual, or wait list). Studies were included if they reported on a specific time point (e.g., 6 weeks) with a maximum range of 2 weeks around that time point. Where authors did not report on the age range but did report the mean and *SD* of age, we accepted a maximum *SD* of 3.5 days, such that 95% of infant ages within each study fell within a 2‐week range. We included studies that measured both infants with colic and infants categorized as non‐colic or healthy and synthesized data with the non‐colic groups. Where available, we report on data included in the control arm of an intervention only. If studies reported that there were no differences between the control arm and the experimental arm, and these data were not reported separately, we included the combined datasets (see Fujiwara et al., 2012), where combined results from the intervention and control arms only were reported (additionally reported in Barr, Barr, et al., 2009; Barr et al., 2009a; Radesky et al., 2013).

### Missing data

Where data were not reported in the text, we used WebPlotDigitizer to extract mean and *SD* values (Rohatgi, 2017). We contacted 14 authors to obtain missing data or clarify the data presented in the manuscript, and received data from four of these authors (Anzman‐Frasca et al., 2013; Bilgin & Wolke, 2020a, 2020b; Jordan et al., 2020; Wynter et al., 2014) and the requested clarification from one author (Aloisio et al., 2018). One study was excluded because the age range of the infants was wider than the specified inclusion criteria (Wynter et al., 2014). For the six studies with missing *SD* data, we used the Bracken (1992) imputation method, taking the coefficient of variation from all complete cases, which is the *SD* divided by its mean. Study data for Blum et al. (2002) were extracted from Wolke et al. (2017). Duplicates were removed using EndNoteX8 (2019) and Rayyan software functionality (Ouzzani et al., 2016). Rayyan software was used for “title and abstract” and “full‐text” screening, with two independent reviewers (CP, SLM). Data extraction was performed independently by CP and SLM using customized spreadsheets.

We extracted data for eight criteria, following the quality‐assessment procedure described by Wolke et al. (1) subject selection (whole vs. defined population), (2) recruitment rate (≥50% vs. <50%), (3) participation rate (≥75% vs. <75%), (4) sample size (≥ 101 vs. <101), (5) whether 4‐sample characteristics were reported: socioeconomic status, parity, infant sex, parent age (3 of 4 reported vs. <3 reported), (6) feeding (reported vs. not), (7) resolution time for diary (5 vs. 15 min), (8) number of days requested for diary (≥4 vs. <4 days). Each sample received a score of 0 or 1 for each of the criteria. A score of 0 was given where the information for the criterion was not reported. Individual scores were summed to give a total quality score (0 to 8). For feeding type, we created a 5‐group categorization based on authors’ reports of the feeding type: (1 = majority >70% breastfeeding; 2 = majority bottle‐feeding; 3 = Breastfeeding only; 4 = Bottle only; 5 = Mixed, NA = no data).

### Publication bias

Publication bias was assessed using two methods suggested for observational studies: the Begg and Mazumdar rank correlation test (Begg & Mazumdar, 1994) and the Duval and Tweedie “trim and fill” method (Duval & Tweedie, 2000). These methods rely on the assumption that small studies with non‐significant results are less likely to be published than studies with significant results, which may lead to overestimation of the true mean effect size. Therefore, they test for the absence of small non‐significant studies in the data, and estimates can be adjusted based on the results. We used funnel plots to visualize the distribution of effect sizes (Peters et al., 2008).

### Statistical analysis

The R packages (version 4.0.5) metagear, meta, metafor, car, tidyverse, patchwork, and tmap were used for data synthesis, visualizations, and analyses (Balduzzi et al., 2019; Lajeunesse, 2016; R Core Team, 2021; Tennekes, 2018; Viechtbauer, 2010; Wickham, 2019). To synthesize cry duration data, we calculated a mean (weighted by sample size) and the pooled weighted *SD* for 2‐week intervals until infant age 12 weeks (6 intervals) and from 13 weeks, for 4‐week intervals. This categorization until 12 weeks replicated the time intervals reported by Wolke et al. (2017). Given that fewer studies reported on data from infant age 13 weeks onwards relative to before 13 weeks, we created 4‐week categorizations to maximize number of studies available to pool. To test differences in cry durations across these age intervals, we replicated the analysis reported by Wolke et al. (2017), but using fixed‐effects regression to test the overall effect of age intervals. We synthesize three cry outcome variables in presenting our overall results: “crying and fussing,” “crying only” and “total distress”—including crying, fussing, and inconsolable crying. All additional analyses use “crying and fussing” as the outcome variable, given its status as the most frequently reported outcome.

Effect sizes are also reported as the standardized mean difference with 95% CIs for each study. The mean difference compares the individual study's mean with the overall weighted mean across studies, for each age interval. Hedge's *g*, a variation of Cohen's *d* that corrects for biases due to small sample sizes (Hedges & Olkin, 1985), was selected as the appropriate effect size, given the sample size of a number of the included studies (23% of samples across studies included less than 35 infants). Effect sizes were analyzed using random effects models, in which the error term is composed of variation originating from both within‐study variability and between‐study differences. All materials related to the analyses are available on the Open Science Framework, with the following link: https://osf.io/dzhvm/.

Following the prior synthesis of parent‐reported cry data (Wolke et al., 2017), and studies demonstrating cross‐country differences in parenting practices that might be associated with infant crying (e.g., St. James‐Roberts et al., 2006), we expected to confirm the presence of cross‐country differences in cry duration. We furthermore expected to confirm an overall, but nonlinear, decline in cry duration with infant age, based on prior descriptions of infant cry developmental patterns (Barr, 1990b). We did not have a specific hypothesis regarding the direction of effects for two of the additional moderators tested, infant feeding type or study quality, because of the limited evidence base available for these.

### Heterogeneity

Heterogeneity was explored using *Q* and *I2* statistics (Sterne et al., 2008). Due to the low statistical power of heterogeneity tests, we used a *p*‐value of ≤.10 to determine significant heterogeneity (Poole & Greenland, 1999). The *I2* statistic is an estimate of the amount of variance in a pooled effects size accounted for by heterogeneity in the sample of studies, and is not sensitive to the number of studies (Higgins et al., 2003). An *I2* value of 0% indicates no observed heterogeneity, while values of 25%, 50%, and 75% are considered low, moderate, and high, respectively. Categorical moderator tests were applied to test for between groups heterogeneity (*Q*_b_). A significant value for *Q*_b_ indicates that the effect sizes are significantly different across distinct categories of the moderator variable. Meta‐regression analyses were performed to test quality of assessment as a continuous moderator, and for feeding type (five categories).

### Modeling of the cry curve

In an extension of the preregistered review, we modeled continuous change in cry duration during the first year of life, as presented in the classic “cry curve,” with a peak and then decline cry duration pattern. Based on the results of the analysis presented in Wolke et al. (2017), we also developed a change‐point detection model. The change‐point model represents the data in two distinct periods, starting with a period of constant high cry duration followed by an exponential decay. We then compared this classic cry curve models and our change‐point model to alternatives, using data from the meta‐analysis. This modeling approach provides a more informative way to synthesize available data than presenting cross‐sectional data points alone. Although the age of peak infant crying time is conceptualized in continuous time (or on a scale of days), most studies report either cross‐sectional data (see Table 1), or longitudinal data collected at discrete intervals (e.g., at 5–6 weeks and 11–12 weeks), a binning which entails information loss. To compound this problem, different longitudinal studies use different data collection intervals (e.g., 8–9 weeks, 9–10 weeks), such that cross‐study comparison requires binning of measurements into still larger intervals. A formalization of the continuous change in cry duration over developmental age can be more flexibly fitted to data within and across studies.

**TABLE 1:** Characteristics of the included studies

Study	Year	Country	Measure	Res.	Diary days (e.g., 3 days)	When was the data collected?	Recruit.	Part.	Char	Population	Feeding type: majority (>70)	SS	% Female	Ethnicity
Atella et al. (2003)	2003	USA	24‐h diary	5	3	NR	NR	77.37	3	Defined	Majority breastfeeding	106	48	Mothers: 88% European American, 9% African American, 3% Asian/Asian‐American
Barr et al. (1989)	1989	Canada	24‐h diary	5	7	1981–1982	84	69; 22	3	Whole	Separate groups	374	46	
St. James‐Roberts et al. (2001)	2001	UK	24‐h diary	5	3	NR	35	94	2	Whole	Mixed	191	—	Mothers: White: 92%
St. James‐Roberts et al. (1994)^a^	1994	India	CPQ	NA	NA	NR	NR	NR	2	Whole	All breastfeeding, then majority	100	49	
St. James‐Roberts et al. (2006)	2006	Denmark, UK	24‐h diary	5	4	NR	DK: 44; UK: 26	UK: 63.8; 46.5; 43.1; DK: 80.4 73.5; 72.4	4	Whole	Mixed for London, Denmark: All breastfeeding, then majority	70	LON: 52; DEN: 43	Denmark: 92% mothers, 89% fathers: White European/Danish national origins; London community group: White 75% mothers, 71% fathers
Aloisio et al. (2018)	2018	Italy	24‐h diary	NR	10	2013–2016	58.96	98.10	2	Whole	Majority breastfeeding	155	48	
Alvarez (2004)	2004	Denmark	24‐h diary	5	3	1997–1999	54.50	78.67	4	Whole	Majority breastfeeding	118	57	
DeLeon and Karraker (2007)	2007	USA	24‐h diary	15	7	NR	NR	NR	3	Whole	Mixed	41	37	Non‐Hispanic White (95%)
Anzman‐Frasca et al. (2013)	2013	USA	24‐h diary	15	4	NR	NR	66.88	4	Defined	Majority breastfeeding, Mixed at 16 weeks	49	59	86.4% White
Baildam et al. (1995)	1995	UK	24‐h diary	5	1	1978–1980	99.5	78.5	0	Whole	Majority bottle‐fed	157	48	
Bilgin and Wolke (2020a)	2020	UK	CPQ	NA	NA	NR	NR	NR	4	Whole	Majority bottle‐feeding	105	44	
Blum et al. (2002)	2002	USA	24‐h diary	5	4	NR	NR	53; 52; 51;	3	Whole	NR	60	50	Parents: White: 85%
Bolten et al. (2012)	2012	Switzerland	24‐h diary	15	3	NR	NR	73.62	4	Whole	NR	120	45	
Bonichini et al. (2008)	2008	Italy	24‐h diary	5	3	NR	NR	77.78	4	Whole	NR	70	53	
Clifford et al. (2002a)	2002a	Canada	24‐h diary	5	7	1999	84	69.85	2	Whole	Mixed	431	—	
Clifford et al. (2002b)	2002b	Canada	24‐h diary	5	7	1999	84	58.68	2	Whole	NR	320	—	
Darlington and Wright (2006)	2006	UK	24‐h diary	15	2	NR	NR	58.19;	2	Whole	Mixed	75	57	
de Weerth and Buitelaar (2007)	2007	NL	24‐h diary	NR	4	NR	NR	87.93	4	First born	Mixed	102	50	Mothers: 100% White
Fujiwara et al. (2012)	2011	USA, Canada	24‐h diary	5	4	2004–2006	54.11	67.82	3	Whole	NR	1857	‐	
Geeraerts et al. (2020)	2020	USA	24‐h diary	5	3	2009–2018	NR	80	3	Whole	NR	132	46	93.29%: White 2.01%: as Black/African American, 1.34%: Hispanic or Latino; 1.34% Asian; 2%: Native Hawaiian/Pacific Islander; American Indian/Alaskan Native, ethnicity not provided
Harrison (2004)	2004	UK	24‐h diary	5	3	2003	NR	77.78	1	Whole	NR	56	54	
Hechler et al. (2018)	2018	NL	24‐h diary	5	3	NR	NR	67.53	4	Defined	Majority breastfeeding	68	49	
Hiscock et al. (2014)	2014	Australia	24‐h diary	10	3	2010–2011	55.00	65.9; 54.7;	4	Whole	Majority breastfeeding	259	49	
Hunziker and Barr (1986)	1986	Canada	24‐h diary	5	7	1983	50.00	86.21	4	Defined: breastfed, first born	Breastfeeding only; then 40–45 introduced formula	50	58	44 White, 3 Black; 3 Chinese/Oriental
Jordan et al. (2020)	2020	Canada	24‐h diary	NR	7	2015–2017	NR	NR	3	Whole	NR	10	30	White: 90%; White/Hispanic: 10%
Keller et al. (1996)	1996	Germany	24‐h diary	5	3	1990–1991	NR	NR	4	Defined: firstborn	NR	13	38	Mothers: 100% White
Keller et al. (1998)	1998	Germany	24‐h diary	5	3	NR	70; NR	82; 58;	2	Defined: firstborn	NR	62	—	
Killerby (1992)	1992	UK	24‐h diary	5	1	1989–1990	NR	NR	2	Defined: selected for crying and not crying	Mixed	14	57	
Kivijärvi et al. (2004)	2004	Finland	24‐h diary	5	1	NR	NR	NR	3	Defined: socioeconomic minimum education	NR	56	66	
Korja et al. (2008)	2008	Finland	24‐h diary	NR	3	2001–2002	55.00	94.74	4	Defined	NR	36	47	
Kramer et al. (2001)	2001	Canada	24‐h diary	NR	3	1998–1999	NR	92.91	3	Defined: breastfeeding	Breastfeeding initially	183	—	
Lam et al. (2010)	2010	Canada	24‐h diary	5	7	NR	67.88	64.52	4	Whole	NR	33	42	Mother's ethnicity: 25% Asian, 12% Filipino, 12% Other %; White—52%
Lee (1994)	1994	South Korea	24‐h diary	5	1	NR	NR	NR	0	Whole	NR	32	—	
Lee (2000)	2000	South Korea	24‐h diary	5	1	1996–1997	NR	NR	0	Whole	NR	143	—	
Litmanovitz et al. (2014)	2014	Israel	24‐h diary	NR	3	2009–2011	NR	72; 60	4	Defined: feeding type	Separate groups	35	42	
Lohaus et al. (2001)	2001	Germany	24‐h diary	5	3	NR	NR	100	4	Defined: firstborn	NR	20	45	
Lucas and St. James‐Roberts (1998)	1998	UK	24‐h diary	5	3	NR	NR	94; 78; 95.5	4	Defined	Separate groups	92	48	White: 63%
Lucassen et al. (2003)	2003	NL	24‐h diary	10	7	1994–1997	NR	NR	0	Defined: feeding type	Majority bottle feeding	90	—	
McGlaughlin and Grayson (1999)	1999	UK	CPQ	NA	NA	NR	NR	57.14	0	Defined: Crying	NR	40	—	
McRury and Zolotor (2010)	2010	USA	24‐h diary	5	3	2005–2006	3.62	68.6; 31; 31; 33; 27;	3	Whole	NR	35	—	
Meijer and van den Wittenboer (2007)	2007	NL	CPQ	NA	NA	NR	NR	NR	4	Defined: firstborn	Mixed	86	49	
Miller et al. (1993)	1993	Canada	24‐h diary	NR	7	NR	NR	77.88	4	Define: firstborn	Most breastfeeding	88	50	Mothers: White 72%
Miller‐Loncar et al. (2004)	2004	USA	24‐h diary	15	3	1998–2000	57	NR	3	Whole	Mixed	43	38	92% White; 8% hispanic
Mohebati et al. (2014)	2014	Mexico	CPQ	NA	NA	2000–2002	NR	67	4	Defined	Majority breastfeeding for early points	204	53	
Öztürk Dönmez and Bayik Temel (2019)	2019	Turkey	24‐h diary	15	7	2012–2014	NR	77.78	4	Defined: first born	Majority breastfeeding	21	52	
Popp et al. (2019)	2019	Germany	24‐h diary	NR	3	NR	81.67	61.90	4	Define: firstborn	NR	13	56	
Shinohara and Kodama (2012)	2012	Japan	24‐h diary	5	3	NR	50	100	3	Whole	Mixed	31	52	
St. James‐Roberts and Conroy (2005)	2005	UK	24‐h diary	5	3	NR	NR	94.6; 90	3	Whole	Mixed at discharge, then NR	88	—	Mothers: 88% White
St. James‐Roberts and Menon‐Johansson (1999)	1999	UK	24‐h diary	5	3	NR	NR	56; 80; 80;	0	Whole	NR (recorded)	14	—	
St. James‐Roberts and Plewis (1996)	1996	UK	24‐h diary	5	1–3	NR	92.17	61; 46.5; 34; 36.5	2	Whole	NR (recorded)	122	46	
St. James‐Roberts et al. (1993)	1993	UK	24‐h diary	5	1	NR	NR	NR	2	Whole	Mixed	32	50	
St. James‐Roberts et al. (2003)	2003	UK	24‐h diary	NR	3	NR	NR	68.38	4	Whole	Majority breastfeeding	93	51	Mothers/fathers: White: 88%
Stifter et al. (2003)	2003	USA	24‐h diary	5	4	NR	NR	88.8	4	Whole	NR	128	42	White: 98%
Wake et al. (2006)	2006	Australia	24‐h diary	5	1	1998–2000	68.00	92, 89, 88	4	First born	NR	446	49	
Walker and Menahem (1994)	1994	Australia	24‐h diary	NR	1	1987–1989	NR	53.85	3	Whole	Majority breastfeeding	21	48	
Milgrom et al. (1995)	1995	Australia	24‐h diary	5	7	NR	NR	91, 66	4	Defined: depression	Majority breastfeeding, then NR	63	51	
Wurmser et al. (2006)	2006	Germany	24‐h diary	5	5	NR	NR	68; 67	4	Whole	NR (recorded)	64	52	

^a^ Manali sample included; NL, the Netherlands; Res., resolution of diary (5 mins, 15mins), Recruit., recruitment %, Part., participation %; Char., characteristics of sample reported (from four items socioeconomic status, parity, infant sex, and maternal age); population selection (whole vs. defined population); SS, sample size: from first recorded time point: NA, not applicable; NR, not reported.

### The traditional cry curve model: Double exponent

Importantly, our formalization explicitly includes a representation of the age at which peak cry duration occurs. The model also allows us to make inferences beyond age of peak cry duration. Other characteristics of the cry curve include the magnitude of the peak (i.e., maximum time spent crying per day), the rate of increase or decrease in crying over time, and the lower asymptote of the curve (i.e., the lower bound on crying throughout the modeled time period). Our modeling can also quantify our uncertainty in these estimates, given the data available.

To specify the model, we assume that cry duration data at each measurement follows the gamma distribution:

(1) $$\mu^{\mathrm{Study}}∼\mathrm{Gamma}\left(\alpha ,\beta \right),$$

where *µ* ^Study^ is the mean cry time reported for an individual measurement, and *α* and *β* are the shape and rate parameters of the distribution. We choose the gamma distribution, because unlike the normal distribution, it is restricted to non‐negative numbers (see Figure S4). We re‐parameterize the gamma distribution in terms of its mode and standard deviation, such that:

α=1+μβ,

(2) $$\beta =\mu +\frac{\sqrt{\mu^{2}+4\sigma^{2}}}{2\sigma^{2}},$$

where *µ* is the inferred cry time in the model, and *σ* represents uncertainty in the estimate *µ*. The uncertainty parameter *σ* is unknown and assumed to follow a gamma distribution.

(3) $$\sigma ∼\mathrm{Gamma}\left(0.01,0.01\right).$$

We model change in crying time *µ* using a double exponent model. The double exponent model represents a mixture of rise and decay processes, with separate parameters governing the increase in cry duration to a peak, followed by steady decay (see Figure S5):

(4) $$\mu =A_{0}+Ae^{‐t\gamma_{\mathrm{decay}}}+Ae^{‐t\gamma_{\mathrm{rise}}}.$$

In the model, *A*_0_ represents the lower asymptote, or minimum cry duration. The *γ* parameters represent the rate of decrease (*γ*_decay_) and increase (*γ*_rise_) in duration across the modeled period. The parameter *A* is a scaling parameter, which regulates the height of the curve, and gives an indication of magnitude (not age) of the peak cry duration. The parameters *A* and *A*_0_ are non‐negative, and so are also assumed to follow the gamma distribution:

$$A∼\mathrm{Gamma}\left(0.01,0.01\right),$$

(5) $$A_{0}∼\mathrm{Gamma}\left(0.01,0.01\right).$$

The parameters *γ*_rise_ and *γ*_decay_ are rates, and so are assumed to follow a uniform distribution:

$$\gamma^{\mathrm{rise}}∼\mathrm{Uniform}\left(0,1\right),$$

(6) $$\gamma^{\mathrm{decay}}∼\mathrm{Uniform}\left(0,\gamma^{\mathrm{rise}}\right),$$

with the decay rate (i.e., the rate at which crying decreases over developmental time) assumed to be lower than the rise rate (i.e., the rate at which it increases). From here it is possible to define peak crying time as the first derivative of the model, solved for $\frac{\mathrm{dy}}{\mathrm{dt}}=0$.

Thus, we infer the age at which crying time peaks as:

(7) $$t^{\mathrm{peak}}=\frac{\ln\left(\gamma^{\mathrm{decay}}\right)‐\ln\gamma^{\mathrm{rise}}}{\gamma^{\mathrm{decay}}‐\gamma^{\mathrm{rise}}}.$$

### The change‐point detection model

We tested an alternative, plausible model, a change‐point detection model* (adapted from Lee & Wagenmakers, 2013) to examine the evolution of mean cry/fuss duration in time as a two‐part process: starting at a constant rate, then after a specific time point, changing to an exponential decay. The strength of the change‐point detection model is that it can infer from the data, the point in time at which the system switches dynamics, therefore allowing us to compare the inferred time at which cry duration starts decreasing in this model with the *cry peak* of the double exponential model.

As for the double exponential model, mean cry/fuss time is assumed to follow a gamma distribution, and is re‐parameterized in terms of its mode *µ* and standard deviation *σ* (identical to Equation (1), (2), (3)). In addition, the uncertainty parameter *σ* is also assumed to be constant across both processes (higher cry duration, lower cry duration). To constrain the model, the change‐point parameter *χ* is assumed to follow a uniform distribution, bounded between 0 and *t*_max,_ the maximum age available in the dataset (52 weeks).

(8) $$\chi ∼\mathrm{Uniform}\left(0,\;t_{\max}\right).$$

If the age (in weeks) of infants in a study is below *χ*, the mode cry duration *µ* is equal to the constant *µ*_initial_. If it is above *χ*, it follows an exponential decay with rate *τ*, starting at *µ*_initial_ and with lower asymptote *A*_0_.

(9) $$\mu =\left\{\begin{array}{c} \mu_{\mathrm{initial}}\;\;\;\;\;\;\;\;\;\;\;\;\;\;\;\;\;\;\;if\;t<\;\chi \\ A_{0}+Ae^{‐t\tau }\;\;\;\;\;\;\;\;if\;t\geq \;\chi \end{array}\right..$$

The parameters *µ*_initial_ and *A*_0_ are assumed to follow a gamma distribution, while *τ* is assumed to follow a uniform distribution.

$$\mu_{\mathrm{initial}}∼\mathrm{Gamma}\left(0.01,0.01\right),$$

$$A_{0}∼\mathrm{Gamma}\left(0.01,0.01\right),$$

(10) $$\tau ∼\mathrm{Uniform}\left(0,\;1\right).$$

These two models were applied to both simulated data, and to our meta‐analytic data. We also included two simpler models: a decay only model (i.e., $\mu =A_{0}+Ae^{‐t\gamma_{\mathrm{decay}}}$), and a linear model with a decrease in crying over developmental time.

Bayesian inference (implemented using Gibbs sampling in JAGS via the R2jags package; Plummer, 2003, 2019; Su & Yajima, 2021) was used to infer model parameters. Three chains of 10,000 samples were used, with the first 5000 samples discarded as burn in. Initial values were randomized. These settings were constant across models. For model comparison, we report the deviance information criterion (DIC), where lower DIC represents a better model fit (Spiegelhalter et al., 2002), and Akaike weights, representing the conditional probabilities of a finite set of models given the data (Wagenmakers & Farrell, 2004).

## RESULTS

**FIGURE 1:**
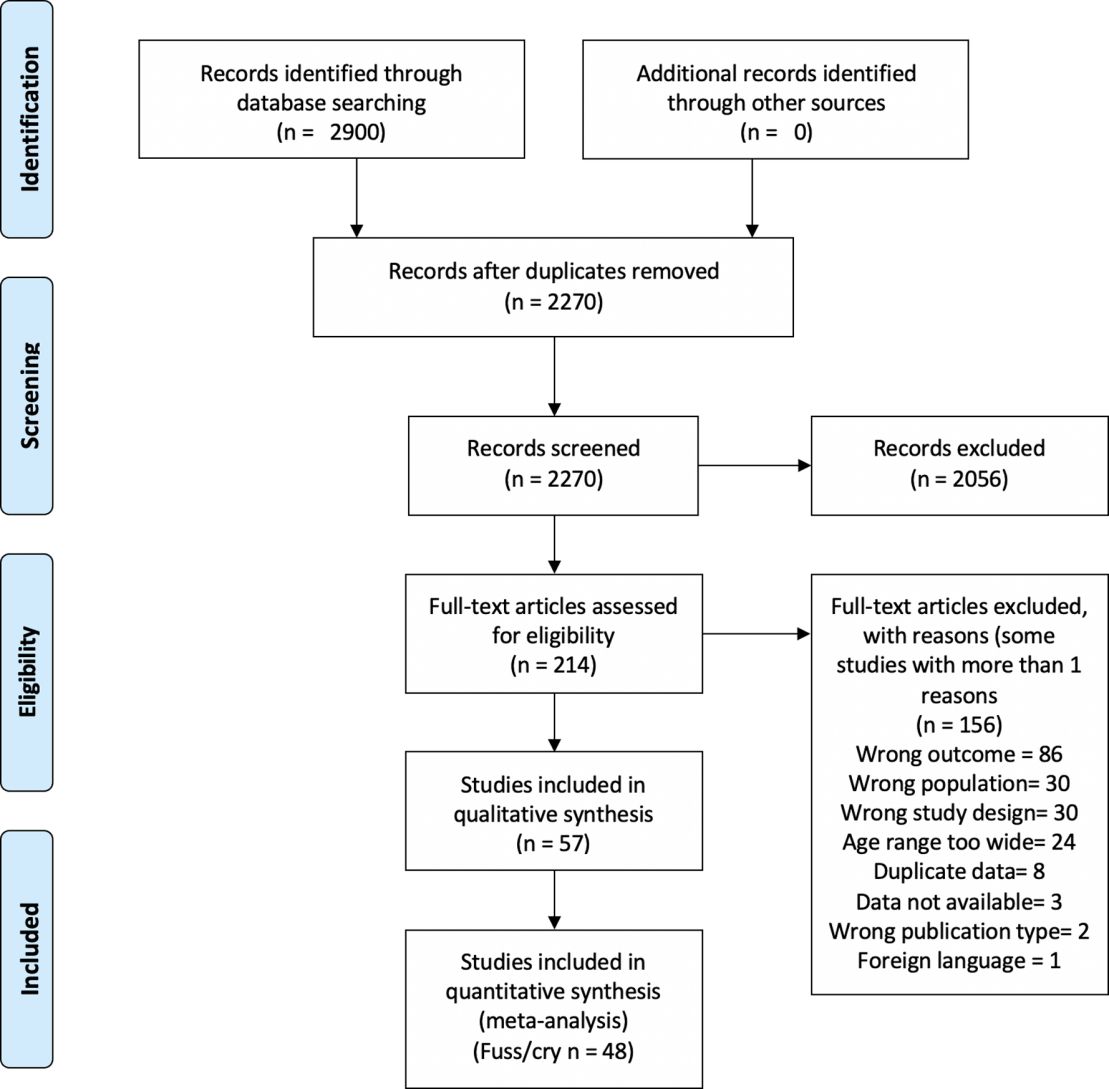
PRISMA flow diagram

Figure 1 presents the PRISMA flow diagram for study inclusion (see OSF folder for individual study exclusions at Full‐Text screening: https://osf.io/dzhvm/). We included data from 57 studies (see Table 1 for full study characteristics). The median study sample size at the first time point assessed was 70 (IQR = 36–122) with a minimum of 10 (Jordan et al., 2020) and a maximum of 1857 participants (Fujiwara et al., 2012). The median study publication year was 2004, with the earliest study published in 1986, and the most recent in 2020.

**FIGURE 2:**
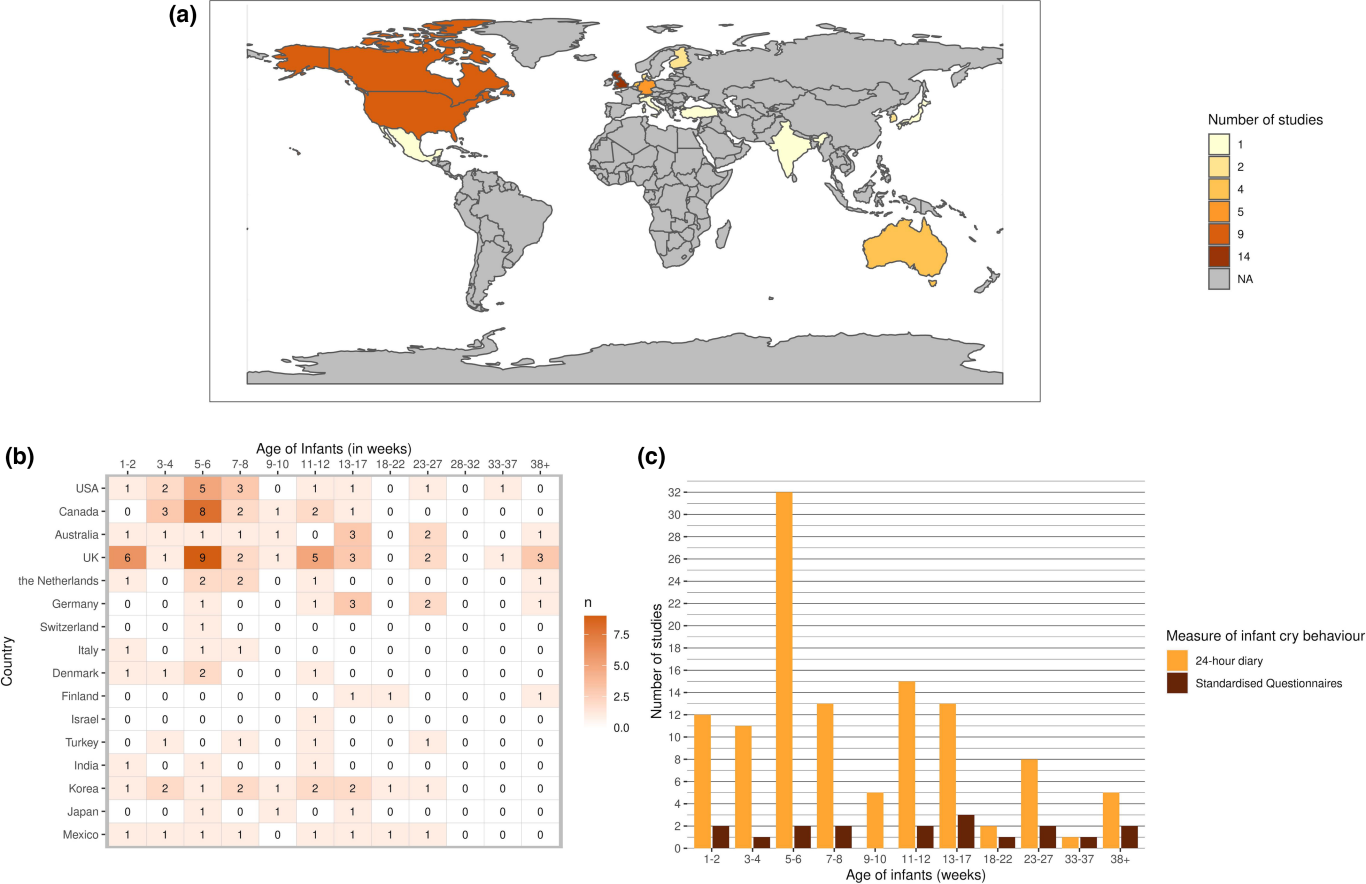
(a) Global map presenting the number of available studies from each country. (b) Numbers of studies providing data per age interval and per country. (c) The number of studies conducted at each age interval, by questionnaire and diary measures

Data were drawn from 17 countries (the United States, the United Kingdom, Canada, Australia, Denmark, Switzerland, Italy, the Netherlands, Germany, Finland, Japan, South Korea, Israel, Mexico, Turkey, India, Sweden, see Figure 2a). Most of the studies were from Canada (*k* = 9; Barr et al., 1989; Clifford et al., 2002a, 2002b; Fujiwara et al., 2012; Hunziker & Barr, 1986; Jordan et al., 2020; Kramer et al., 2001; Lam et al., 2010; Miller et al., 1993), the United States (*k* = 9; Anzman‐Frasca et al., 2013; Atella et al., 2003; Blum et al., 2002; DeLeon & Karraker, 2007; Fujiwara et al., 2012; Geeraerts et al., 2020; McRury & Zolotor, 2010; Miller‐Loncar et al., 2004; Stifter et al., 2003) and the United Kingdom (*k* = 14; Baildam et al., 1995; Bilgin & Wolke, 2020a; Darlington & Wright, 2006; Harrison, 2004; Killerby, 1992; Lucas & St. James‐Roberts, 1998; McGlaughlin & Grayson, 1999; St. James‐Roberts & Conroy, 2005; St. James‐Roberts & Menon‐Johansson, 1999; St. James‐Roberts et al., 1993, 1994, 2001, 2003, 2006).

### Infant characteristics

**FIGURE 3:**
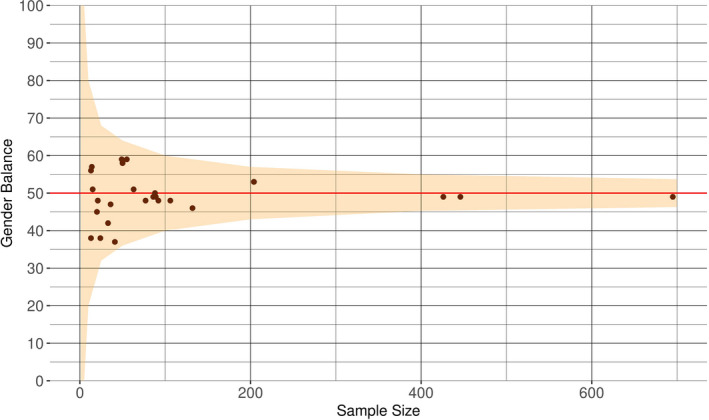
Distribution of gender across study samples. The relation between sample size and male to female proportion (gender balance). A gender balance of “0” indicates a male‐only sample, while a gender balance of 100 indicates a female‐only sample. The orange area represents the theoretical sampling error for a given sample size, obtained through simulations (*n* = 10,000 for sample sizes of 1, 5, 10, 25, 100, 200, 400, 700, with a true proportion of 50%, indicated by the red line). The orange shaded region represents the 5% and 95% quantiles of gender balance for a given sample size and the brown dots represent the available data

The youngest participants were aged 1 week (Lohaus et al., 2001; McRury & Zolotor, 2010; Mohebati et al., 2014; St. James‐Roberts & Menon‐Johansson, 1999; St. James‐Roberts et al., 2001). The oldest participants were aged 52 weeks (Baildam et al., 1995; Keller et al., 1998; Kivijärvi et al., 2004; McGlaughlin & Grayson, 1999; Meijer & van den Wittenboer, 2007; Wake et al., 2006). Most studies reported on infant measures at the 5‐ to 6‐week interval (*k* = 35, see Figure 2b). The majority of studies (44/57) reported on the overall sex/gender composition of the sample. Overall, there was approximately an even proportion of male and female infants included (49% female, see Table 1). We performed further exploratory analysis of the gender composition of our data, and observed that the variation across samples was within the range of sampling error (see Figure 3). As would be expected, larger participant sample sizes were associated with samples closer to 50% male and female infants. However, we also found no evidence of bias toward more male or female participants for studies with small sample sizes (<200 participants). Independent age groups were closely balanced, except for the age interval 33–37 weeks (37% female), where just one study with 41 participants was included. Ethnicity of the infants included was sparsely reported overall (8 studies reporting infant ethnicity, 10 reporting maternal/parental ethnicity). Where ethnicity was reported, this was typically given as the majority ethnicity, which was “White” in all studies (see Table 1).

### Measures and study design

The majority of studies used the Barr Baby 24‐h diary (see Figure 2c; one study used the Infant Sleep Activity Record, a 24‐h diary; Öztürk Dönmez & Bayik Temel, 2019) or a modified version with a time resolution of 10 or 15 min (*k* = 8) instead of the standard 5 min (*k* = 34, 10 studies did not report the time resolution). The median number of diary days was 3 (range 1–10 days). Most studies also specified that mothers completed the diaries (*k* = 35), and none included data from fathers reporting on their infants’ crying specifically. Four studies used the Crying Pattern Questionnaire, the CPQ (Bilgin & Wolke, 2020a; Meijer & van den Wittenboer, 2007; Mohebati et al., 2014; St. James‐Roberts et al., 1994). McGlaughlin and Grayson (1999) used a modified version of the CPQ, assessing crying only, rather than “crying and fussing” as in the standard CPQ. Mohebati et al. (2014) also adapted the CPQ to the country‐specific appropriate time periods.

Most of the included studies combined crying and fussing into one category of “cry/fuss” (*k* = 26). A number of studies also measured and included “inconsolable crying” in their outcome, which we refer to as “total distress” (*k* = 4). Several studies reported on crying only (*k* = 5), specifying that they excluded fussing (Baildam et al., 1995; Harrison, 2004; Litmanovitz et al., 2014; McGlaughlin & Grayson, 1999; Walker & Menahem, 1994) or reporting crying only alongside fussing, and inconsolable crying (*k* = 22). When studies reported crying and fussing separately, these were summed and added to the category of “cry/fuss” (*k* = 23). If inconsolable crying was reported separately alongside with cry and fuss, then these were summed to be included in the “total distress” category (*k* = 11). The majority of studies were longitudinal studies (*k* = 35), with a smaller number comprising cross‐sectional studies (*k* = 10), intervention trials (*k* = 10) and two case‐control studies. A minority of studies reported on the timing of their data collection, and of these, most collected data in the early 2000s (see Table 1).

### Synthesizing cry duration measures

**FIGURE 4:**
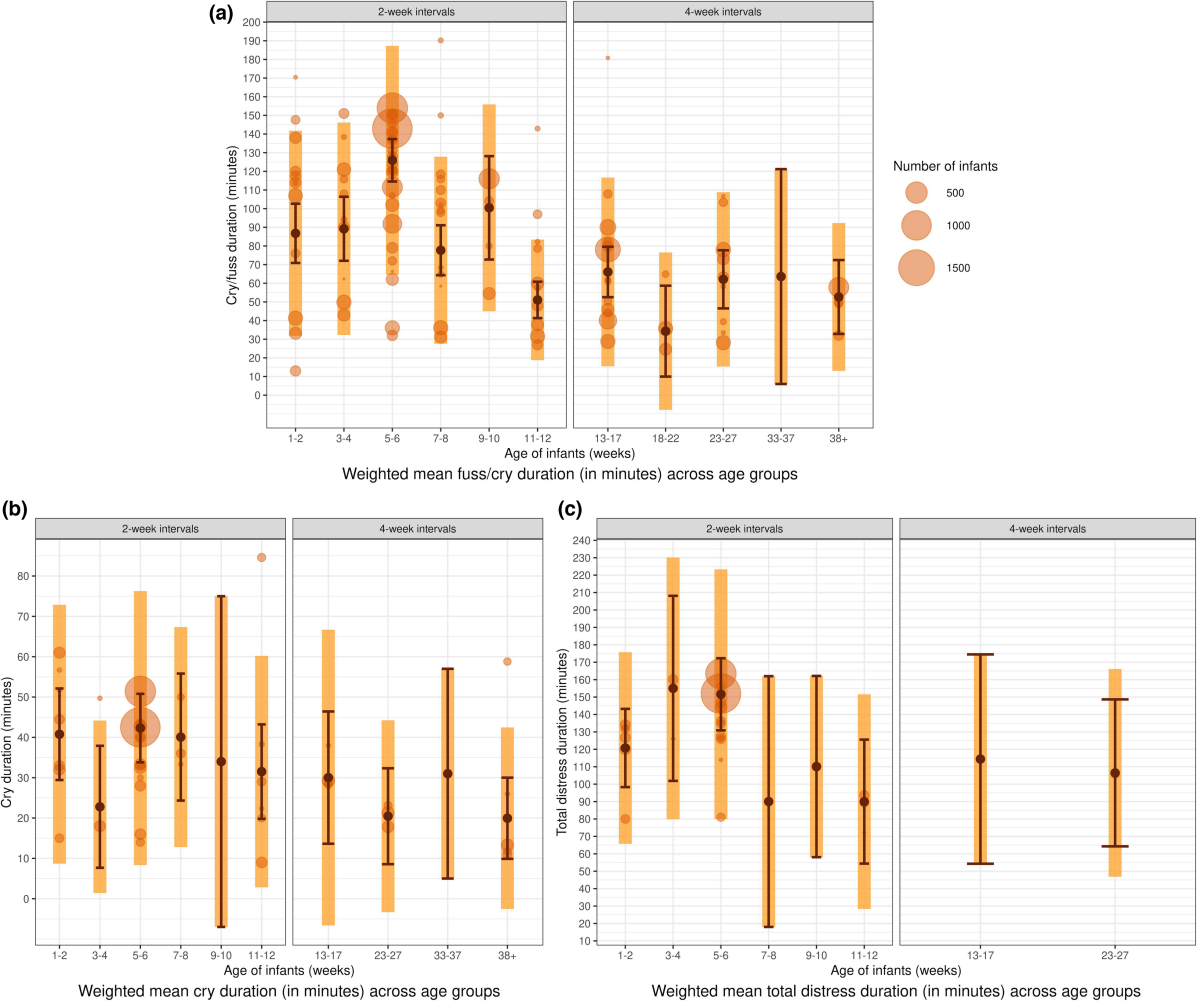
Weighted mean duration for three measures of infant cry duration: (a) cry/fuss; (b) cry only; (c) total distress. Each panel combines country‐level data across ages. Orange circles represent mean durations of individual samples within available studies. The circle size reflects the number of infants and is used to visualize that influence of a given sample in the calculation of the weighted means. The light orange bar represents the standard deviation of age intervals, pooled across available samples. The dark circles represent the weighted mean of each age interval, and the error bar represents its standard error

We calculated weighted means per age interval for three cry duration measures (i) fuss and cry combined (cry/fuss) (ii) only crying, (iii) fussing and inconsolable crying (total distress). Means were weighted by number of participants to reduce the bias induced by studies with small sample sizes (see Figure 4). The linear regressions to assess the effect of age used raw means and followed the structure: formula = Mean minutes ~ Age interval.

**FIGURE 5:**
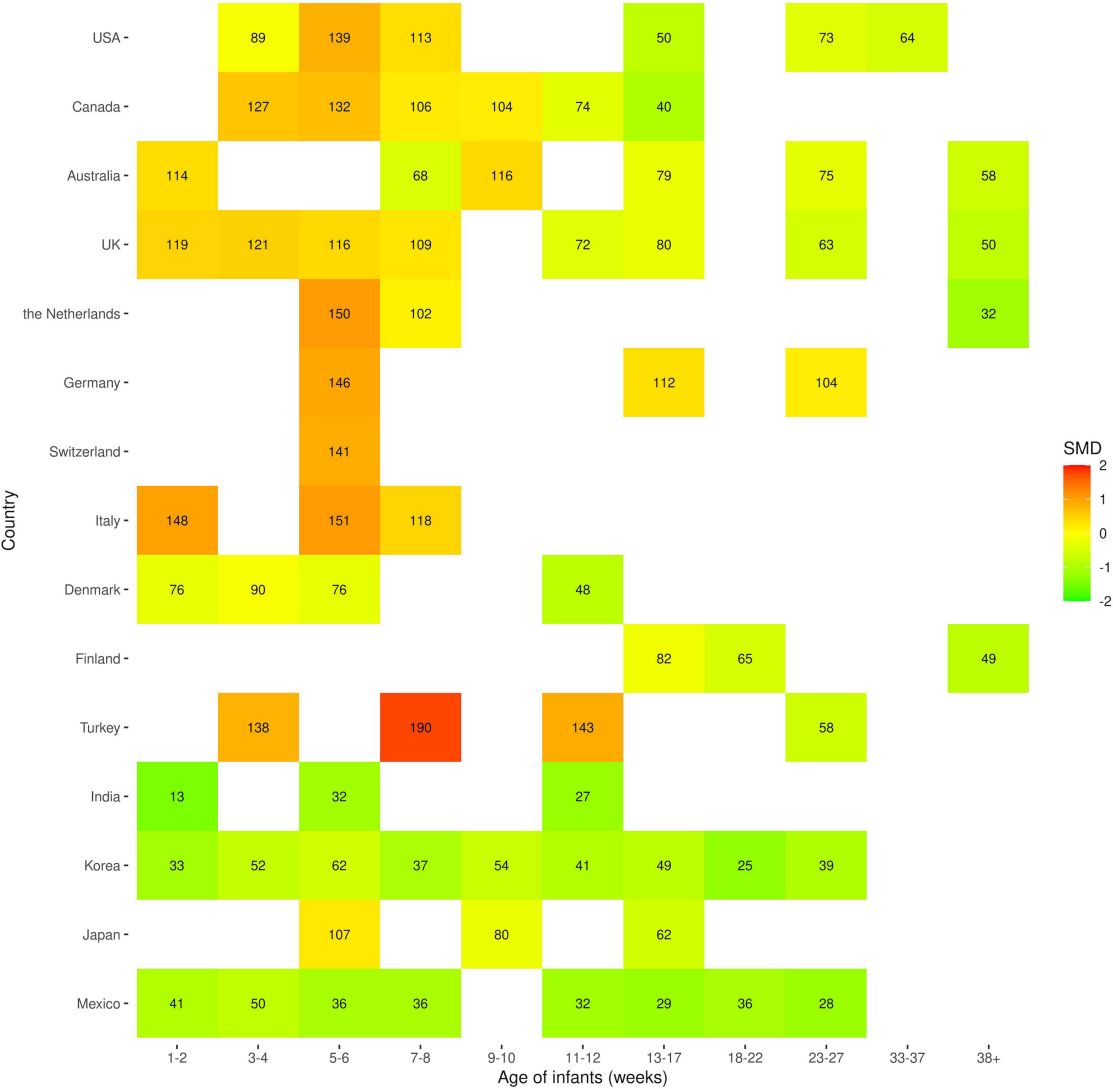
Weighted mean of parent‐reported cry/fuss duration across the infant age intervals and across all available countries (blank cells represent ages and countries with no available data). The standardized mean difference (SMD) is calculated by comparing the weighted mean of each cell to the overall weighted mean across age intervals and countries, providing an overview of the effect of both age and country on cry/fuss duration

(i) For infant cry/fuss, the overall weighted mean was approximately 93 min (pooled *SD* = 54.53). The highest duration was at 5–6 weeks (126 min, *SE* = 11.39). The lowest value was for 18–22 weeks at 34 min (*SE* = 24.36). Figure 5a shows two different clusters of age intervals with broadly comparable durations (1–10 weeks; 11–38+ weeks). Cry/fuss was recorded as longer in duration at the age intervals until 9–10 weeks, with mean estimates ranging from 78 to 126 min. From 11 to 12 weeks to 38+ weeks, mean estimates were between 34 (18–22) and 66 (13–17) min, and there were fewer available studies with smaller sample sizes (Figure 4a). The large *SE* at 33–37 weeks can be explained by the presence of only one sample of infants. The regression analysis using unweighted mean durations confirmed this pattern of results, with the intercept (1–2 weeks) estimated at 99.32 min (*SE* = 10.50, *t*(101) = 9.46, *p* < .001). The durations at age intervals 11–12, 18–22, 23–27, and 38+ weeks were all significantly lower than the intercept (*b* values, −34.5 to 57.6; *p* values = .03–.01, see Supporting Information). In summary, the 5‐ to 6‐week interval had the longest duration of cry/fuss, after 11–12 weeks, there were broadly lower overall averaged cry/fuss durations, compared to the overall weighted and unweighted means.

The weighted mean duration of (ii) “crying only” across all age intervals was approximately 38 min (pooled *SD* = 32.32). The highest mean duration occurred at 5–6 weeks (approx. 42 min, *SE* = 8.49; see Figure 4b), with similar estimates at 1–2 weeks (41, *SE* = 11.34) and 7–8 weeks (40, *SE* = 15.74). The lowest mean duration was estimated at 38+ weeks (20, *SE* = 10.06) and 23–27 weeks (20, *SE* = 10.06). Only 1 study reported on infant crying only for 9–10 weeks (34, *SE* = 41) and 33–37 weeks (31, *SE* = 26), explaining the large standard error. For the regression analysis using unweighted means, the intercept (1–2 weeks) was estimated at 43 min (*SE* = 5.30, *t*(41) = 8.11, *p* < .001). Only the mean durations from age intervals 23–27 weeks (*b* = −21.21, *SE* = 9.18, *t*(41) = −2.33, *p* = .02) and 38+ weeks (*b* = −18.84, *SE* = 8.55, *t*(41) = −2.20, *p* = .03) were significantly lower than the intercept.

(iii) Of the three cry measures, total distress had the least available data (*k* = 28), explaining the high variability of the weighted mean durations (Figure 4c). Three age intervals had just one sample, at 7–8, 9–10, and 13–17 weeks, while 5–6 weeks comprised 43% of the samples available (*k* = 12). The highest mean duration occurred at 3–4 weeks with 155 min (*SE* = 53.14) and the lowest occurred at 11–12 with 90 min (*SE* = 35.59). The results of the regression set the intercept (1–2 weeks) at 121 min (*SE* = 8.32, *t*(20) = 14.52, *p* < .001) and only the age interval 11–12 appears to be significantly lower (*b* = −35.86, *SE* = 14.41, *t*(20) = −2.49, *p* = .02).

### Cross‐country comparisons: Cry/fuss

We performed subgroup analyses using random effects models for the age intervals with more than 10 studies, as per recommendations (Higgins et al., 2003). For all age intervals assessed, we found significant subgroup effects (6 intervals: 1–2, 3–4, 5–6, 7–8, 11–12, 13–17; *Q* values: 78.91–452.36; *p* values all <.0001). We particularly note countries with 95% CIs for their standardised mean difference estimates that did not overlap with the overall estimate CIs for the given age interval. At 1–2 weeks, three non‐Western countries (India, South Korea, and Mexico) had mean cry/fussing durations of 13, 33, and 41 min, whereas the Western countries ranged from 76 min for Denmark to 148 min for Italy (see Figure 5). Indeed, across all available age intervals, country data from India, South Korea, and Mexico was lower than the averages, with the most pronounced differences for the intervals before 12 weeks. However, Mexico and India were represented by one study each, and two studies were used to calculate the average cry/fuss time of South Korea. Estimates of cry/fuss from Germany and Turkey appeared to be higher than the pooled estimates from other studies, but Turkey was represented by a single study also (Öztürk Dönmez & Bayik Temel, 2019). From the Western countries included, Danish infants had lower recorded cry and fuss durations based on the two available studies (Alvarez, 2004; St. James‐Roberts et al., 2006).

Focusing on 5–6 weeks, the age interval with most available data, the United Kingdom, the United States, and Canada, clustered at around 120 min of estimated infant crying (25 samples were included from these 3 countries), while the Netherlands, Switzerland, and Germany (4 samples) had slightly higher estimated averages (146–151 min). Similar to the 1‐ to 2‐week interval findings, Korea, Denmark, Mexico, and India all had lower estimates (non‐overlapping CIs) than the age interval pooled estimate, but the Netherlands had a higher estimate (see Supporting Information for full model results). As seen in Figure 5, cross‐country comparison data are sparse at 9–10 weeks, and only a small number of countries contribute data, particularly for older infants (33 weeks onwards).

### Heterogeneity of estimates

At each age interval, there was significant heterogeneity in cry/fuss duration estimates, with I2 values ranging between 82.5% for the 38+ weeks age interval, to 97.5 for the 1–2 weeks age interval. The statistical heterogeneity present is at least partially related to between‐country differences, as illustrated in Figure 5, where three countries (Mexico, India, and South Korea) represented by four studies represented more extreme outliers, with considerably lower cry/fuss durations reported at all assessed age intervals. We used the dmetar R function to detect outliers, whereby the study's confidence interval did not overlap with the confidence interval of the pooled effect at each age interval. This means the study's effect size estimate is unlikely to be part of the "population" of effect sizes pooled in the present meta‐analyses (i.e., the individual study differs significantly from the overall effect, Harrer et al., 2019). With this approach, we noted outliers at all age intervals, with the exception of weeks 9–10, 18–22, and 38+ (see Supporting Information for all of the identified outliers). Only for weeks 3–4 (Canada, Korea, Mexico; Kramer et al., 2001; Lee, 2000; Mohebati et al., 2014) and 7–8 (Turkey, Korea, Mexico; Lee, 2000; Mohebati et al., 2014; Öztürk Dönmez & Bayik Temel, 2019), did outlier removal reduce heterogeneity to the “moderate” rather than high range, from 95% and 95.5% to 69.5%, and 62.1%, respectively.

### Moderator analyses

We performed moderator analyses for two variables, study quality rating and feeding type, using univariate meta‐regression analyses. We found no evidence of an association between study quality rating and infant cry/fuss duration averages across any of the age intervals with sufficient data available (see Supporting Information, all *p* values >.31; minimum *k* of 10; age intervals 1–2, 3–4, 5–6, 7–8, 11–12, 13–17). For feeding type, we selected studies with cry/fuss duration data and information on feeding type, providing 60 samples from 30 different studies. Using the 5‐group feeding categorization (majority breastfeeding; majority bottle‐feeding; breastfeeding only, bottle‐feeding only, mixed sample), exactly half of the samples fell in the “Mixed” category, while only three samples comprised “bottle fed” only infants. The availability of information on feeding type decreased with infant age, as well as the diversity of types reported (see top panel of Figure S3b). Therefore, any association identified between cry/fuss duration and feeding type is likely to be a spurious association, reflecting the relation between age and cry/fuss duration identified in the previous section (see bottom panel of Figure S3b).

As for the subgroup analyses of country, we took age intervals with a minimum of 10 studies, which left 2 age intervals with sufficient data to compare feeding types (1–2 weeks with *k* = 11; 5–6 weeks with *k* = 19). Given the imbalanced numbers between categories, we are cautious in interpreting these results, as effects, or their absence, may be a result of insufficient data (see 1–2 and 5–6 weeks; bottom panel Figure S3b). We did not find any significant difference between Feeding types at age 5–6 weeks. At age 1–2 weeks, “bottle only” was flagged as significantly higher than the overall mean cry/fuss duration at that age. However, we note that with a single data point, we lacked the variance necessary to estimate a significant difference (see 1–2 week, bottom panel of Figure S3b). We conducted post hoc analyses to investigate the association between crying time and sex/gender. Given the limited data on each individual age group, we conducted univariate linear regressions on all data available, before and after adjusting for age group and sample size. No significant effect of gender was found examining the raw mean (*b* = −1.80, *SE* = 1.14, *t*(35) = −1.58, *p* = .12, *R* ^2^ = .07), or the weighted means (*b* = 0.09, *SE* = 0.74, *t*(35) = 0.13, *p* = .90, *R* ^2^ = .00).

### Publication bias

We did not find evidence for publication bias when inspecting the asymmetry of the distributions of ESs, examining cry and fuss estimates using funnel plots (see Figure S2). The Begg and Mazumbar rank correlation test (Kendall's *τ*) was employed to examine whether the observed outcomes and the corresponding sampling variances were correlated. The test did not reach statistical significance, Kendall's *τ* between −1 and 1 and *p* values between .26 and 1.00, indicating no significant evidence of funnel plot asymmetry for any of the age intervals tested. Results from the Duval and Tweedie “trim and fill” method are presented in Table S4, where only a single study was added to the left side of the funnel plot at age interval 1–2 weeks.

### Modeling of the cry curve

The simulation study and sensitivity analysis suggest reasonable parameter recovery for all parameters within the double exponential model, excluding the scaling parameter *A* (see Figures S6 and S7). Thus, caution should be exercised when using this parameter in inference, especially for lower values (see Figure S8). While the magnitude of crying time is dependent on A, the age of peak cry duration is independent of it (Equation 4 and 7), so it is, therefore, possible to reliably identify when infants cry the most. For the change‐point detection model, parameter recovery for *µ*_intial_ and *A*_0_ was reasonable. For *χ*, the change‐point parameter, recovery was good for values below 20, but systematically underestimated for values above 20 when the simulated data presented a similar bias towards the age of infants (see Figures S9 and S11). This problem was resolved when the simulated data were more balanced across ages (see Figure S10). This suggest that, if the change‐point is expected after 20 weeks of age, more data on older infants would be needed in order to reliably estimate it. However, a transition later than 12 weeks is very unlikely, thus in this context, we expect to reliably infer *χ*. Parameter recovery for the rate of decay *τ* was poor, suggesting that the model's results do not provide a reliable estimate of how fast cry duration transitions from *µ*_initial_ to *A*_0_ after the change‐point (see Figure S9).

We fitted the models to the cry/fuss data, which was reported across the majority of studies and is widely understood as synonymous with “crying duration” (e.g., Wolke et al., 2017). Model comparison suggests that both the double exponent model (DIC = 1145.9, weight = 0.29) and the change‐point detection model (1144.3; weight = 0.65) were better fits to the data than both the simple decay (DIC = 1150.1, weight = 0.03) and linear decrease models (DIC = 1151, weight = 0.02). These DIC values suggest that the double exponent model and change‐point detection model are clearly different from the two “control” models, but are only minimally different from one another. Evidence ratios using model weights indicate that the double exponential model is 9.7 times more likely to be a better representation of the data than an exponential decay model, and 29 times more likely than a linear decrease. The change‐point model is 20.09 times more likely to be a better representation of the data than an exponential decay model, and 28.5 times more likely than a linear decrease.

**FIGURE 6:**
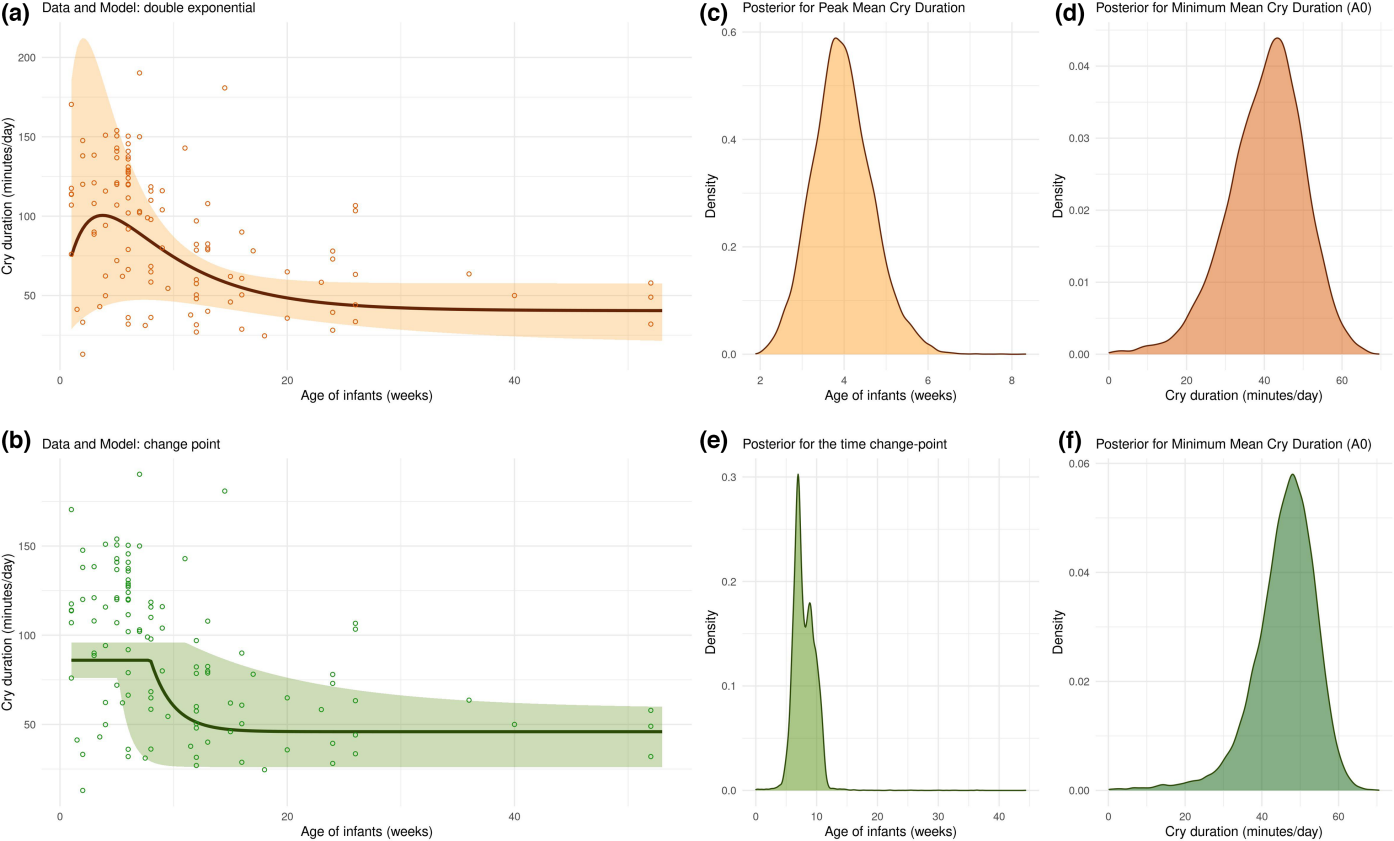
(a, b) Inferred curve representing the change in cry duration across ages, modeled as a double exponential (a) and a change‐point detection (b), fitted using the posterior means of the inferred model parameters (solid curve), with uncertainty presented using 95% Credible intervals (shaded region around the curve), and data points (circles). (c) Posterior distribution of the cry peak, inferred from the double exponential model fitted to the meta‐analytic data. (d) Posterior distribution of *A*_0_, the asymptote of the double exponential model. It represents the inferred theoretical minimum value towards which cry duration decays. (e) Posterior distribution of the time of the change‐point between high and constant cry period and the exponential decay in the change point detection model. (f) Posterior distribution of *A*_0_, the asymptote of the change‐point detection model

We used these two differing models, the traditional cry curve, and the change‐point model to make inferences about the data from the meta‐analysis. For the “cry curve” (see Equation 7), the peak of crying occurred at 3.97 weeks (95% credible intervals: 2.64–5.50 weeks, see full posterior distribution in Figure 6c). Similarly, we can infer the minimum cry duration in *A*_0_, the lower asymptote of the model, at 40.4 min a day (95% Credible Intervals: 19 to 58 min a day, see full distribution in Figure 6d). For the change‐point model, the time point where there was a change from a constant mean to an exponential decay occurred at 7.97 weeks (95% Credible intervals: 5.14–11.06 weeks, see full posterior distribution in Figure 6e). The mean estimated cry duration during the “high and constant” cry period was 85.98 min per day (95% credible intervals: 76.1 to 95.85 min) while the minimum cry duration is estimated at 45.93 min a day (95% credible Intervals: 26.11 to 59 min a day, see full distribution in Figure 6g).

## DISCUSSION

Our synthesis of data from 57 studies, from a total of 17 countries, provides a comprehensive view of the heterogeneity of parent‐reported infant cry behavior, both between and within studies and countries. Perhaps as a consequence of the Brazelton (1962) analysis, which provided a compelling case for a “cry curve” peaking at 6 weeks, the overwhelming majority of studies have focused on the 5‐ to 6‐week period in infancy. Furthermore, three countries have contributed most of the available data: the United Kingdom, Canada, and the United States, primarily driven by two prolific researchers, St. James Roberts and Barr, the latter of which developed the Barr Baby Diary, which dominates infant cry measurement in the field. Entire continents are absent from the current global perspective on infant crying, and where low‐and‐middle‐income countries are represented, it is typically by a single data point.

With the caveats of the synthesized data in mind, there are several findings we would like to highlight. We note substantial heterogeneity in cry and fuss duration reports across countries. Country‐level differences in reports are clearly a contributor to the between‐study heterogeneity estimated here. Inspecting the study‐level data, most infant samples were characterized by large standard deviations in reported cry duration. This between‐study and within‐study variation underscores an important aspect of infant crying: durations of reported infant crying are highly variable. The normal variability from infant to infant is emphasized in psychoeducation materials for parents (e.g., Barr et al., [n.d.]) and our synthesis supports such an emphasis. For infant feeding type, in contrast to Wolke et al. (2017), we found no consistent evidence for a significant difference in cry/fuss duration related to each study's sample composition (majority breastfeeding; majority bottle‐feeding; breastfeeding only, bottle‐feeding only, mixed sample).

### Theoretical and clinical implications

We note several theoretical and clinical implications of our cry duration synthesis and our subsequent cry curve modeling. We present two models of infant crying over time, which are not robustly distinguishable from one another in terms of their fit to the data, but both are better than exponential decay models or linear decline models. Our “cry curve”, with cry duration modeled as a rise and decay process, maps to the original notion of a peak in crying, followed by a decline. While our estimates of the magnitude of the peak are tentative, we provide evidence to suggest it is slightly earlier (4 weeks) than the originally specified 5–6 weeks (Brazelton, 1962). Our change‐point model suggests a “high and constant” level of crying until approximately 8 weeks (mean of 86 means per day), followed by a drop. The two models infer similar minimum cry durations (means: 40–45 min), after the first 8 weeks of life. While our models support different inferences about initial changes in cry duration, they provide clear, testable questions for future work. In addition, our modeling provides a framework for systematically investigating individual and cultural differences in future work (e.g., infants raised with different parenting styles, identifying atypical patterns of crying). We view our model as a tool to allow formal comparisons between infants, or across countries (https://osf.io/dzhvm), which may be used to design or analyze new studies.

Second, for clinicians discussing infant cry durations with parents, we note that the initial “rise” and subsequent “decay” in cry duration are not as dramatic as depicted in many parent‐facing diagrams on infant crying. This is consistent with the analysis presented in Wolke et al., and may support clinicians in managing parental expectations, along with our description of the high variability in typical cry patterns, both within and across countries. Our change‐point model, as for the cry curve model, indicates that daily cry duration remains considerable beyond its initial period of stable and high cry duration (or “peak” for the cry curve model). We also highlight the country‐related variability in infant crying, which might impact both parental and clinician definitions of “excessive” infant crying. Excessive infant crying has been defined in a variety of ways (for review, see Reijneveld & Stronks, 2001), but a duration‐based definition as in the Wessel criteria (Wessel et al., 1954) may capture infants in the upper quartiles in some countries, but not others (see Figure 5). Furthermore, clinical considerations of infant crying as outside the normal range should take into account infant age, as also emphasized by Wolke et al. (2017), but noting that the available data after 12 weeks, indicate that crying remains substantial in daily duration.

### Why are there substantial differences across countries?

As for the Wolke et al. (2017) analysis, there was substantial variability across countries, where one European country, Denmark, seemed to represent an “outlier” relative to the average reported durations in the United Kingdom, the United States, and Canada, which generally had comparable reported cry durations at the different age intervals. We would like to caution about the confidence we should place in this estimate as *robust evidence* for Danish infants crying less than other European or North American infants. Our Danish estimates are based on just two unique studies with non‐overlapping infant ages and relatively small samples, published 15 years ago. While commentators on the Wolke et al. (2017) finding have suggested that Denmark, with its long parental leave, and provision of subsided childcare (Craig & Mullan, 2010), may reduce the stress of early parenting, and therefore lower infant distress (Ramaswamy, 2017), this is characteristic of the Nordic countries generally. Another common parenting practice in Denmark and in Nordic countries involves placing infants to sleep outside (e.g., Tourula et al., 2008), which may also impact parents’ cry reports. Validation studies of Nordic parents’ reports have not yet been conducted, as is the case for most of the 17 countries included here.

Our synthesis included only a small number of studies from non‐Western countries, India, Mexico, and South Korea, with pronounced low levels of reported infant cry durations, relative to clusters of particular anglophone countries (e.g., the United Kingdom, the United States, and Canada). Parental practices around responding to infant crying vary across countries and Western and non‐Western countries have been reported to differ in measurable ways. In India, mothers have been reported to be less inclined to leave their infants to cry, and more likely to take them into their own bed (St. James‐Roberts et al., 1994). In Chile, mothers are reported to engage in frequent infant carrying, and researchers have suggested this reduces instances of crying (Rinne et al., 1990). Even within single European and Western countries, there is a wide range of parental practices and beliefs about infant crying, and controversies about the use of strategies such as “cry it out” (Bilgin & Wolke, 2020b). We also note that infant ethnicity was reported in a small number of the included studies (*k* = 8), and in all cases, the sample compositions were majority White infants, or White parents, limiting the generalizability of the current synthesis.

### Parent reports do not reflect actual infant cry durations

The original validation study for Barr's Baby diary reported correlations between parent reports and audio‐recordings of 0.67 for crying only, 0.45 for crying and fussing, but no correlation for fussing only (0.01; Barr et al., 1988). Some authors have argued that the discrepancy between audio‐recording and diary reports of fussing may reflect the insensitivity of recordings to visible signs of infant distress, which would lead parents to more accurately record infant fussing behavior (St. James‐Roberts & Plewis, 1996). Barr suggested that the typical shorter duration of fussing relative to crying may contribute to the discrepancy between diaries and recordings. Regardless of the sources of differences between diaries and audio‐recordings, our synthesis is of parent‐reports, or perceived infant cry durations rather than actual durations.

While diaries and questionnaires provide feasible methods for assessing large samples, they do not fully correspond with audio‐recordings and are subject to between‐participant variations in recording accuracy, compliance, and memory biases, as for other measures of parent‐reported child behavior (e.g., coughing, Dales et al., 1997). Notwithstanding ethical and privacy concerns with long‐form recordings of the auditory environment (Cychosz et al., 2020), there are established proprietorial infant wearable devices (e.g., Language Environment ANalysis) and emerging open‐source hardware solutions (e.g., Baby Logger, Cao et al., 2018) that provide methods to audio‐record crying from larger infant samples than previously tested. Finally, we note our inclusion of both questionnaire and diary instruments, an extension of the Wolke et al. (2017) study which included diaries only. For the CPQ, the standardized instrument used in studies here, a previous validation reported that the amount of fussing and crying was estimated as 13% higher in the CPQ relative to a 24‐h diary (Wolke et al., 1994), indicating imperfect alignment between measures.

### Limitations of the present synthesis

In the current body of studies, some authors report on “crying,” referring to both “crying and fussing” combined, whereas others deliberately exclude fussing from their reports, ambiguity that we resolved where possible by contacting authors for clarification. We recommend that, for a more complete understanding of cry behavior, it will be of value if authors report on each of the categories (fussing, crying, inconsolable crying) from the Barr Baby Diary separately, for example, in Supporting Information. In addition to the between‐infant variability in our analyses, the available evidence also suggests considerable within‐infant differences, both from day‐to‐day and from week‐to‐week (Barr, 1990a; Rebelsky & Black, 1972). We did not assess within‐infant variation, and our estimates, as in the Wolke et al., (2017) synthesis, treat the observations of cry duration at each time interval as independent.

While the mean and *SD* are the most common summary statistics provided, they may not be the most appropriate descriptive for cry duration data. Using mean and *SD* to represent the data, we are led to assume that crying data are normally distributed, an assumption that is unlikely to be strictly true. The high *SD* values relative to the mean in many studies suggest a non‐normal distribution (cry duration cannot be a negative value, see Figure S5). In extracting study data, we did not find any articles that provided raw infant diary data. Future meta‐analytical studies would benefit from the granularity provided by raw data availability, which would allow descriptions and modeling of the data, beyond the most commonly used statistical approaches (e.g., between‐group comparisons with ANOVAs). Researchers may consider raw data sharing using third party repositories with a persistent link (e.g., a DOI, repository examples include OSF, DYAD, Figshare), or indeed the sharing of synthetic datasets, where there are concerns about sensitive infant‐related data (for further discussions of data sharing practices, see Towse et al., 2020). As has been argued previously, future meta‐analyses will be most productive if they can be conducted synthesizing *individual*‐level data—if such data are accessible (Roisman & van Ijzendoorn, 2018).

## CONCLUSION

The current understanding of infant cry patterns is largely derived from a body of studies focused on infant age 5–6 weeks, and from three anglophone Western counties. The heterogeneity of the pooled estimates calculated could be better understood via sharing of raw data from infant diaries. Raw data availability could allow us to build accurate statistical descriptions of crying and could stimulate the elaboration of new theoretical accounts. While standardized paper‐based diaries have been the dominant method for collecting parent‐reported cry duration data, automated audio recording and experience sampling methods offer new opportunities to collect fine‐grained, and complementary data streams. Raw datasets, from different data recording modalities, could allow us to formalize and test proposed mechanisms (e.g., environmental, or individual‐infant variables) and theories using computational models, as previously done in other fields of research (e.g., sleep, decision‐making). Models can be built to consider variables, such as the broad impact of parents’ preferred soothing strategies, or the developmental stage of the infant, that can reproduce the variation observed across age intervals and cultures. In addition, physiological variables, such as different hormones and their fluctuation (leptin, melatonin, cortisol), and sleep timing, could be integrated to account for individual variation, which may be useful in understanding atypical or problematic cry behavior.

## CONFLICTS OF INTEREST

The authors declare no conflicts of interest.

## Supporting information

Supplementary Material
