# Supplementary material for: Crying in the first 12 months of life: A systematic review and meta‐analysis of cross‐country parent‐reported data and modeling of the “cry curve”
Source: Child Dev. 2022 Apr 19;93(4):1201–22. doi: 10.1111/cdev.13760 (PMC9541248; doi:10.1111/cdev.13760)
Supplement: Supplementary file 1 — Supplementary Material [file CDEV-93-1201-s001.docx]

**Supplementary Materials**

**Results**

# *Differences across Age intervals: ANOVA results*

To replicate the analysis reported in Wolke et al. (2017), we carried out an ANOVA, with age interval as the fixed effect and Tukey HSD post-hoc tests. The results replicate the regression results reported in the main manuscript.

S. Table 1. Results of the ANOVA models with three measures of infant crying duration (“cry/fuss”, “crying only” and “total distress”) and testing age interval effects

| Variable | Predictor | Sum of Squares | df | Mean Square | F | p | Partial η | Partial η  90% CI  [LL, UL] |
| --- | --- | --- | --- | --- | --- | --- | --- | --- |
| Cry/fuss | Mean minutes | 118371.61 | 1 | 118371.61 | 90.26 | .000 |  |  |
|  | Age interval | 51646.17 | 10 | 5164.62 | 3.94 | .000 | .28 | [.10, .33] |
|  | Error | 133769.91 | 102 | 1311.47 |  |  |  |  |
| Crying only | Mean minutes | 14799.52 | 1 | 14799.52 | 67.14 | .000 |  |  |
|  | Age interval | 1859.49 | 9 | 206.1 | 0.94 | .504 | .17 | [.00, .18] |
|  | Error | 9258.19 | 42 | 220.43 |  |  |  |  |
| Total distress | Mean minutes | 87552.16 | 1 | 87552.76 | 210.76 | .000 |  |  |
|  | Age interval | 8357.18 | 7 | 1193.88 | 2.87 | .030 | .50 | [.04, .55] |
|  | Error | 8308.37 | 20 | 415.42 |  |  |  |  |

S. Table 2. Results of the Tukey HSD tests comparing the different age intervals

| Age interval comparison | diff | 95% CI, LL | 95% CI, UL | adjusted p |
| --- | --- | --- | --- | --- |
| 38+-18-22 | 5.47 | -85.61 | 96.55 | 1.000 |
| 33-37-18-22 | 21.85 | -115.85 | 159.55 | 1.000 |
| 11-12-18-22 | 23.07 | -54.61 | 100.74 | 0.996 |
| 23-27-18-22 | 23.12 | -56.39 | 102.62 | 0.997 |
| 13-17-18-22 | 31.83 | -44.04 | 107.70 | 0.950 |
| 9-10-18-22 | 46.88 | -44.20 | 137.96 | 0.835 |
| 7-8-18-22 | 54.36 | -21.51 | 130.23 | 0.403 |
| 3-4-18-22 | 54.82 | -22.86 | 132.49 | 0.426 |
| 1-2-18-22 | 57.57 | -19.41 | 134.55 | 0.339 |
| 5-6-18-22 | 72.88 | 0.67 | 145.10 | 0.046 |
| 33-37-38+ | 16.38 | -116.95 | 149.71 | 1.000 |
| 11-12-38+ | 17.60 | -52.03 | 87.23 | 0.999 |
| 23-27-38+ | 17.65 | -54.01 | 89.31 | 0.999 |
| 13-17-38+ | 26.36 | -41.25 | 93.97 | 0.970 |
| 9-10-38+ | 41.41 | -42.92 | 125.73 | 0.871 |
| 7-8-38+ | 48.90 | -18.71 | 116.51 | 0.389 |
| 3-4-38+ | 49.35 | -20.28 | 118.98 | 0.420 |
| 1-2-38+ | 52.10 | -16.75 | 120.95 | 0.322 |
| 5-6-38+ | 67.42 | 3.94 | 130.89 | 0.028 |
| 11-12-33-37 | 1.22 | -123.34 | 125.77 | 1.000 |
| 23-27-33-37 | 1.27 | -124.44 | 126.97 | 1.000 |
| 13-17-33-37 | 9.98 | -113.46 | 133.42 | 1.000 |
| 9-10-33-37 | 25.03 | -108.30 | 158.35 | 1.000 |
| 7-8-33-37 | 32.51 | -90.92 | 155.95 | 0.999 |
| 3-4-33-37 | 32.97 | -91.59 | 157.52 | 0.999 |
| 1-2-33-37 | 35.72 | -88.40 | 159.84 | 0.997 |
| 5-6-33-37 | 51.03 | -70.19 | 172.26 | 0.949 |
| 23-27-11-12 | 0.05 | -53.55 | 53.65 | 1.000 |
| 13-17-11-12 | 8.76 | -39.28 | 56.81 | 1.000 |
| 9-10-11-12 | 23.81 | -45.82 | 93.44 | 0.988 |
| 7-8-11-12 | 31.30 | -16.75 | 79.35 | 0.549 |
| 3-4-11-12 | 31.75 | -19.10 | 82.60 | 0.611 |
| 1-2-11-12 | 34.50 | -15.28 | 84.28 | 0.454 |
| 5-6-11-12 | 49.82 | 7.78 | 91.85 | 0.008 |
| 13-17-23-27 | 8.71 | -42.24 | 59.66 | 1.000 |
| 9-10-23-27 | 23.76 | -47.90 | 95.42 | 0.991 |
| 7-8-23-27 | 31.25 | -19.70 | 82.20 | 0.636 |
| 3-4-23-27 | 31.70 | -21.90 | 85.30 | 0.685 |
| 1-2-23-27 | 34.45 | -18.13 | 87.04 | 0.540 |
| 5-6-23-27 | 49.77 | 4.45 | 95.09 | 0.019 |
| 9-10-13-17 | 15.05 | -52.56 | 82.66 | 1.000 |
| 7-8-13-17 | 22.53 | -22.54 | 67.61 | 0.858 |
| 3-4-13-17 | 22.99 | -25.06 | 71.03 | 0.889 |
| 1-2-13-17 | 25.74 | -21.17 | 72.65 | 0.773 |
| 5-6-13-17 | 41.05 | 2.46 | 79.65 | 0.027 |

Using the 5-6 weeks category as the comparison age interval, we noted significant differences in cry/fuss duration (minutes) between:

- 5-6 and 9-11 weeks (diff = 49.82, 95% CI [7.78; 91.85], p < 0.01)
- 5-6 and 13-17 weeks (diff = 41.06, 95% CI [2.46; 79.65], p < 0.05)
- 5-6 and 18-22 weeks (diff = 72.89, 95% CI [0.67; 145.10], p < 0.05)
- 5-6 and 23-27 weeks (diff = 49.77, 95% CI [4.45; 95.09], p < 0.05)
- 5-6 and 38+ weeks (diff = 67.42, 95% CI [3.94; 130.89], p < 0.05)

# *Differences across Age intervals: Regression results*

We compared the mean cry durations (cry/fuss; crying only; “total distress”) for each age interval to the overall mean duration. The durations at age intervals 11-12 weeks (b = -34.50, SE = 15.12, t(102) = -2.28, p = 0.03), 18-22 (b = -57.57, SE = 23.38, t(102) = -2.46, p = 0.02), 23-27 (b = -34.45, SE = 15.97, t(102) = -2.16, p = 0.03) and 38+ weeks (b = -52.10, SE = 20.91, t(102) = -2.49, p = 0.01) were all significantly lower than the intercept (b values, -34.4- 58; p values = 0.01-.0 03). In addition, the age interval 13-17 weeks was on the borderline of being significantly different from the intercept (b = -25.74, SE = 14.25, t(102) = -1.81, p = 0.07).

S. Table 3. Regression models for the 3 crying outcomes, with age interval as a predictor

| Outcome Variable | Predictor | B | SE | | t | p | R2 | R2 adjusted |
| --- | --- | --- | --- | --- | --- | --- | --- | --- |
| Cry/fuss |  | | | | | | .28 | .20 |
|  | Intercept | 99.32 | 10.45 | | 9.50 | .000 *** |  |  |
|  | 3-4 weeks | -2.75 | 15.12 | | -0.18 | .856 |  |  |
|  | 5-6 weeks | 15.32 | 12.37 | | 1.24 | .219 |  |  |
|  | 7-8 weeks | -3.21 | 14.25 | | -0.23 | .823 |  |  |
|  | 9-10 weeks | -10.69 | 20.91 | | -0.51 | .610 |  |  |
|  | 11-12 weeks | -34.50 | 15.12 | | -2.28 | .025 * |  |  |
|  | 13-17 weeks | -25.74 | 14.25 | | -1.81 | .074 . |  |  |
|  | 18-22 weeks | -57.57 | 23.38 | | -2.46 | .016 * |  |  |
|  | 23-27 weeks | -34.45 | 15.97 | | -2.16 | .033 * |  |  |
|  | 33-37 weeks | -35.72 | 37.69 | | -0.95 | .346 |  |  |
|  | 38+ weeks | -52.10 | 20.91 | | -2.49 | .014 * |  |  |
| Crying only |  | | | | | | .17 | -.01 |
|  | Intercept | 43.01 | | 5.25 | 8.19 | .000 *** |  |  |
|  | 3-4 weeks | -9.16 | | 11.74 | -0.78 | .440 |  |  |
|  | 5-6 weeks | -8.01 | | 6.37 | -1.26 | .215 |  |  |
|  | 7-8 weeks | -3.23 | | 10.05 | -0.32 | .750 |  |  |
|  | 9-10 weeks | -9.01 | | 15.75 | -0.57 | .570 |  |  |
|  | 11-12 weeks | -9.14 | | 8.02 | -1.14 | .261 |  |  |
|  | 13-17 weeks | -10.05 | | 8.46 | -1.19 | .242 |  |  |
|  | 23-27 weeks | -21.21 | | 9.09 | -2.33 | .025 * |  |  |
|  | 33-37 weeks | -12.01 | | 15.75 | -0.76 | .450 |  |  |
|  | 38+ weeks | -18.84 | | 8.46 | -2.26 | .032 * |  |  |
| Total distress |  | | | | | | .50 | .33 |
|  | Intercept | 120.78 | 8.32 | | 14.52 | .000 *** |  |  |
|  | 3-4 weeks | 22.25 | 16.64 | | 1.34 | .196 |  |  |
|  | 5-6 weeks | 13.21 | 10.19 | | 1.30 | .210 |  |  |
|  | 7-8 weeks | -30.80 | 22.02 | | -1.40 | .177 |  |  |
|  | 9-10 weeks | -10.70 | 22.02 | | -0.49 | .632 |  |  |
|  | 11-12 weeks | -35.86 | 14.41 | | -2.49 | .022 * |  |  |
|  | 13-17 weeks | -6.40 | 22.02 | | -0.29 | .774 |  |  |
|  | 23-27 weeks | -12.82 | 16.64 | | -0.77 | .450 |  |  |

# *Subgroup Analyses for infant fuss/cry duration.*

We examined the models with Random Effects per age group using Forest plots.


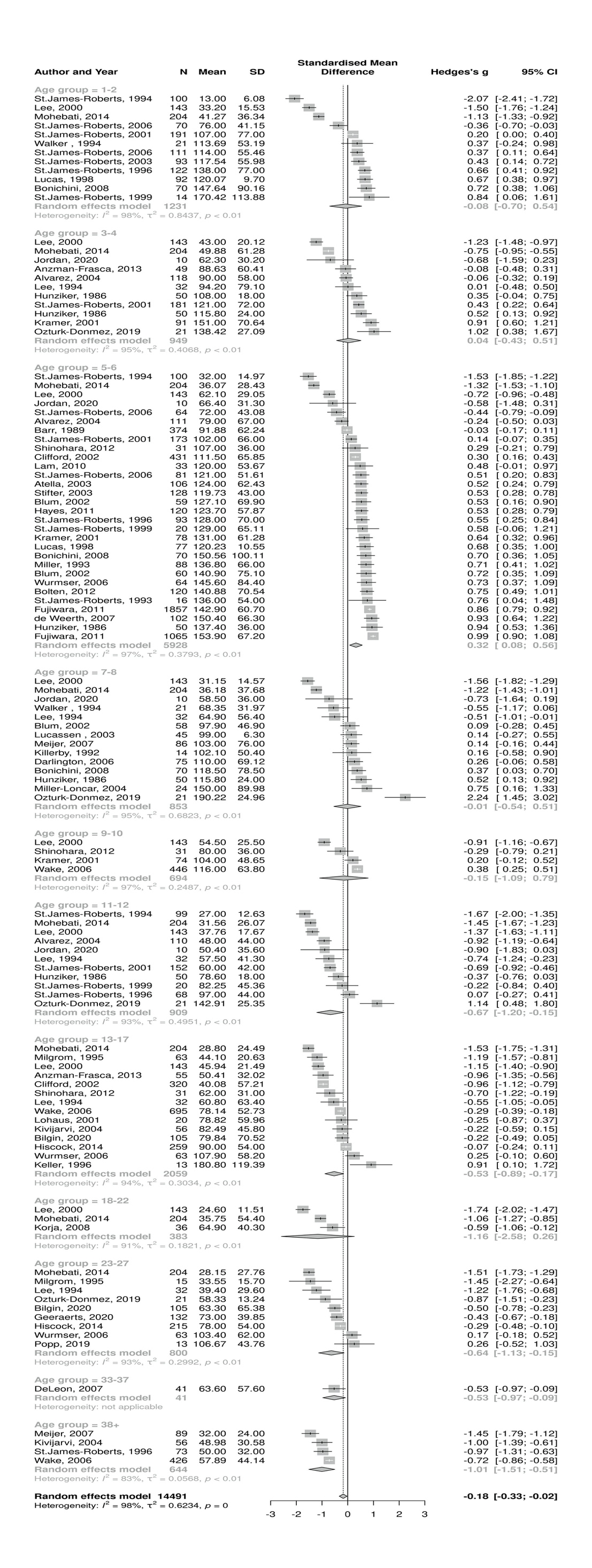


S. Figure 1a. Forest plots for the Random Effect model on age groups for cry/fuss durations.

We examined the models with Random Effects per country for each age interval independently using Forest plots.


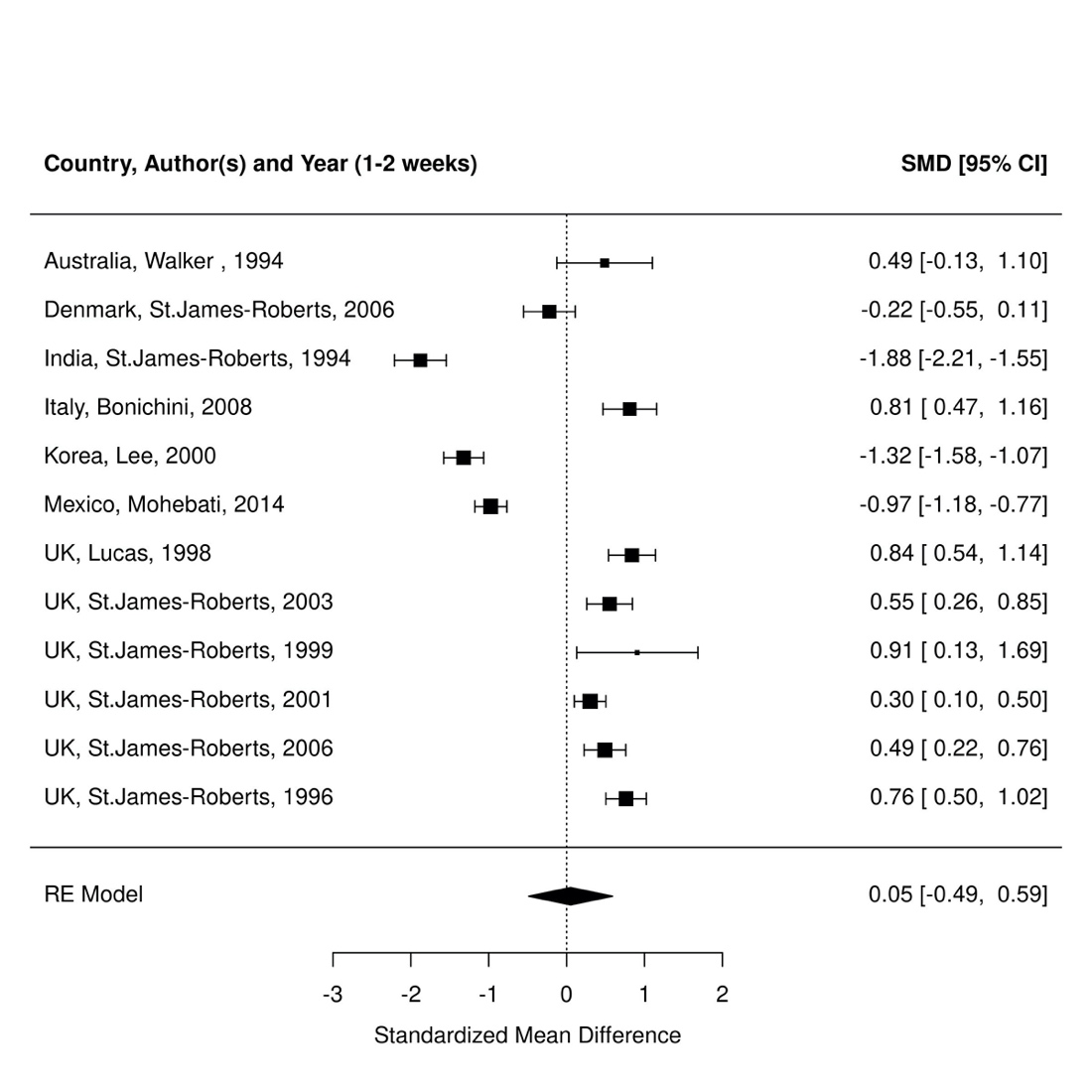

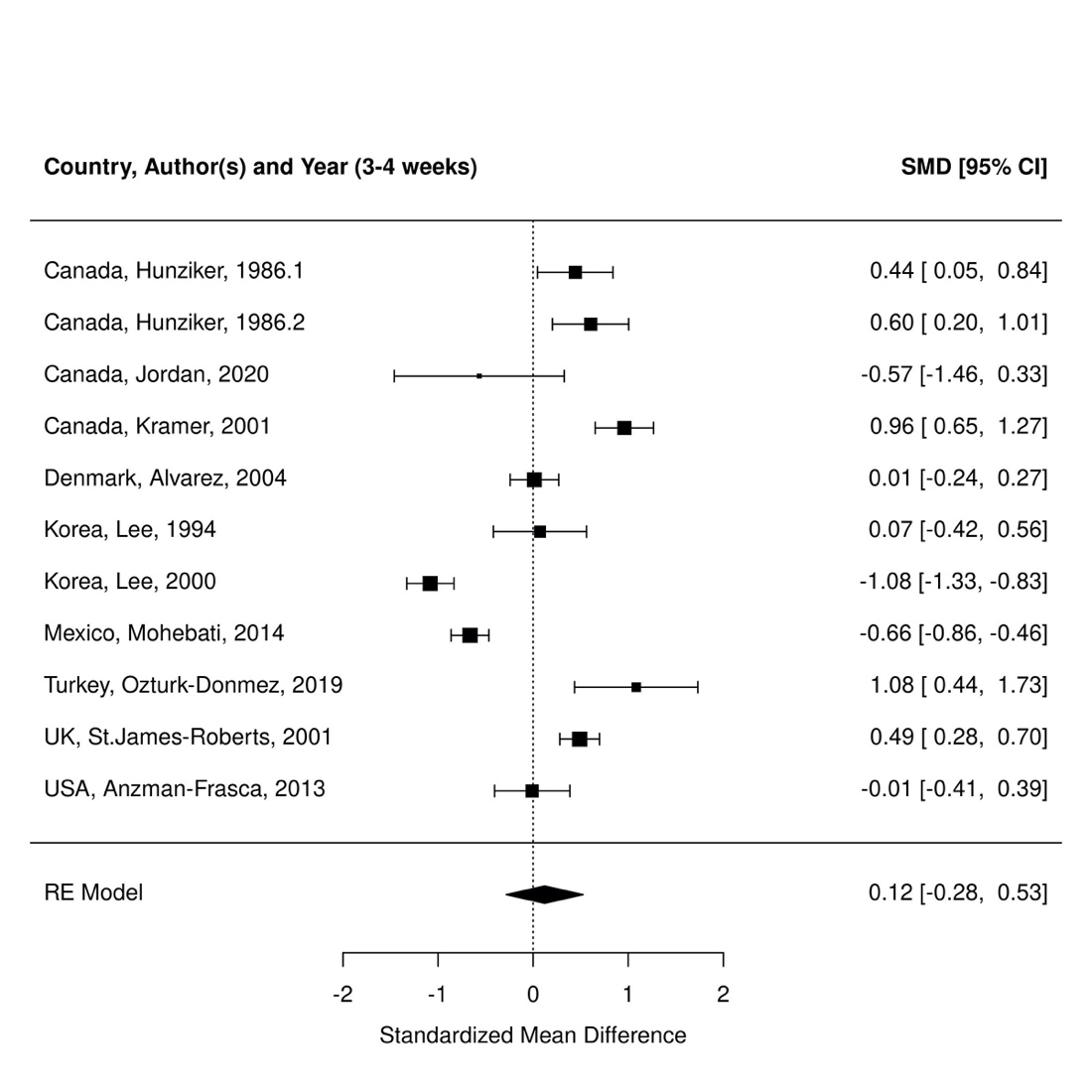

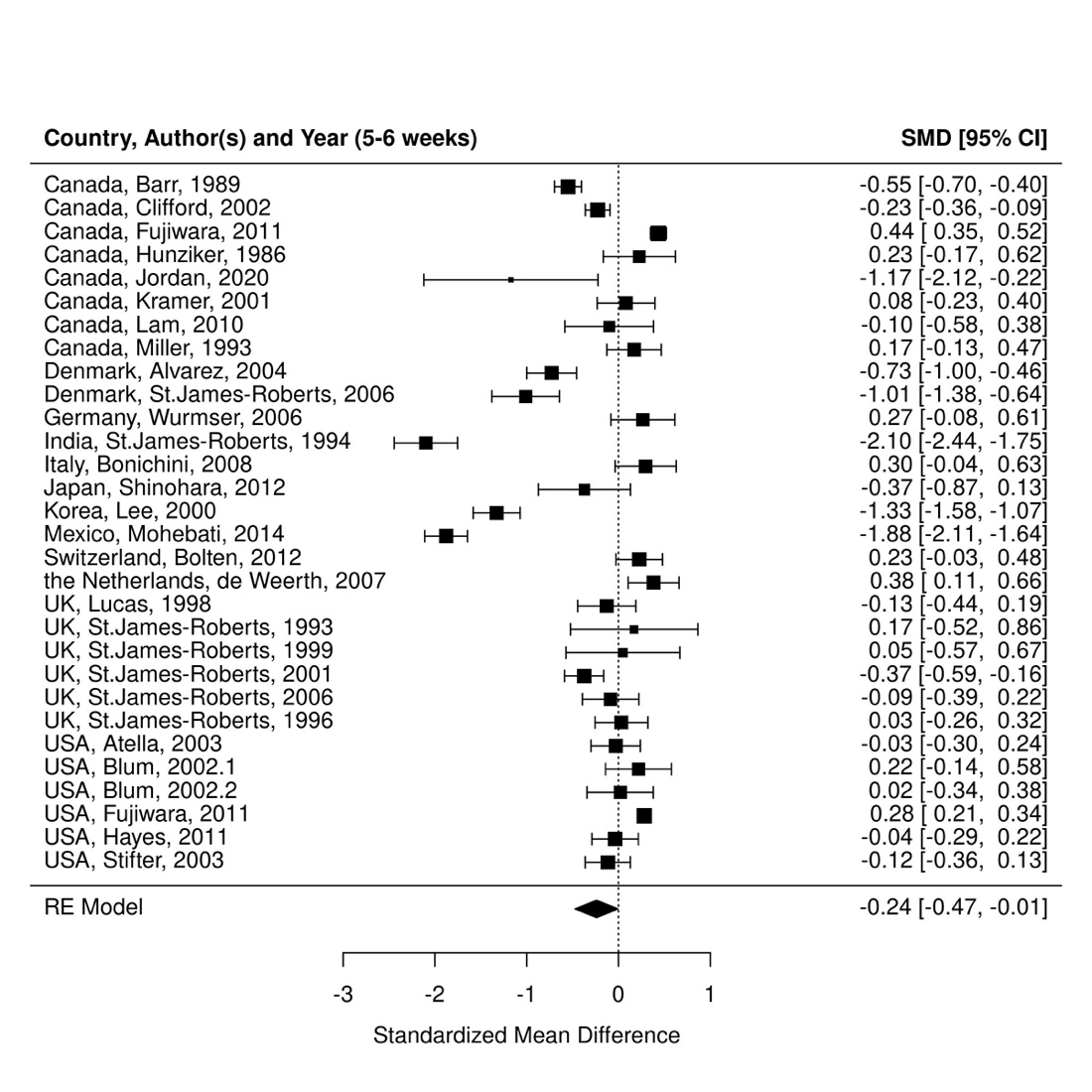

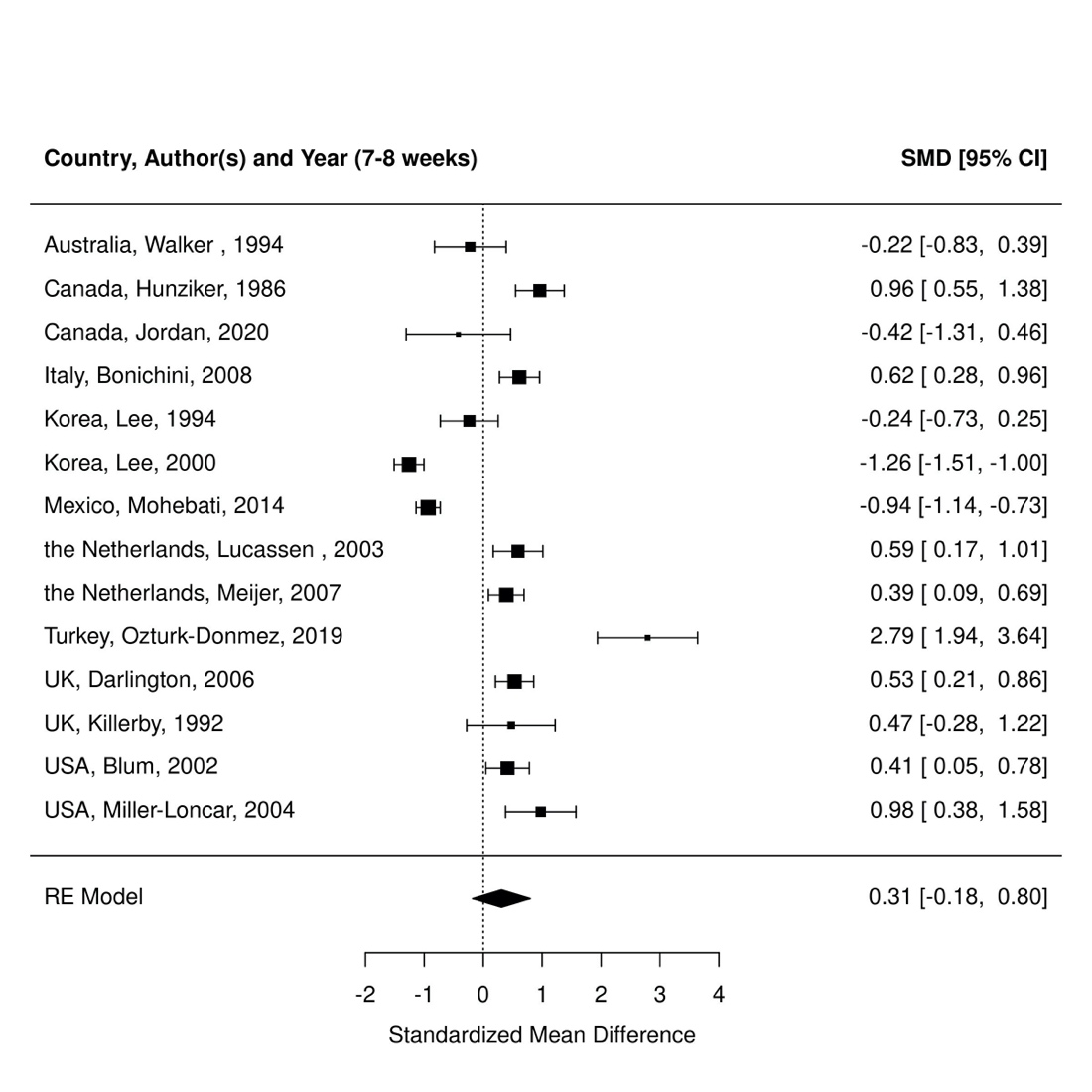

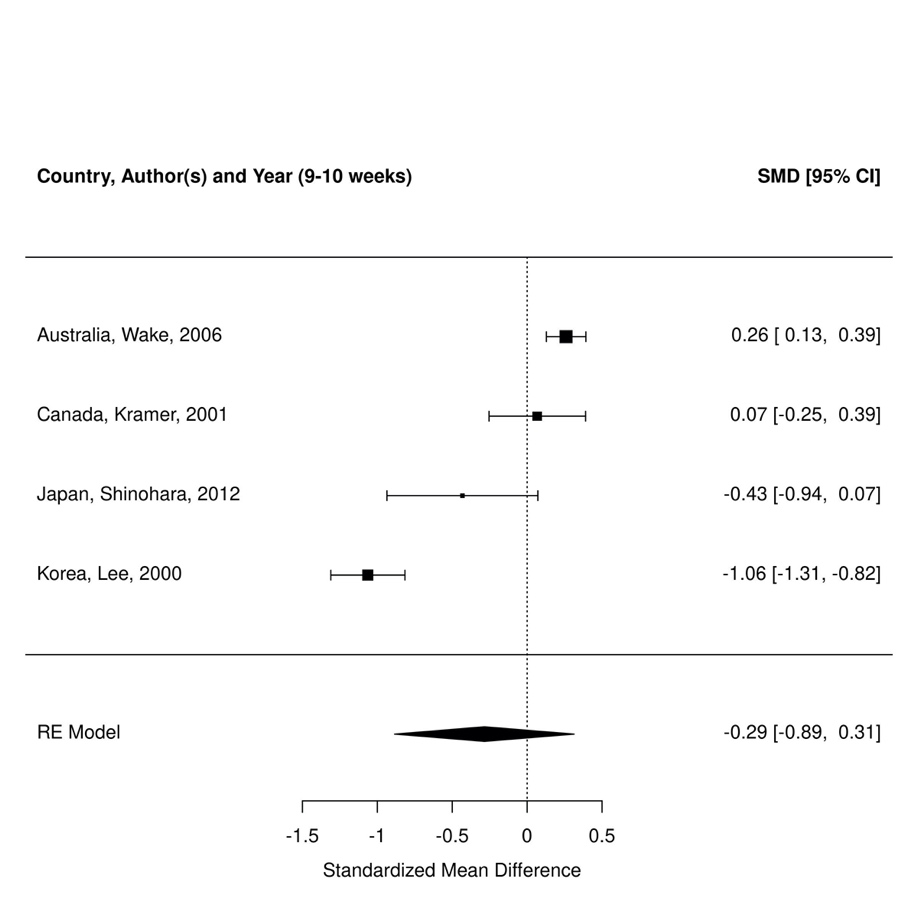

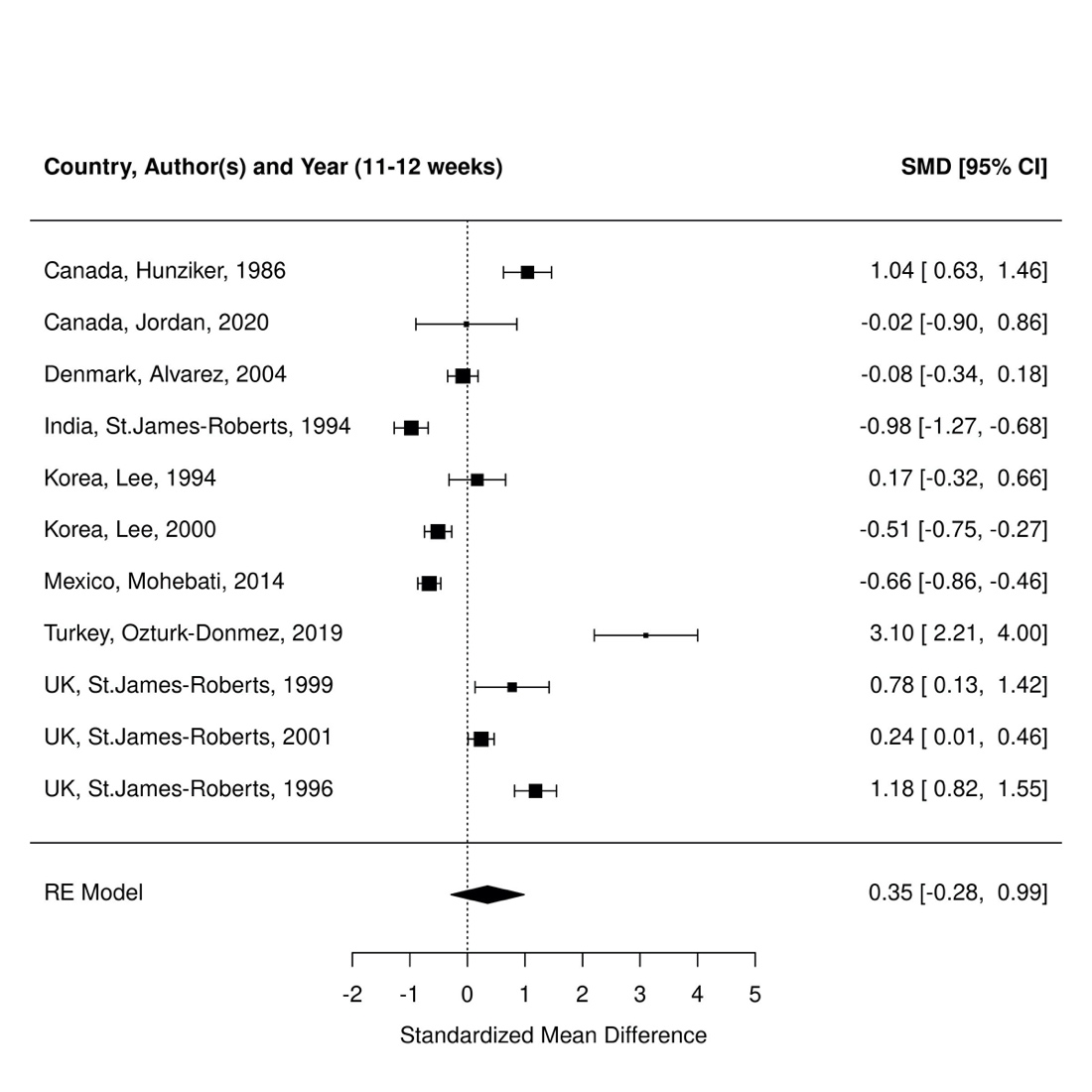

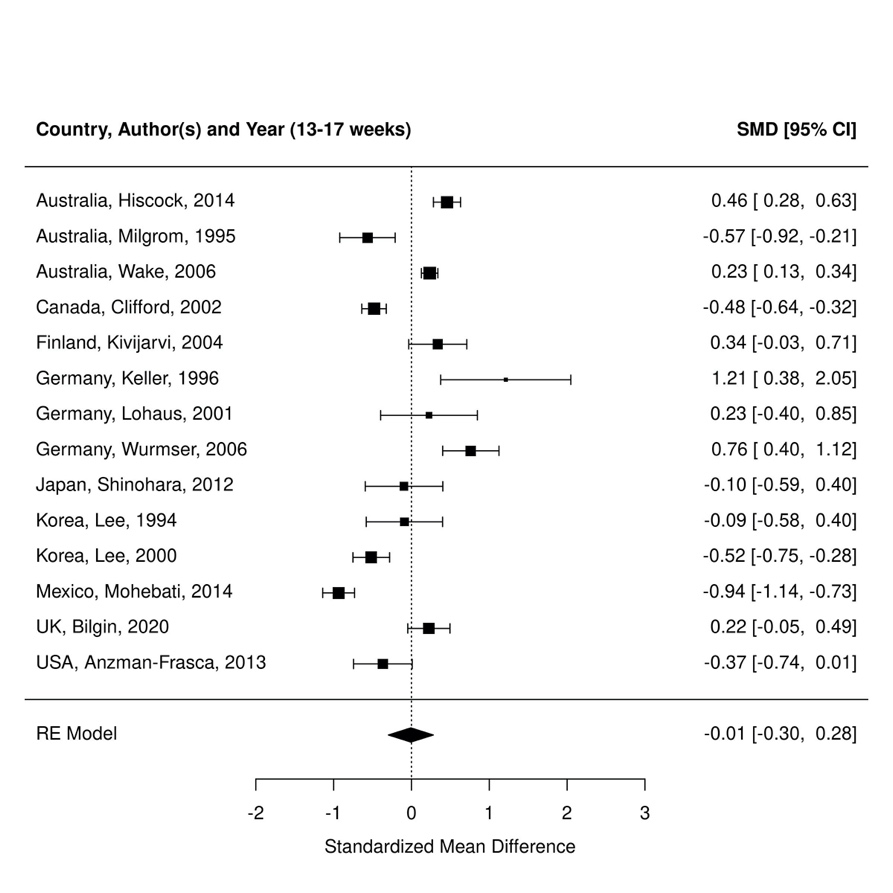

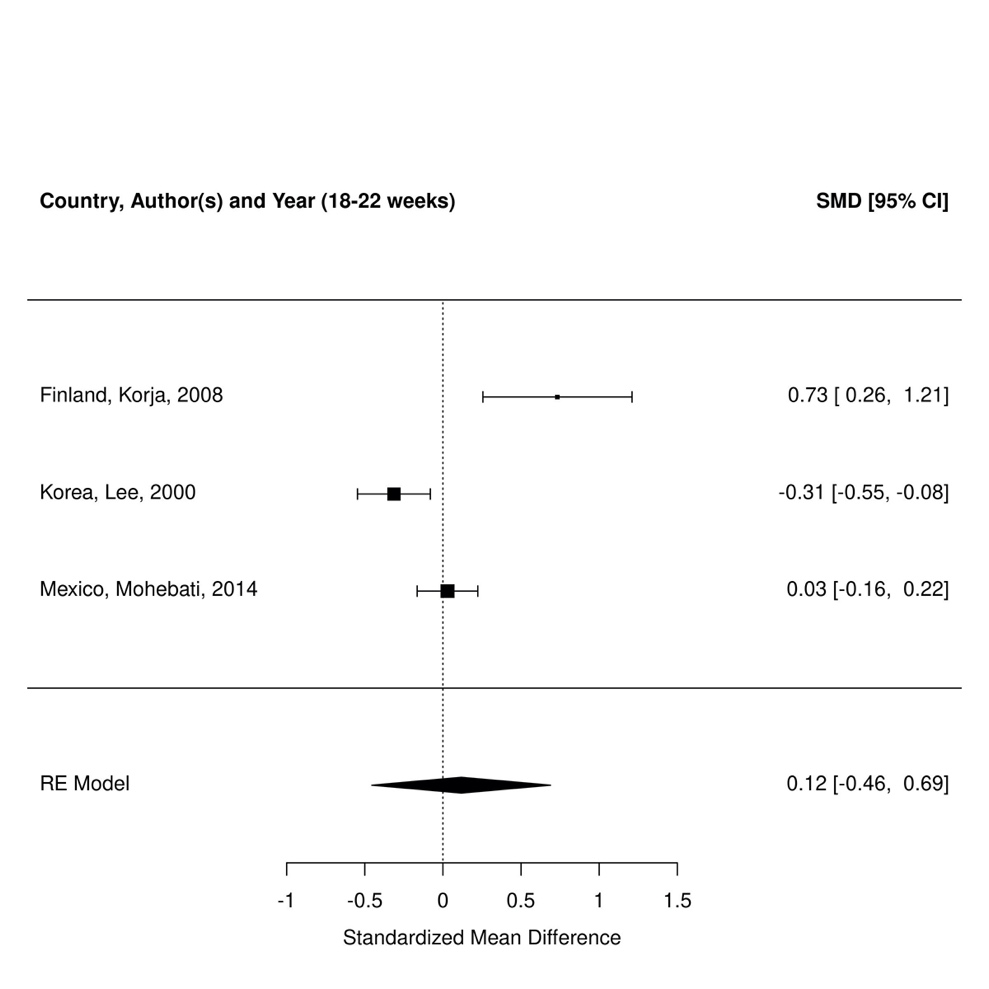

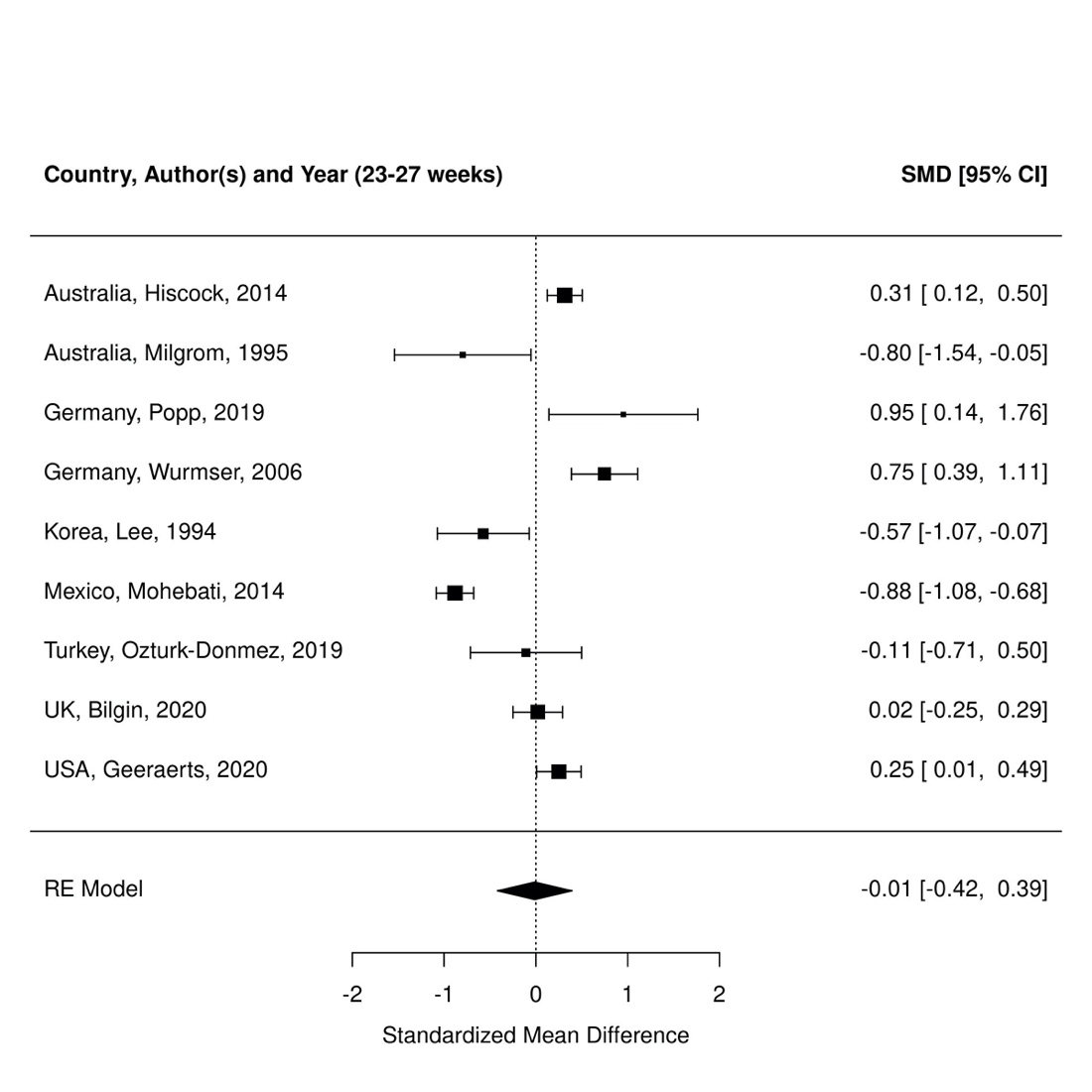

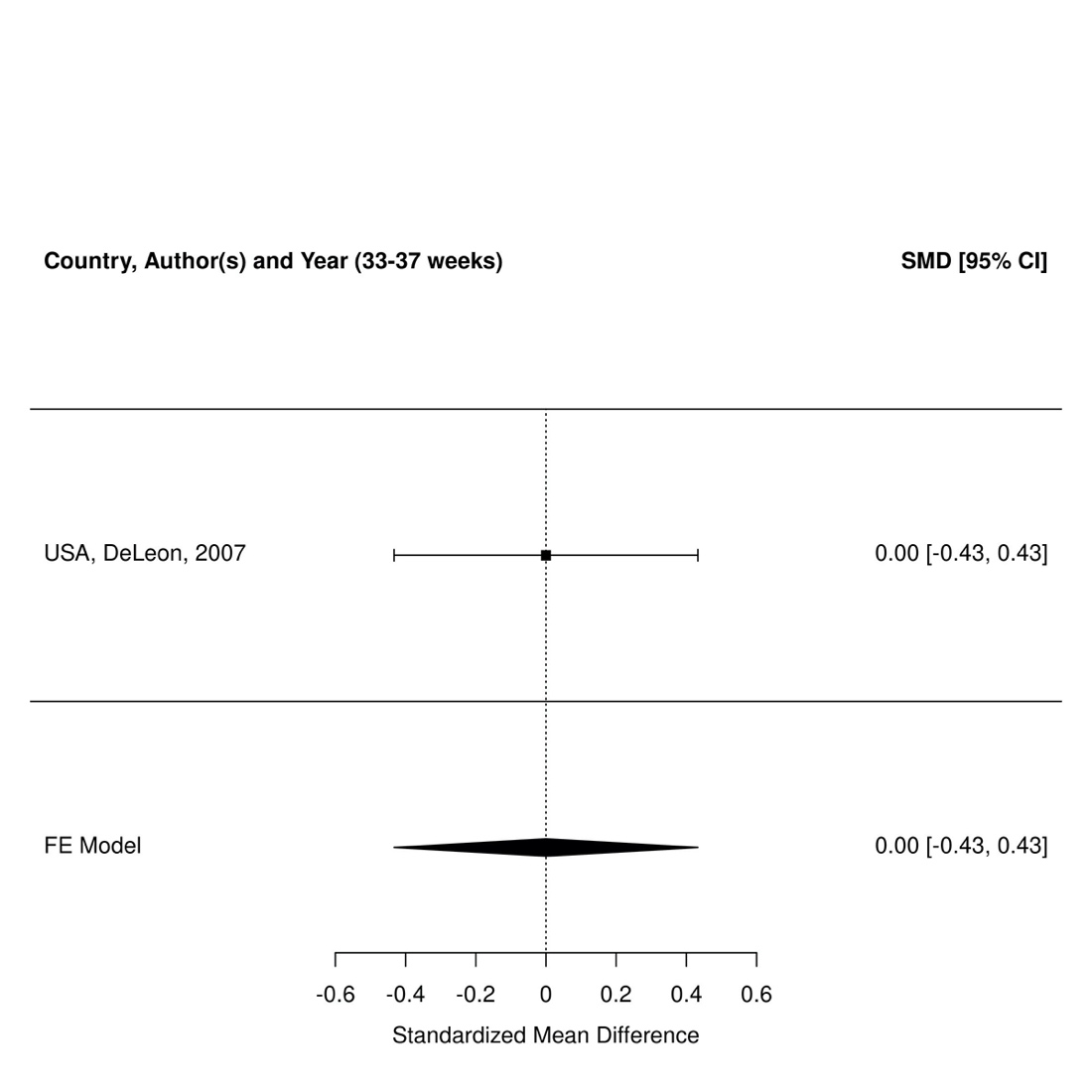

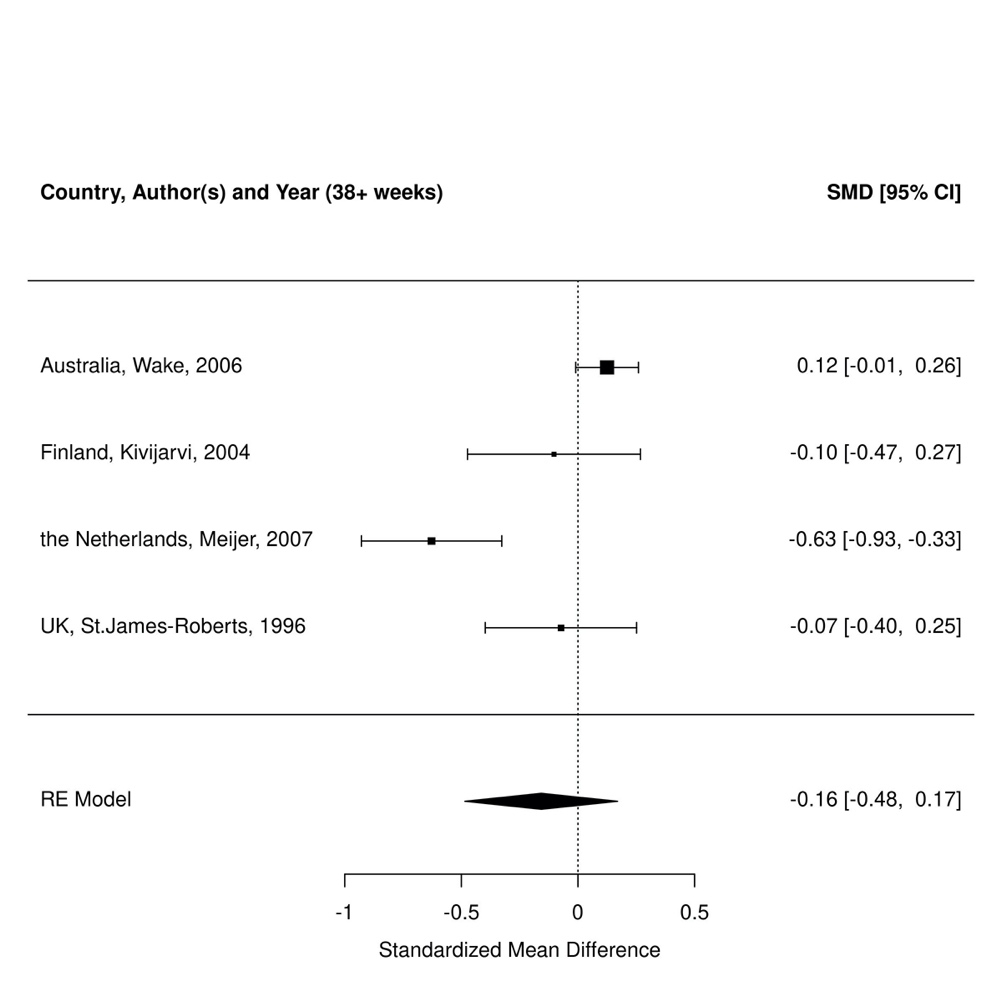


S. Figure 1b. Forest plots for the subgroup analyses for cry/fuss durations, divided into age intervals. Each study is labelled by country and then author and year.

# *Publication bias*

S. Table 4 provides the results of the Duval and Tweedie “trim and fill” method (Duval & Tweedie, 2000), which suggested a single missing study at age 1-2 weeks on the left side of the funnel plot.

S. Table 4. Results of the Trim and Fill test (Duval & Tweedie, 2000).

| **1-2 weeks** | **Estimated number of missing studies on the left side: 1 (SE = 2.3392)** | | | | |
| --- | --- | --- | --- | --- | --- |
|  |  |  |  |  |  |
|  | **Random-Effects Model (k = 13; tau^2 estimator: REML)** | | | | |
|  | tau^2 | 0.8609 (SE = 0.3695) | | **Model Results:** | |
|  | tau | 0.9279 |  |  |  |
|  | I^2 | 97.32% |  | estimate | -0.0172 |
|  | H^2 | 37.33 |  | se | 0.264 |
|  |  |  |  | z value | -0.0651 |
|  | **Test for Heterogeneity:** | |  | p value | 0.9481 |
|  | Q(df = 12) | 441.5538 |  | ci 2.5% | -0.5347 |
|  | p value | < .0001 |  | ci 97.5% | 0.5003 |
| **3-4 weeks** | **Estimated number of missing studies on the left side: 0 (SE = 2.1765)** | | | | |
|  |  |  |  |  |  |
|  | **Random-Effects Model (k = 11; tau^2 estimator: REML)** | | | | |
|  | tau^2 | 0.4218 (SE = 0.2091) | | **Model Results:** | |
|  | tau | 0.6495 |  |  |  |
|  | I^2 | 94.22% |  | estimate | 0.1208 |
|  | H^2 | 17.31 |  | se | 0.2065 |
|  |  |  |  | z value | 0.5851 |
|  | **Test for Heterogeneity:** | |  | p value | 0.5585 |
|  | Q(df = 10) | 199.5069 |  | ci 2.5% | -0.284 |
|  | p value | < .0001 |  | ci 97.5% | 0.5257 |
| **5-6 weeks** | **Estimated number of missing studies on the right side: 0 (SE = 2.5374)** | | | | |
|  |  |  |  |  |  |
|  | **Random-Effects Model (k = 30; tau^2 estimator: REML)** | | | | |
|  | tau^2 | 0.3920 (SE = 0.1113) |  | **Model Results:** | |
|  | tau | 0.6261 |  |  |  |
|  | I^2 | 97.04% |  | estimate | -0.2395 |
|  | H^2 | 33.78 |  | se | 0.119 |
|  |  |  |  | z value | -2.0115 |
|  | **Test for Heterogeneity:** | |  | p value | 0.0443 * |
|  | Q(df = 29) | 829.3416 |  | ci 2.5% | -0.4728 |
|  | p value | < .0001 |  | ci 97.5% | -0.0061 |
| **7-8 weeks** | **Estimated number of missing studies on the left side: 0 (SE = 2.3980)** | | | | |
|  |  |  |  |  |  |
|  | **Random-Effects Model (k = 14; tau^2 estimator: REML)** | | | | |
|  | tau^2 | 0.8123 (SE = 0.3452) |  | **Model Results:** | |
|  | tau | 0.9013 |  |  |  |
|  | I^2 | 95.53% |  | estimate | 0.3093 |
|  | H^2 | 22.39 |  | se | 0.2509 |
|  |  |  |  | z value | 1.2324 |
|  | **Test for Heterogeneity:** | |  | p value | 0.2178 |
|  | Q(df = 13) | 289.1384 |  | ci 2.5% | -0.1826 |
|  | p value | < .0001 |  | ci 97.5% | 0.8011 |
| **9-10 weeks** | **Estimated number of missing studies on the right side: 0 (SE = 1.5649)** | | | | |
|  |  |  |  |  |  |
|  | **Random-Effects Model (k = 4; tau^2 estimator: REML)** | | | | |
|  | tau^2 | 0.3474 (SE = 0.3057) |  | **Model Results:** | |
|  | tau | 0.5894 |  |  |  |
|  | I^2 | 95.27% |  | estimate | -0.2855 |
|  | H^2 | 21.13 |  | se | 0.3059 |
|  |  |  |  | z value | -0.9333 |
|  | **Test for Heterogeneity:** | |  | p value | 0.3507 |
|  | Q(df = 3) | 88.283 |  | ci 2.5% | -0.8852 |
|  | p value | < .0001 |  | ci 97.5% | 0.3141 |
| **11-12 weeks** | **Estimated number of missing studies on the left side: 0 (SE = 1.8861)** | | | | |
|  |  |  |  |  |  |
|  | Random-Effects Model (k = 11; tau^2 estimator: REML) |  |  |  |  |
|  | tau^2 | 1.0918 (SE = 0.5159) |  | **Model Results:** | |
|  | tau | 1.0449 |  |  |  |
|  | I^2 | 97.55% |  | estimate | 0.3503 |
|  | H^2 | 40.78 |  | se | 0.324 |
|  |  |  |  | z value | 1.0811 |
|  | **Test for Heterogeneity:** | |  | p value | 0.2797 |
|  | Q(df = 10) | 218.6812 |  | ci 2.5% | -0.2848 |
|  | p value | < .0001 |  | ci 97.5% | 0.9854 |

| **13-17 weeks** | **Estimated number of missing studies on the left side: 0 (SE = 2.3461)** | | | | |
| --- | --- | --- | --- | --- | --- |
|  |  |  |  |  |  |
|  | **Random-Effects Model (k = 14; tau^2 estimator: REML)** | | | | |
|  | tau^2 | 0.2654 (SE = 0.1184) | | **Model Results:** | |
|  | tau | 0.5152 |  |  |  |
|  | I^2 | 94.38% |  | estimate | -0.0078 |
|  | H^2 | 17.79 |  | se | 0.1472 |
|  |  |  |  | z value | -0.053 |
|  | **Test for Heterogeneity:** | |  | p value | 0.9577 |
|  | Q(df = 13) | 223.854 |  | ci 2.5% | -0.2964 |
|  | p value | < .0001 |  | ci 97.5% | 0.2808 |
| **18-22 weeks** | **Estimated number of missing studies on the left side: 0 (SE = 1.4967)** | | | | |
|  |  |  |  |  |  |
|  | **Random-Effects Model (k = 3; tau^2 estimator: REML)** | | | | |
|  | tau^2 | 0.2297 (SE = 0.2560) |  | **Model Results:** | |
|  | tau | 0.4793 |  |  |  |
|  | I^2 | 92.46% |  | estimate | 0.1169 |
|  | H^2 | 13.26 |  | se | 0.2919 |
|  |  |  |  | z value | 0.4005 |
|  | **Test for Heterogeneity:** | |  | p value | 0.6888 |
|  | Q(df = 2) | 15.8578 |  | ci 2.5% | -0.4552 |
|  | p value | 0.0004 |  | ci 97.5% | 0.6891 |
| **23-27 weeks** | **Estimated number of missing studies on the left side: 0 (SE = 2.0082)** | | | | |
|  |  |  |  |  |  |
|  | **Random-Effects Model (k = 9; tau^2 estimator: REML)** | | | | |
|  | tau^2 | 0.3331 (SE = 0.1930) |  | **Model Results:** | |
|  | tau | 0.5771 |  |  |  |
|  | I^2 | 92.85% |  | estimate | -0.0132 |
|  | H^2 | 13.98 |  | se | 0.2077 |
|  |  |  |  | z value | -0.0634 |
|  | **Test for Heterogeneity:** | |  | p value | 0.9494 |
|  | Q(df = 8) | 117.2605 |  | ci 2.5% | -0.4202 |
|  | p value | < .0001 |  | ci 97.5% | 0.3938 |
| **28-32 weeks** | **NO DATA** | | | | |
|  |  |  |  |  |  |
|  |  |  |  |  |  |
|  |  |  |  |  |  |
|  |  |  |  |  |  |
|  |  |  |  |  |  |
|  |  |  |  |  |  |
|  |  |  |  |  |  |
|  |  |  |  |  |  |
|  |  |  |  |  |  |
|  |  |  |  |  |  |
| **33-37 weeks** | **ONLY 1 DATAPOINT** | | | | |
|  |  |  |  |  |  |
|  |  |  |  |  |  |
|  |  |  |  |  |  |
|  |  |  |  |  |  |
|  |  |  |  |  |  |
|  |  |  |  |  |  |
|  |  |  |  |  |  |
|  |  |  |  |  |  |
|  |  |  |  |  |  |
|  |  |  |  |  |  |
| **38+ weeks** | **Estimated number of missing studies on the right side: 0 (SE = 1.3802)** | | | | |
|  |  |  |  |  |  |
|  | **Random-Effects Model (k = 4; tau^2 estimator: REML)** | | | | |
|  | tau^2 | 0.0899 (SE = 0.0911) |  | **Model Results:** | |
|  | tau | 0.2998 |  |  |  |
|  | I^2 | 83.24% |  | estimate | -0.1575 |
|  | H^2 | 5.96 |  | se | 0.167 |
|  |  |  |  | z value | -0.9433 |
|  | **Test for Heterogeneity:** | |  | p value | 0.3455 |
|  | Q(df = 3) | 20.4183 |  | ci 2.5% | -0.4848 |
|  | p value | < .0001 |  | ci 97.5% | 0.1697 |

We examined publication bias using Funnel plots for each age interval, as follows:

1. 1-2 weeks


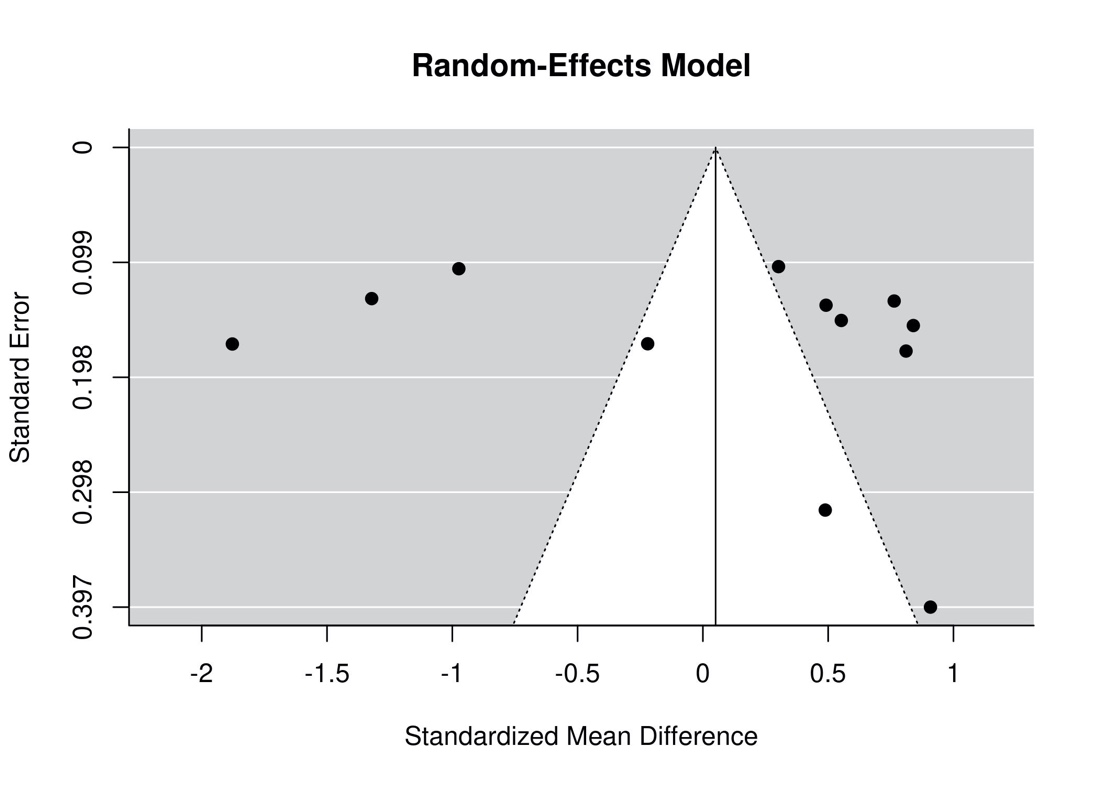


1. 3-4 weeks


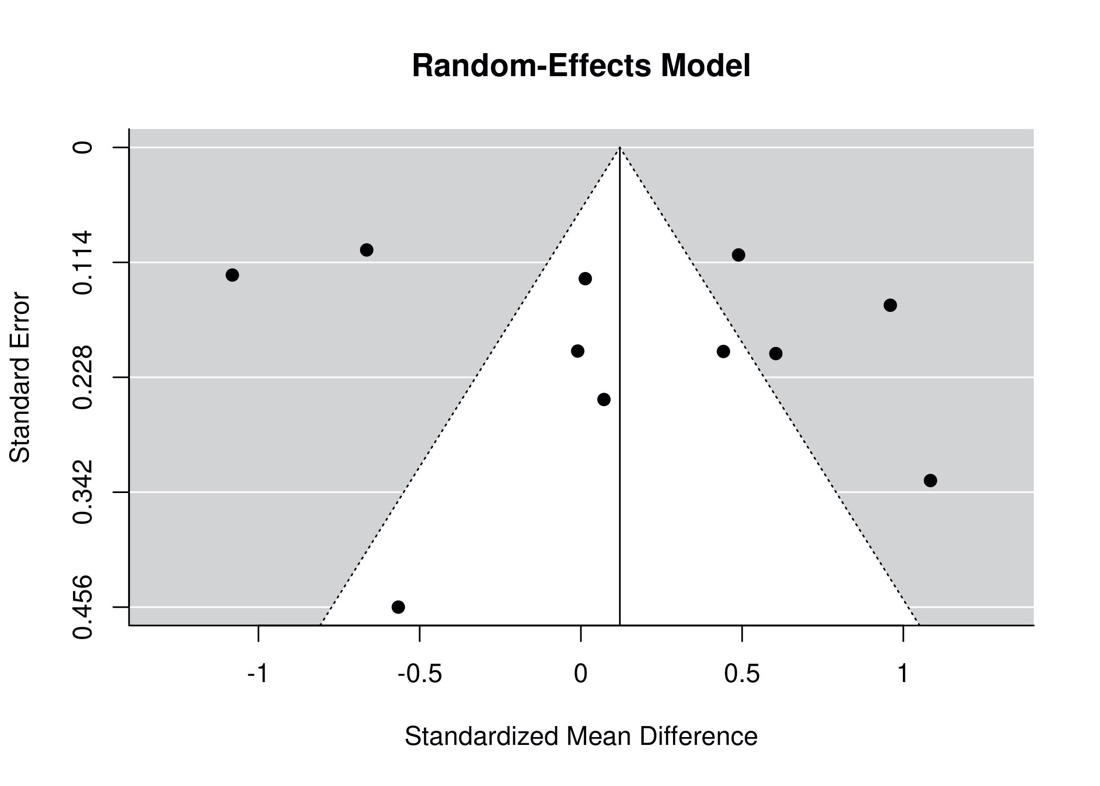


1. 5-6 weeks


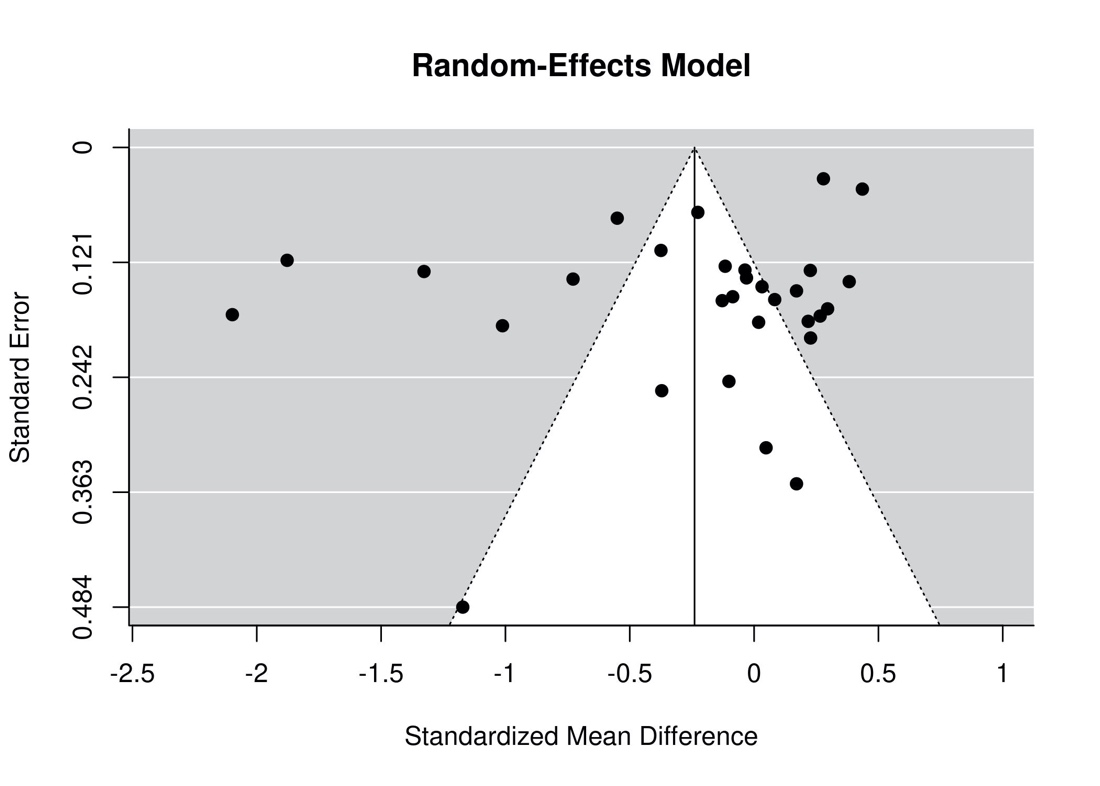


1. 7-8 weeks


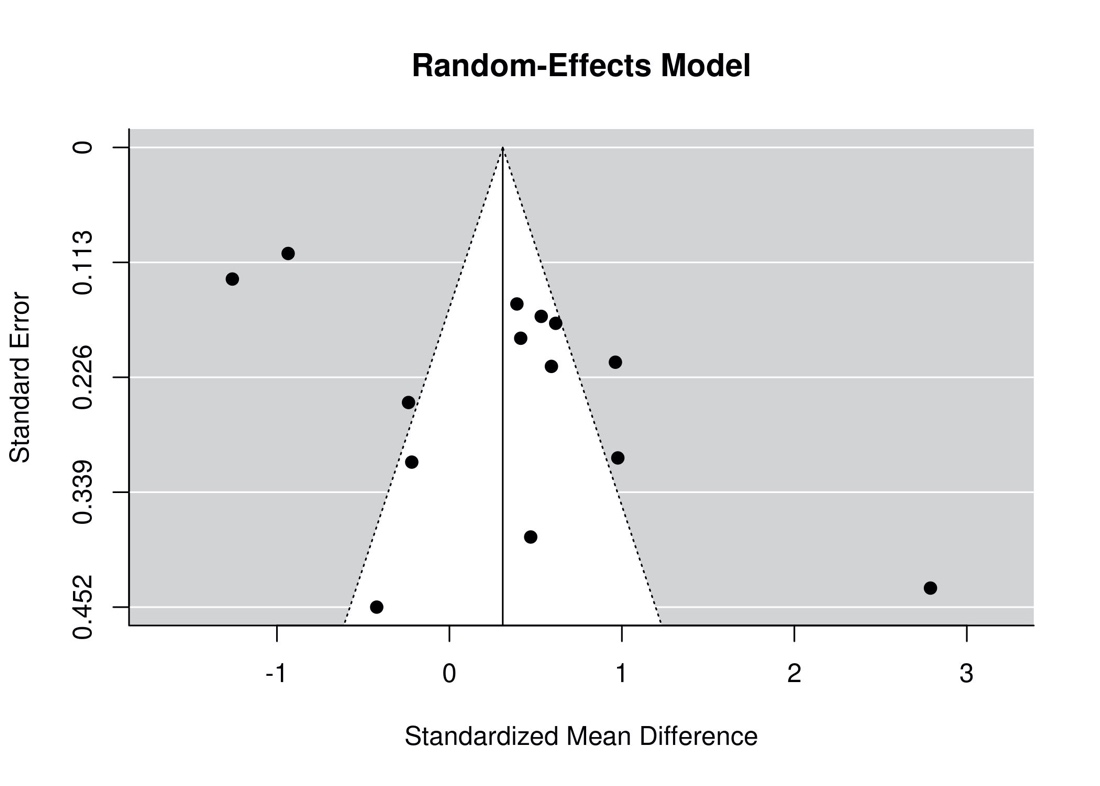


1. 9-10 weeks


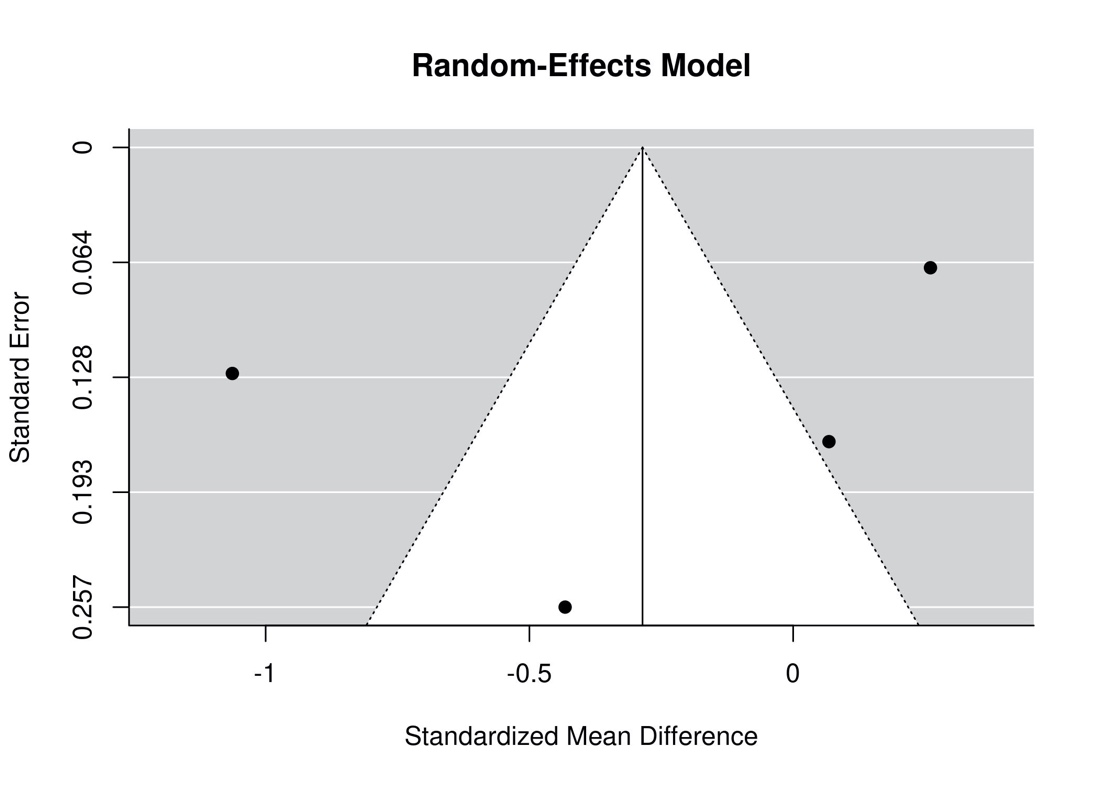


1. 11-12 weeks


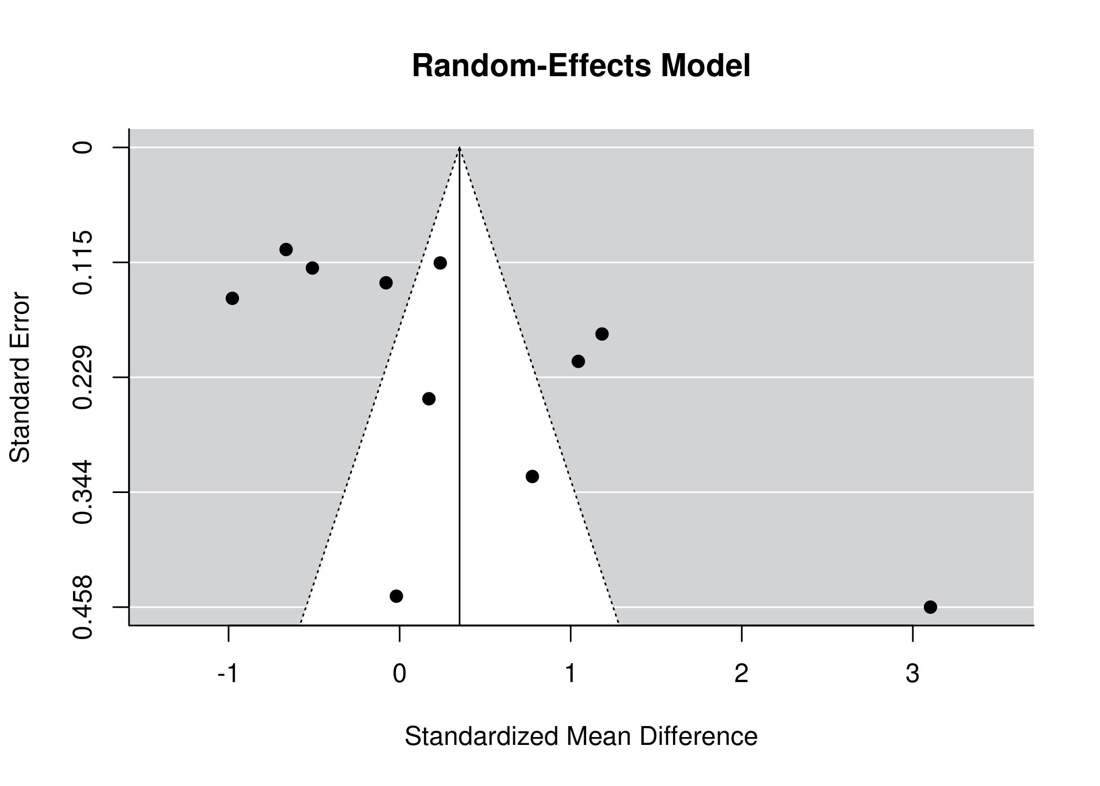


1. 13-17 weeks


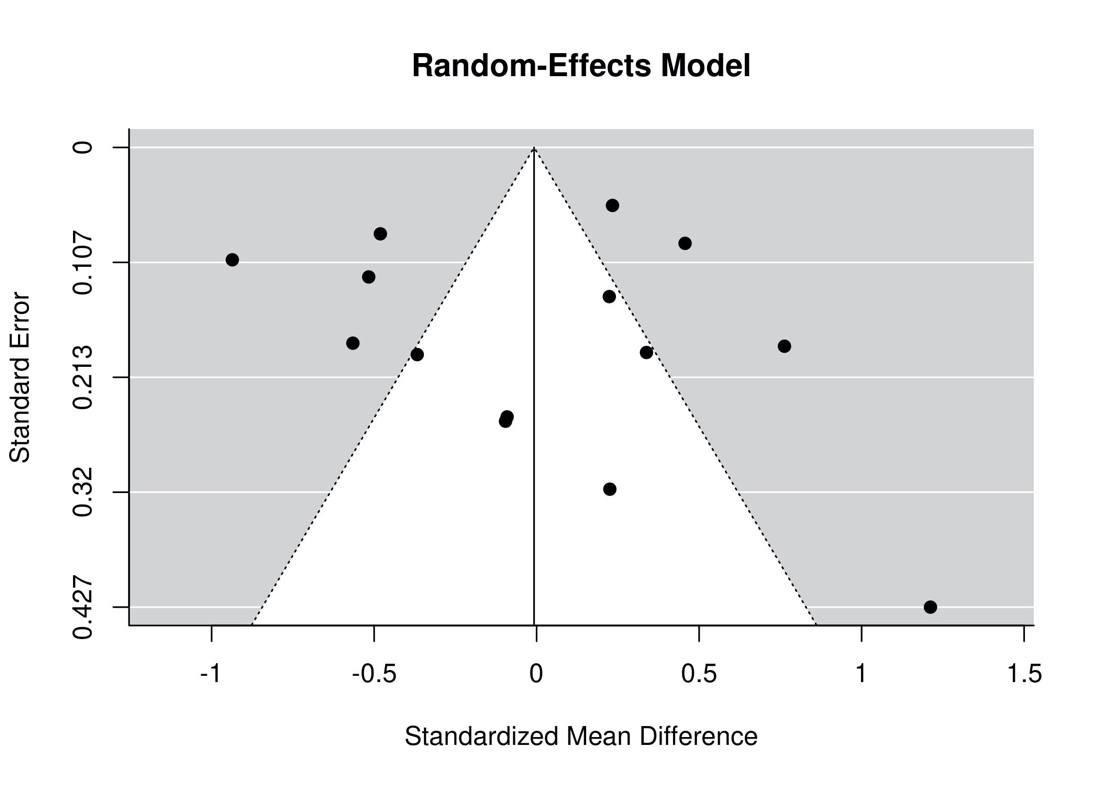


1. 18-22 weeks


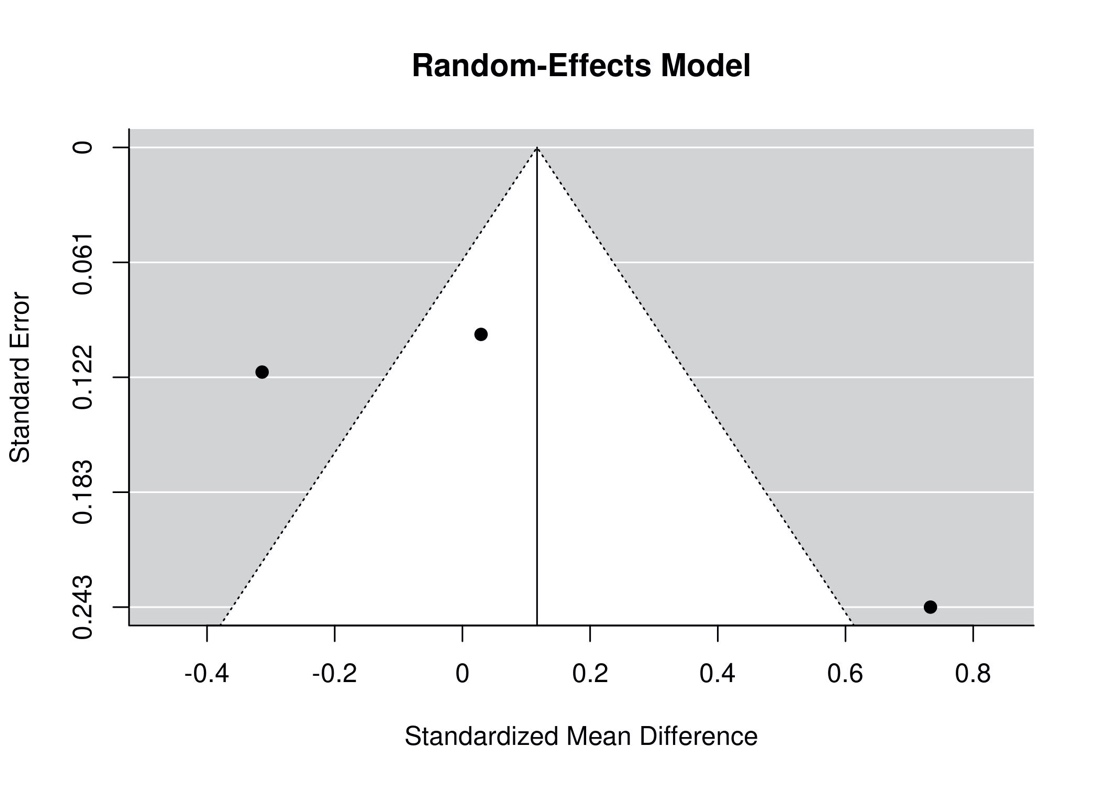


(i) 23-27 weeks


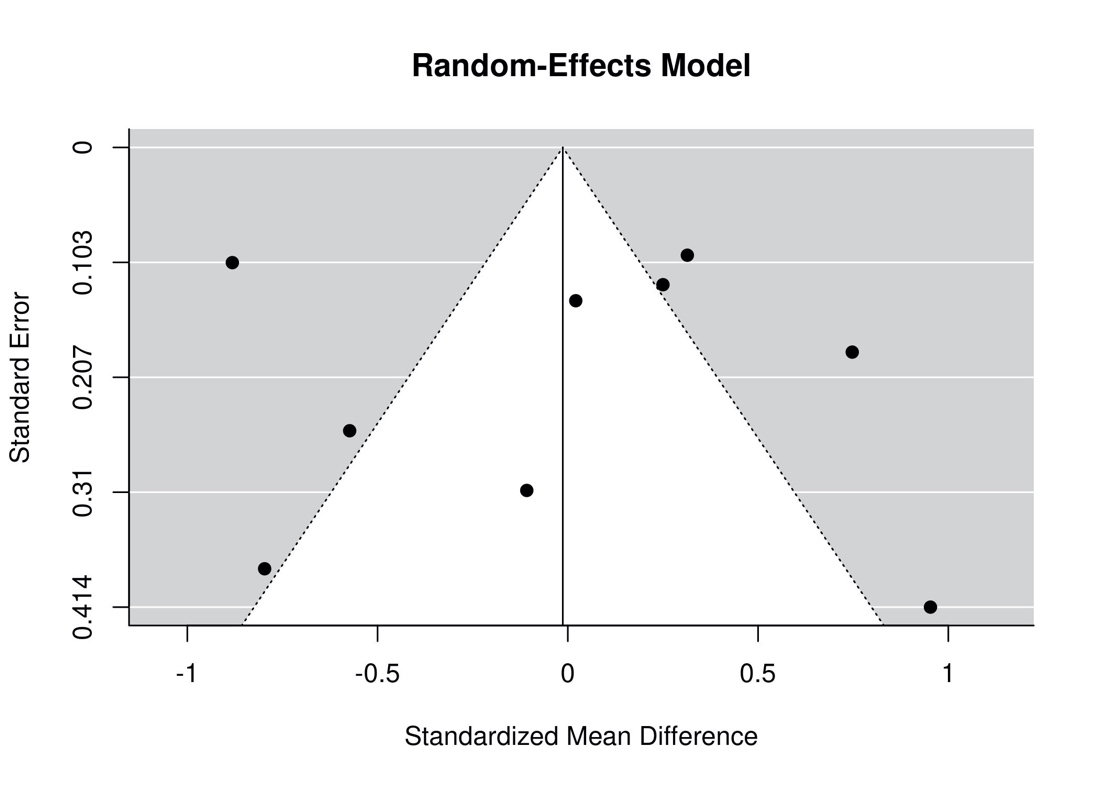


(j) 38+ weeks


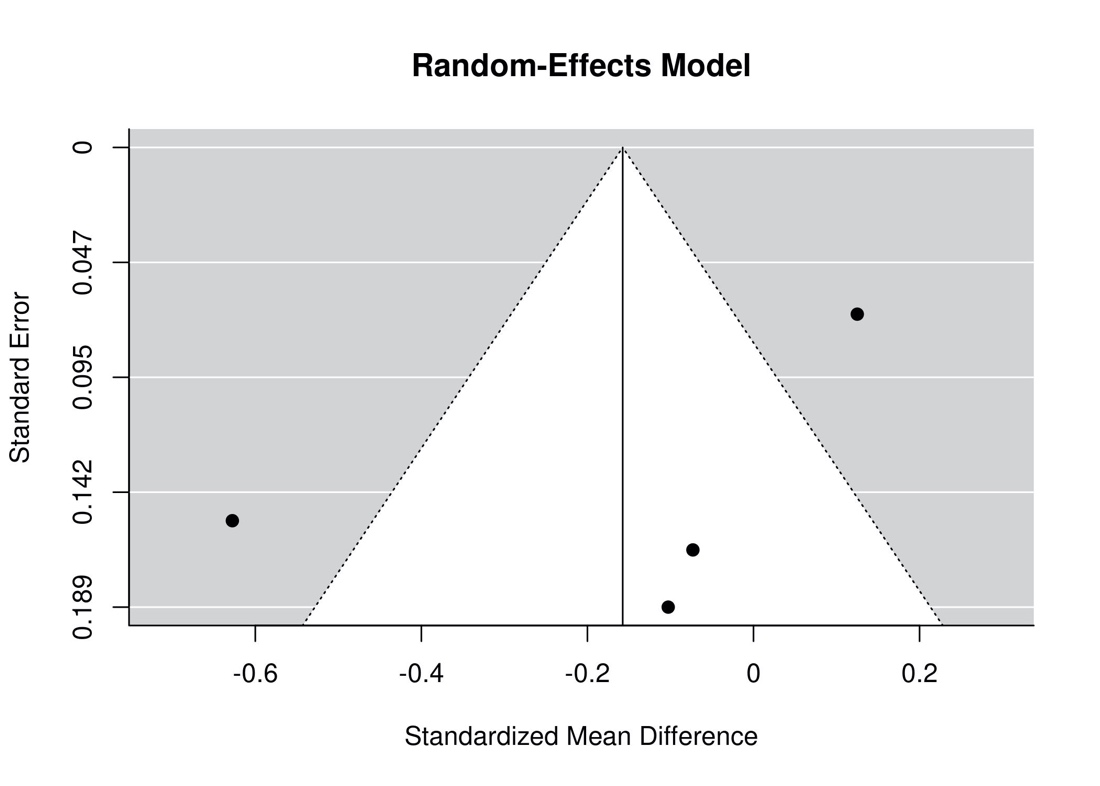


Figure S2. Funnel plots for each age interval (a-j)

# *Moderator analyses*

We carried two moderator analyses using metaregression to examine the associations between rated study quality and cry/fuss duration outcome, and between infant feeding type and cry/fuss duration outcomes.

S.Table 5. Quality rating for each study

| **Author** | **Year** | **Study Quality Rating** |
| --- | --- | --- |
| Aloisio | 2018 | 6 |
| Alvarez | 2004 | 7 |
| Anzman-Frasca | 2013 | 3 |
| Atella | 2003 | 5 |
| Balidam | 1995 | 6 |
| Barr | 1989 | 7 |
| Bilgin | 2020 | 5 |
| Blum | 2002 | 4 |
| Bolten | 2012 | 3 |
| Bonichini | 2008 | 4 |
| Clifford | 2002a | 6 |
| Clifford | 2002b | 5 |
| Darlington | 2006 | 2 |
| de Weerth | 2007 | 5 |
| DeLeon | 2007 | 4 |
| Fujiwara | 2011 | 6 |
| Geeraerts | 2020 | 5 |
| Harrison | 2004 | 3 |
| Hayes | 2011 | 4 |
| Hechler | 2018 | 3 |
| Hiscock | 2014 | 6 |
| Hunziker | 1986 | 6 |
| Jordan | 2020 | 3 |
| Keller | 1996 | 2 |
| Keller (D) | 1998 | 3 |
| Killerby | 2006 | 2 |
| Kivijärvi | 2004 | 2 |
| Korja | 2008 | 3 |
| Kramer | 2001 | 4 |
| Lam | 2010 | 5 |
| Lee | 1994 | 2 |
| Lee | 2000 | 3 |
| Litmanovitz | 2014 | 3 |
| Lohaus | 2001 | 3 |
| Lucas | 1998 | 4 |
| Lucassen | 2003 | 2 |
| McGlaughlin | 1999 | 1 |
| McRury | 2010 | 3 |
| Meijer | 2007 | 3 |
| Milgrom | 1995 | 5 |
| Miller | 1993 | 4 |
| Miller-Loncar | 2004 | 4 |
| Mohebati | 2014 | 4 |
| Öztürk Dönmez | 2019 | 4 |
| Popp | 2019 | 2 |
| Shinohara | 2012 | 6 |
| St. James (Cohort 2) | 2005 | 5 |
| St. James Roberts | 2001 | 5 |
| St. James Roberts | 1996 | 4 |
| St. James Roberts | 1999 | 3 |
| St. James Roberts | 1993 | 3 |
| St. James Roberts | 2003 | 3 |
| St. James Roberts | 2006 | 6 |
| St. James Roberts - Mali sample | 1994 | 4 |
| Stifter | 2003 | 6 |
| Wake | 2006 | 5 |
| Walker | 1994 | 3 |
| Wurmser | 2006 | 5 |

S. Table 6. Metaregression results with quality ratings as a continuous predictor for the 6 age intervals, where at least 10 participant samples were available.

| Age interval | Predictor | estimate | se | p | tau2 | I2 | H2 | R2 |
| --- | --- | --- | --- | --- | --- | --- | --- | --- |
| 1-2 weeks | intercept | -0.06 | 1.17 | 0.958 | 0.79 | 97.60% | 36.46 | 0.002 |
|  | Quality Rating | 0.03 | 0.28 | 0.923 |  |  |  |  |
| 3-4 weeks | intercept | -0.54 | 0.63 | 0.41 | 0.33 | 92.53% | 13.4 | 0.13 |
|  | Quality Rating | 0.15 | 0.14 | 0.294 |  |  |  |  |
| 5-6 weeks | intercept | -0.38 | 0.49 | 0.441 | 0.38 | 96.84% | 31.67 | 0.003 |
|  | Quality Rating | 0.03 | 0.1 | 0.764 |  |  |  |  |
| 7-8 weeks | intercept | -0.42 | 0.8 | 0.607 | 0.68 | 94.64% | 18.67 | 0.07 |
|  | Quality Rating | 0.22 | 0.23 | 0.358 |  |  |  |  |
| 11-12 weeks | intercept | 0.12 | 1.09 | 0.911 | 0.96 | 97.09% | 34.42 | 0.007 |
|  | Quality Rating | 0.05 | 0.25 | 0.836 |  |  |  |  |
| 13-17 weeks | intercept | 0.16 | 0.48 | 0.742 | 0.24 | 93.83% | 16.2 | 0 |
|  | Quality Rating | -0.04 | 0.11 | 0.71 |  |  |  |  |

S. Table 7. Metaregression results with feeding type (5 categories) as a predictor variable for the 2 age intervals with sufficient numbers of participant samples (k > 10)

| Results for subgroups (random effects model): | | | | | | | | |
| --- | --- | --- | --- | --- | --- | --- | --- | --- |
| Age interval | Category: | k | SMD | 95%-CI | tau^2 | tau | Q | I2 |
| 1-2 weeks | Breastfeeding only | 2 | -0.12 | [-20.25; 20.01] | 2.47 | 1.57 | 115.33 | 99.10% |
|  | Majority breastfeeding | 6 | -0.04 | [ -0.85; 0.77] | 0.46 | 0.68 | 122.14 | 95.90% |
|  | Mixed | 2 | 0.70 | [ -1.03; 2.43] | 0.00 | 0.06 | 2.55 | 60.80% |
|  | Bottle only | 1 | 1.54 | [ 1.08; 1.99] | -- | -- | 0 | -- |
| 5-6 weeks | Breastfeeding only | 3 | -0.27 | [-3.50; 2.96] | 1.08 | 1.04 | 96.11 | 97.90% |
|  | Majority breastfeeding | 4 | 0.26 | [-0.40; 0.92] | 0.11 | 0.33 | 26.44 | 88.70% |
|  | Mixed | 10 | 0.13 | [-0.36; 0.63] | 0.41 | 0.64 | 244.04 | 96.30% |
|  | Bottle only | 2 | -0.11 | [-1.47; 1.24] | 0.00 | 0.00 | 0.75 | 0.00% |


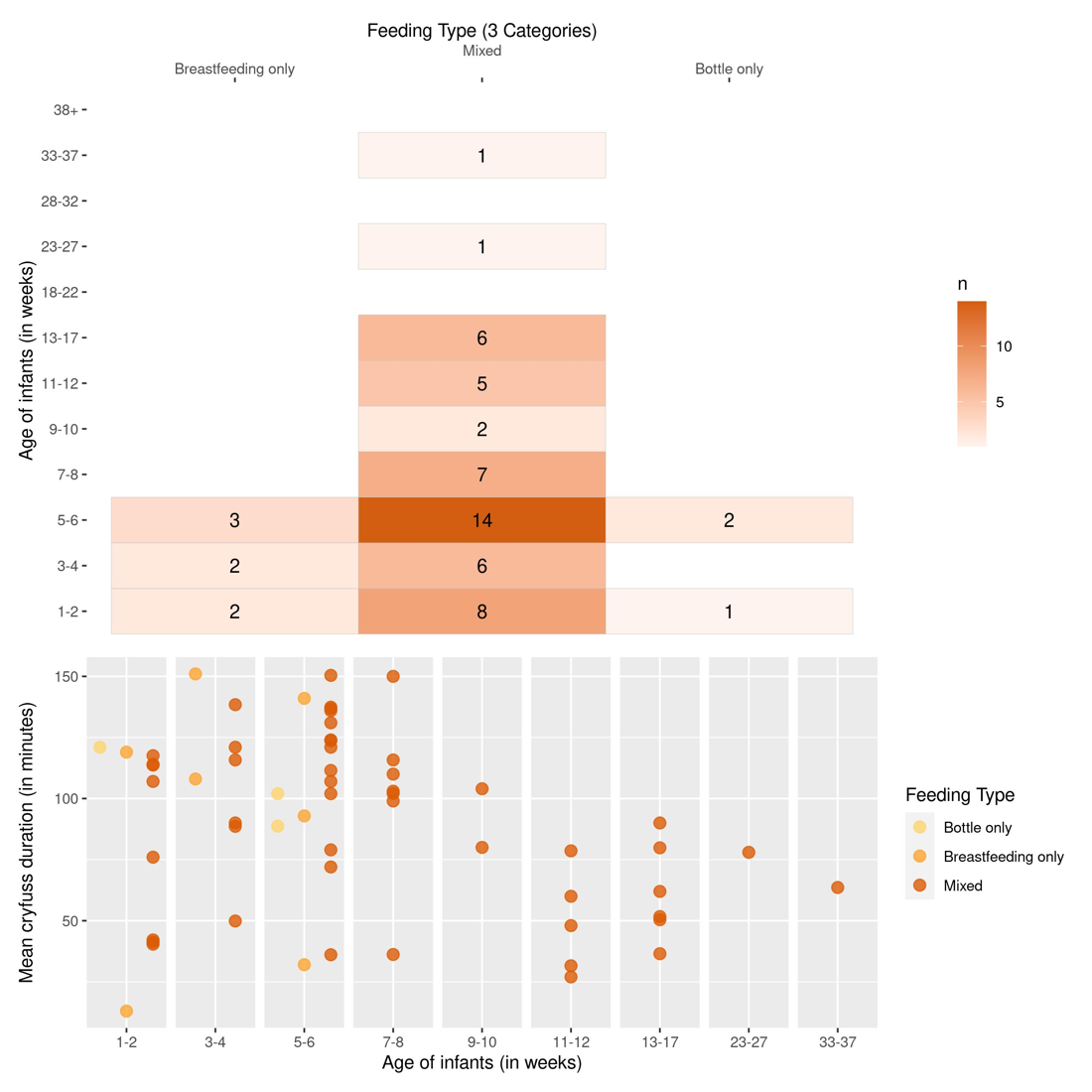


Figure S3a. Description of available samples based on feeding type, according to a 3-levels categorisation scheme (as in Wolke et al., 2017)


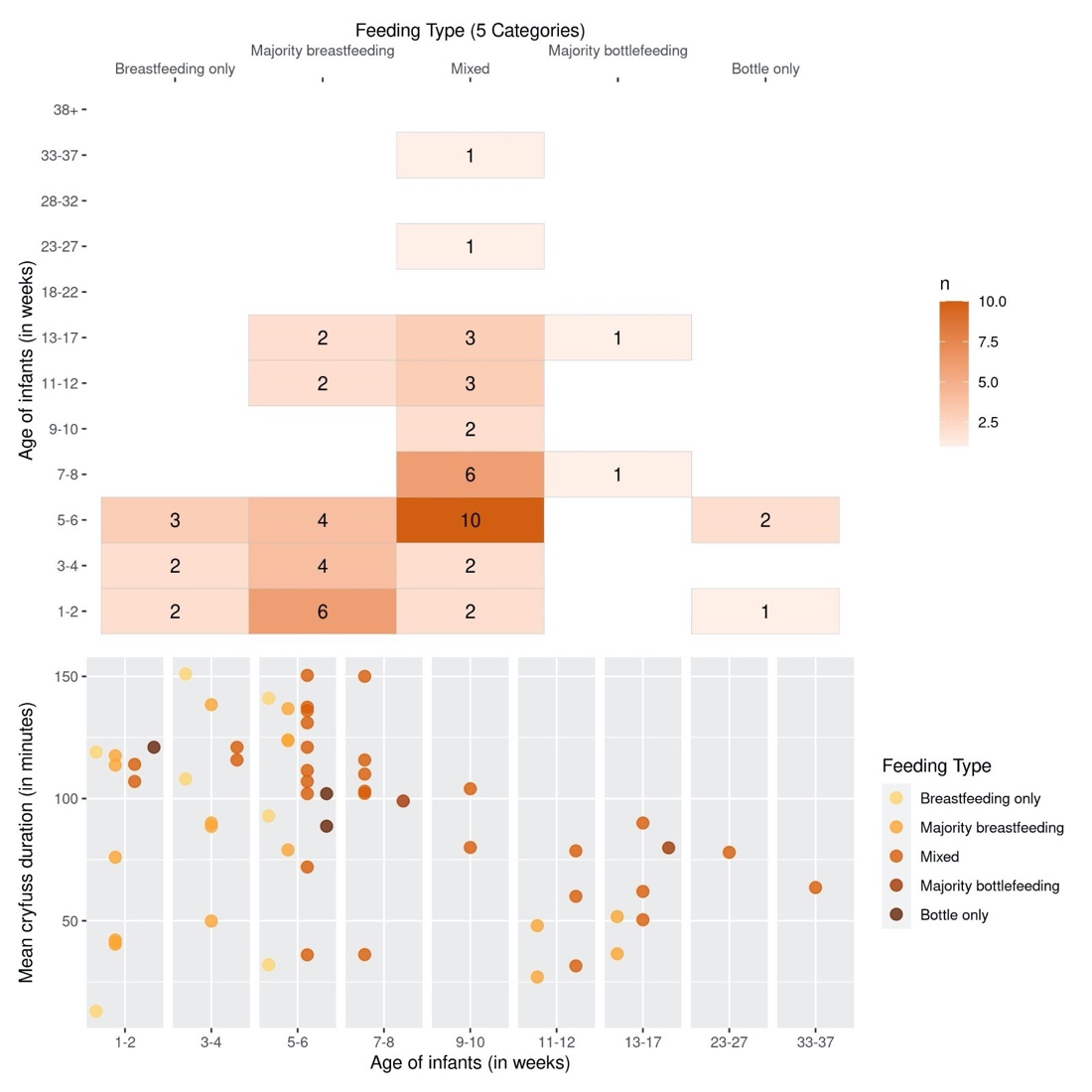


Figure S3b. Summarising the available data for 5 categories of feeding type at the different age intervals (top panel) and mean cry/fuss duration of available samples by feeding type and infant age (bottom panel).

CRY CURVE MODELLING

*Considering the characteristics of the included data*

After excluding the papers that did not report on SD (6 studies), there were 180 samples from all countries and age intervals, all crying measures included. Taking these 180 samples and subtracting 1 SD from the mean, about 19% of samples crossed 0 (see Figure S4), indicating about 15% chance of a “negative” crying duration. Subtracting 2 SDs, approximately 53% crossed 0. Examining the mean and standard deviation indices in this way suggests that the summarised data from the included studies does not follow a normal distribution. For this reason, we decided to use a gamma distribution to model the data. The gamma distribution has the advantage of only taking strictly positive values, therefore, presenting a more skewed profile likely to fit better to the distribution of observed crying durations.


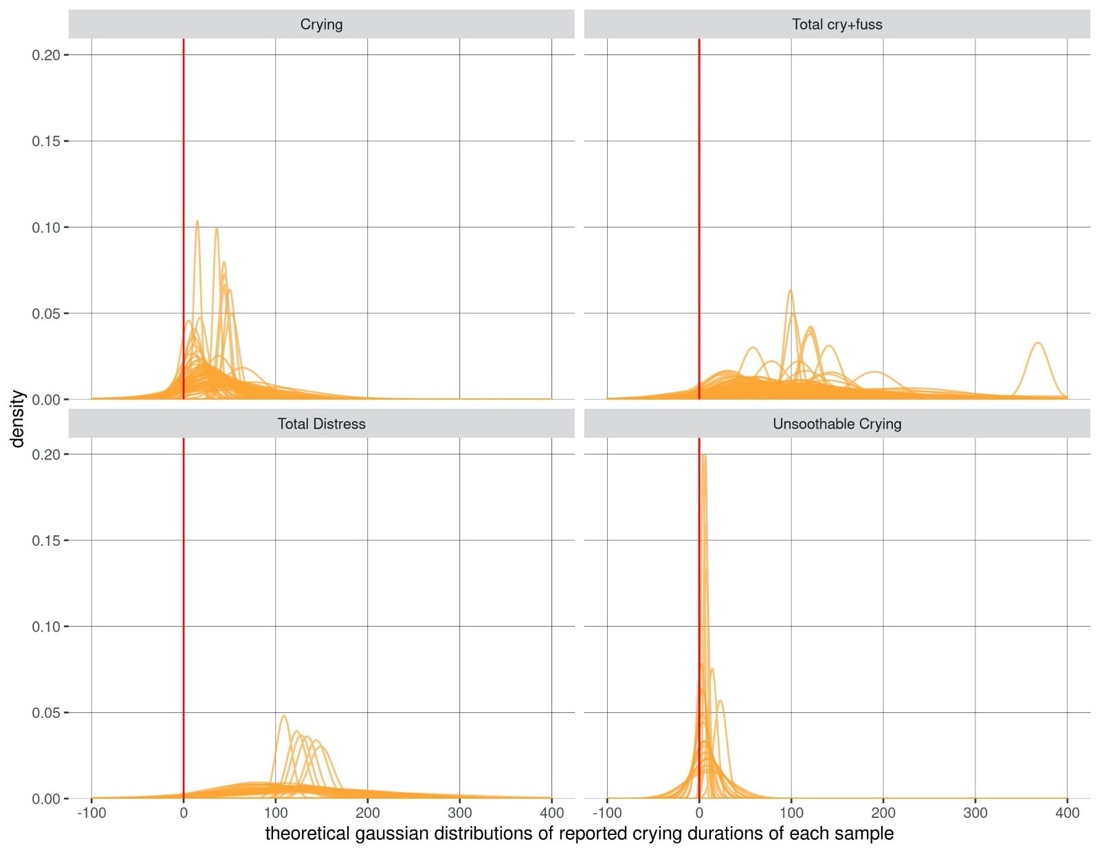


Figure S4. Examining four cry measures and their theoretical gaussian distributions

*Assumptions of the model*

This model relies on two assumptions about the data: (i) mean cry duration for each time point is treated as a separate, independent measure, noting that some studies reported on multiple longitudinal measurements. Only three studies (Lee (2000) = eight measurements, Mohebati (2014) = eight measurements, and Hunziker (1986) = five measurements) included more measured time points than parameters in the double exponent model (i.e., four parameters).

(ii) Infant age was modelled on the scale of weeks. We used mean age of infant if available, and otherwise inferred it.

*Understanding the double exponential function*


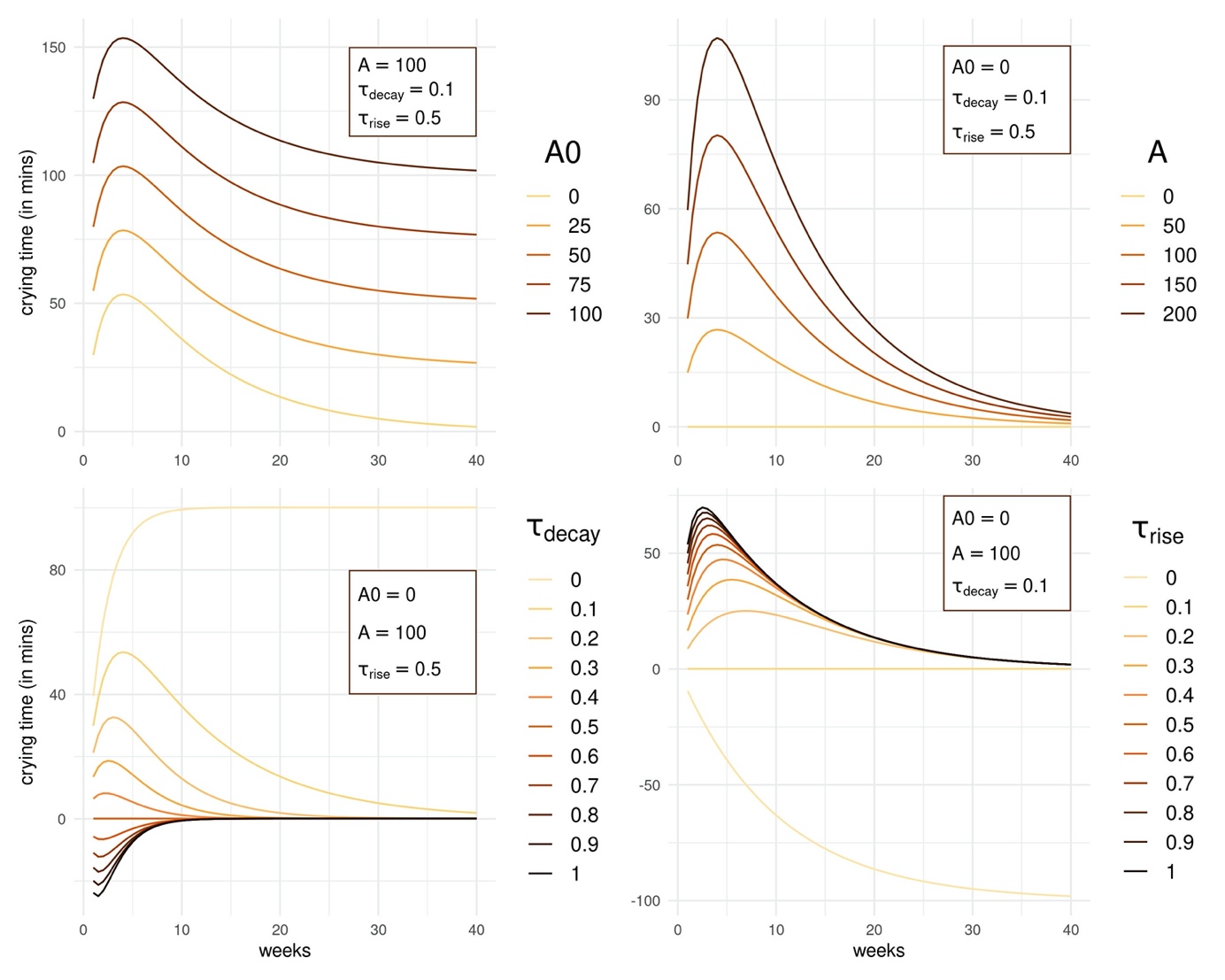


Figure S5. Exploring the parameter space of the double exponential function. On each panel, the value of one parameter is changed while the 3 others remain fixed. The fixed parameter values are indicated in the in-filled boxes while the moving parameter is indicated on the right of the corresponding plot.

*Parameter Recovery: Simulation Study*


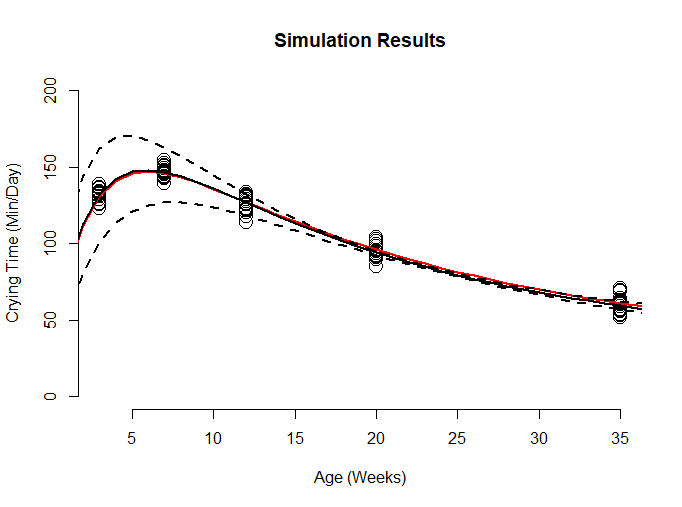


Figure S6. Results of the simulation study, showing good congruence between the inferred (red curve) and the true simulated model (black curve). The chosen parameters for the true model were 𝜏𝑑𝑒𝑐𝑎𝑦 = 0.05, 𝜏𝑟𝑖𝑠𝑒 = 0.4, A = 180, and A = 30. The model was used to generate 20 samples (points in the figure) at each of the timepoints 3 weeks, 7 weeks, 12 weeks, 20 weeks, and 35 weeks, with noise parameter 𝜎 following a Gamma (0.01,0.01). Dotted curves represents the upper and lower 95% credible intervals (defined as the lower and upper 2.5 percentiles of the parameters’ posterior distributions).

*Parameter Recovery: Sensitivity Analysis*

To test whether this congruence was stable across parameter values, we conducted a sensitivity analysis, in which we ran more simulations (n=50) with randomly generated parameter values and compared the true to the inferred (mean posterior) values. The distributions from which the parameters were sampled were:

*𝜏rise~Uniform(.1,1)*

*𝜏decay~Uniform(0,𝜏rise)*

*A~Uniform(50,250)*

*A0~Uniform(0,50)*

*𝜎~Gamma(1,0.1)*


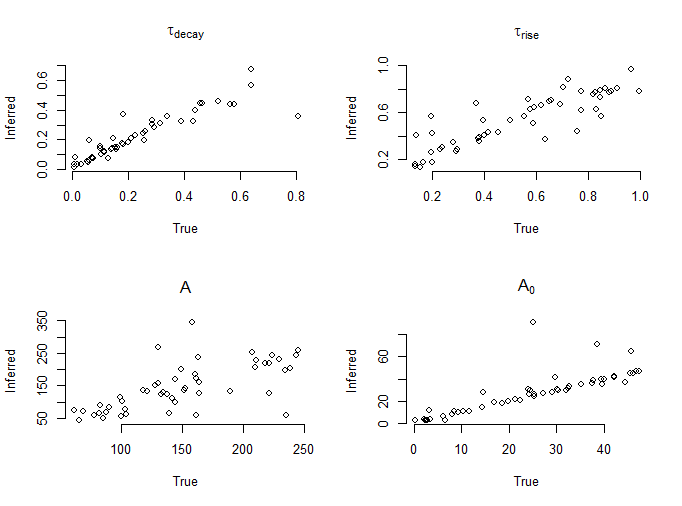


Figure S7. Results of the sensitivity analysis, suggesting reasonable parameter recovery for all parameters, except the scaling parameter *A*. Each dot represents the true value of a parameter against the mean posterior value of the corresponding model.


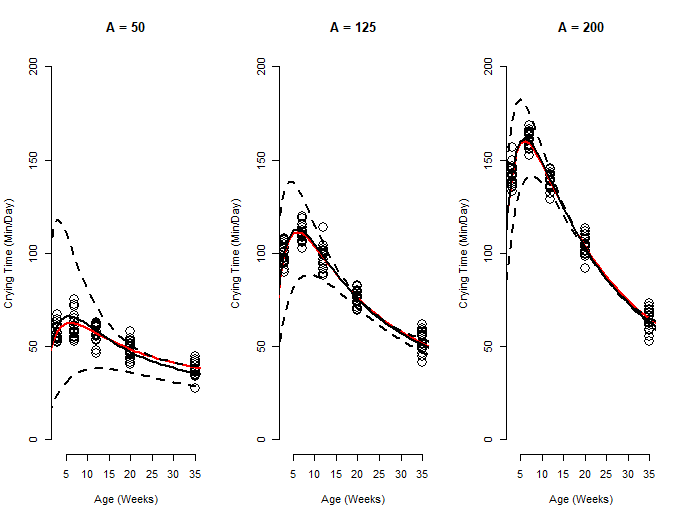


Figure S8. Simulation results for varying values of the scaling parameter A, demonstrating the impact of the identifiability of this parameter. There may be increased risk of error in inference concerning the height of the modelled crying curve, especially for lower values of A.

*Competing model: the change point detection model*

*Parameter Recovery: Sensitivity Analysis*

To test whether this congruence was stable across parameter values, we conducted a sensitivity analysis. We ran 200 simulations with randomly generated parameter values and compared the true to the inferred (mean posterior) values. The distributions from which the parameters were sampled were:

*mu_initial_ ~ Gamma(1, .01)*

*Chi ~ Uniform(0, t_max_)*

*𝜏 ~ Uniform(.1, 1)*

*A0 ~ Uniform(0, mu_initial_)*

*𝜎 ~ Gamma(1,0.1)*

*For each simulation, 20 data points were generated at the following fixed time points: 3, 7, 12, 20, 35 weeks*


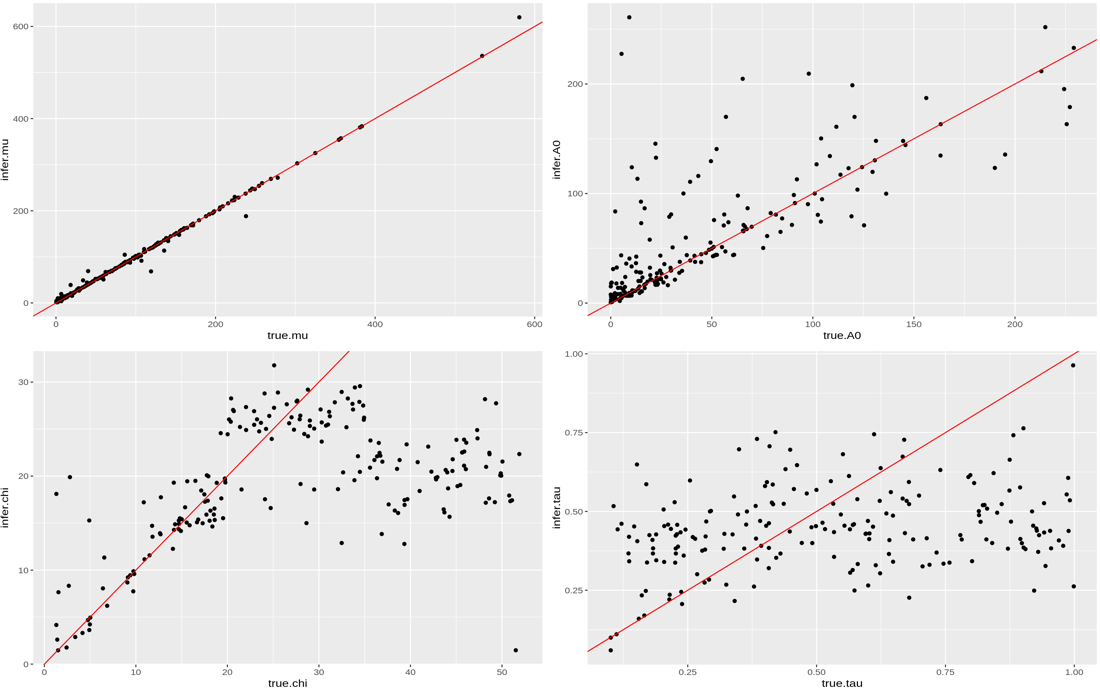


*Figure S9.* Results of the sensitivity analysis for the change point detection model. Each dot represents the true value of a parameter against the mean posterior value of the corresponding model. This suggests poor parameter recovery for *𝜏,* as well as a systematic underestimation of *chi over 20.*

We conducted additional analyses to address the recovery issue of chi, the change point parameter.


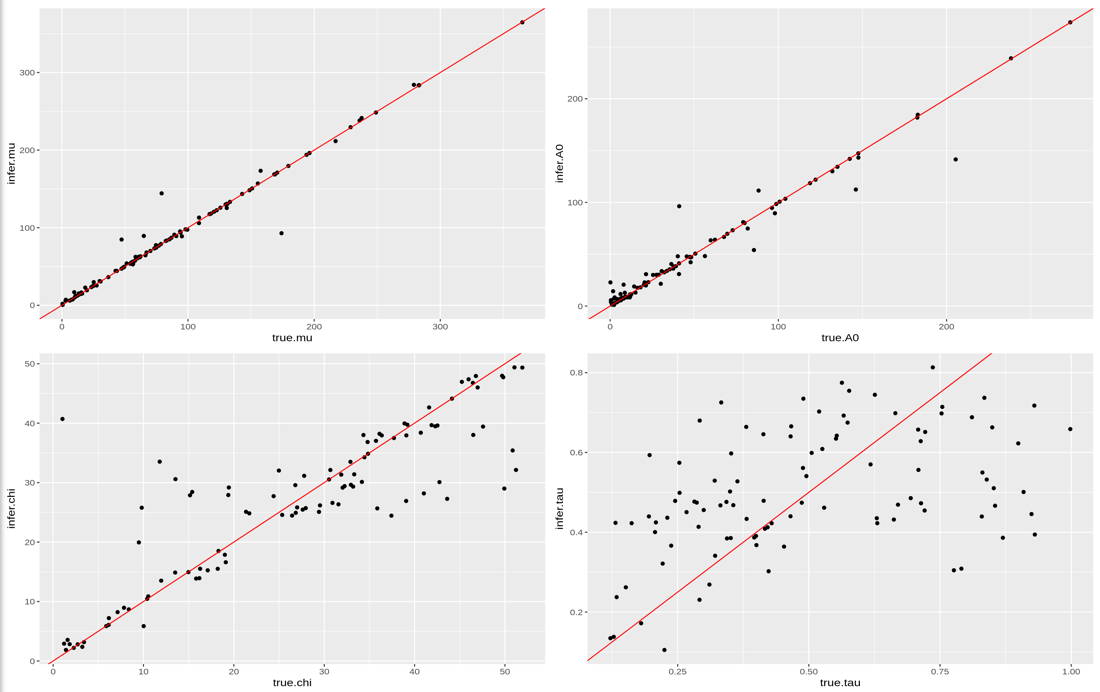


Figure S10. Results of a sensitivity analysis (n = 100 simulations) when adding 20 data points at time points 45 and 52 weeks. This suggests, without surprise, that the reliability of chi estimates over its full range depends on the availability of data along this same time span.


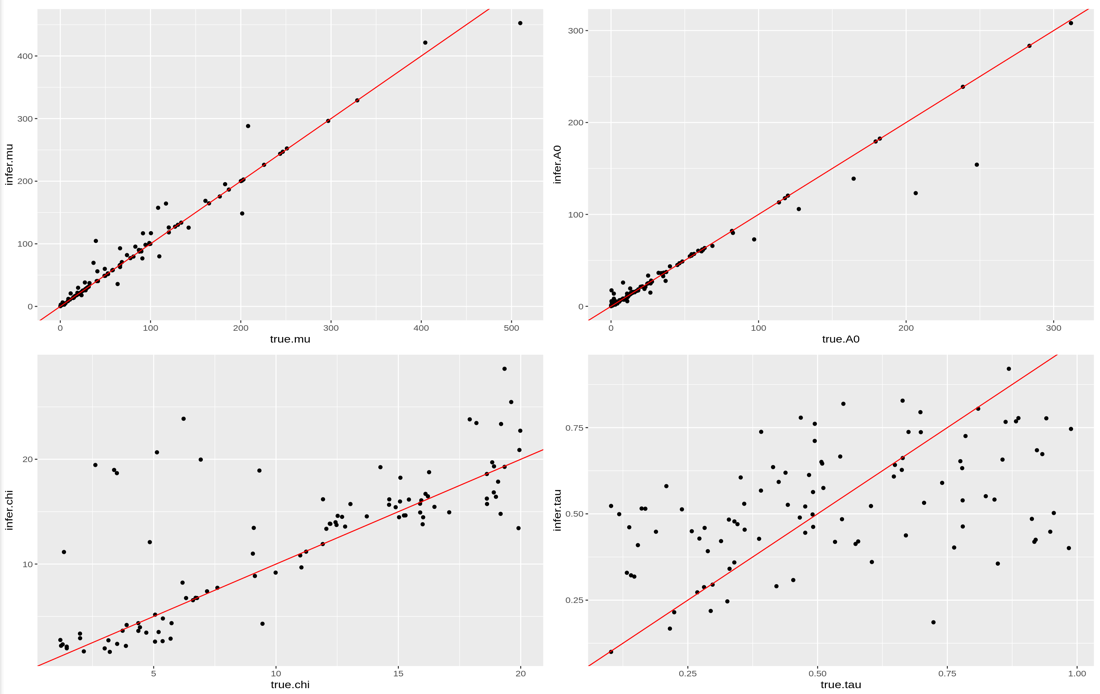


Figure S11. Results of a sensitivity analysis (n = 100 simulations) when chi is drawn from a *Uniform(0, 20),* therefore constraining the true change point to always happen before 20 weeks. This suggests that, despite a lack of data between 20 and 52 weeks, chi can be reliably estimated, as long as its true value is below 20 weeks.

*Model Comparison*


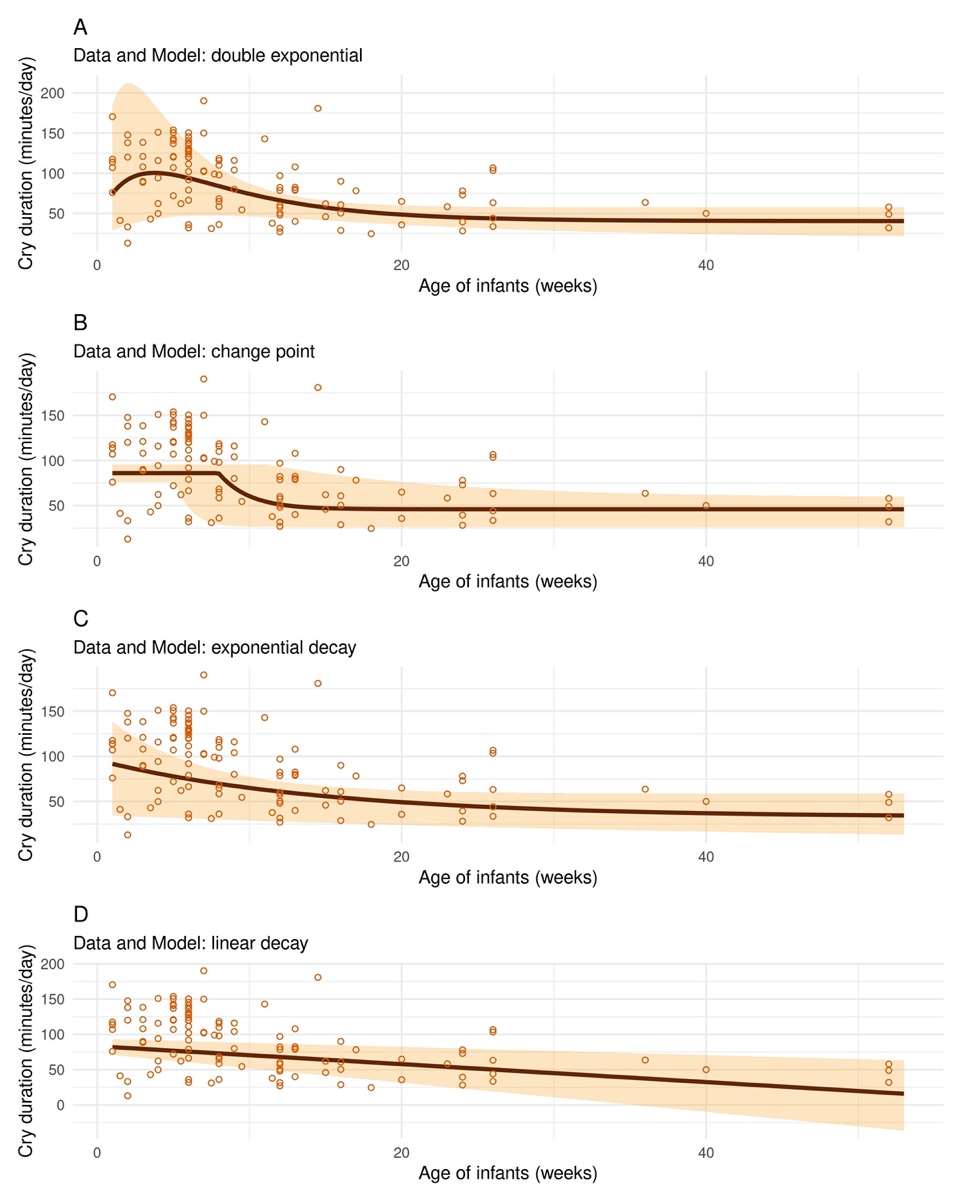


Figure S12. Results of the 4 different models plotted against the data (circles), fitted using the posterior means of the inferred model parameters (solid curve), with uncertainty presented using 95% credible intervals (shaded area).

Table S8. Table describing demographics of the samples used in the meta-analysis. We include this table to comply with the journal's sociocultural policy on data reporting. We are not aware of any theoretical relevance of race or ethnicity for infant crying. We note that reporting of ethnicity/race is sparse in the included studies, and terms that are no longer accepted (e.g., Caucasian) are recorded. In the main manuscript, we provide a description of the skewness of the data towards Western Anglo-Saxon Protestant (WASP) countries. We tabulate the available data, but encourage caution in its interpretation. Detailed information about effect sizes is presented in S Figure 1a and 1b.

| **Author** | **Year** | **Age Group** | **Sample Size** | **Reported Gender (Percentage Girls)** | **Reported Ethnicity** | **Measure** | **Mean minutes** | **SD** | **Effect Size (Hedge's g)** |
| --- | --- | --- | --- | --- | --- | --- | --- | --- | --- |
| Alvarez | 2004 | 3-4 | 118 | - | - | Total cry+fuss | 90.00 | 58.00 | -0.06 |
| Alvarez | 2004 | 5-6 | 111 | - | - | Total cry+fuss | 79.00 | 67.00 | -0.24 |
| Alvarez | 2004 | 11-12 | 110 | - | - | Total cry+fuss | 48.00 | 44.00 | -0.92 |
| Anzman-Frasca | 2013 | 3-4 | 49 | 59 | Infants: 86.4% White | Total cry+fuss | 88.63 | 60.41 | -0.08 |
| Anzman-Frasca | 2013 | 13-17 | 55 | 59 | Infants: 86.4% White | Total cry+fuss | 50.41 | 32.02 | -0.96 |
| Atella | 2003 | 5-6 | 106 | 48 | Mothers: 88% European American. 9% African American. 3% Asian/Asian-American | Total cry+fuss | 124.00 | 62.43 | 0.52 |
| Barr | 1989 | 5-6 | 374 | - | - | Total cry+fuss | 91.88 | 62.24 | -0.03 |
| Bilgin | 2020 | 13-17 | 105 | - | - | Total cry+fuss | 79.84 | 70.52 | -0.22 |
| Bilgin | 2020 | 23-27 | 105 | - | - | Total cry+fuss | 63.30 | 65.38 | -0.5 |
| Blum | 2002 | 5-6 | 60 | - | - | Total cry+fuss | 140.90 | 75.10 | 0.72 |
| Blum | 2002 | 5-6 | 59 | - | - | Total cry+fuss | 127.10 | 69.90 | 0.53 |
| Blum | 2002 | 7-8 | 58 | - | - | Total cry+fuss | 97.90 | 46.90 | 0.09 |
| Bolten | 2012 | 5-6 | 120 | - | - | Total cry+fuss | 140.88 | 70.54 | 0.75 |
| Bonichini | 2008 | 1-2 | 70 | - | - | Total cry+fuss | 147.64 | 90.16 | 0.72 |
| Bonichini | 2008 | 5-6 | 70 | - | - | Total cry+fuss | 150.56 | 100.11 | 0.7 |
| Bonichini | 2008 | 7-8 | 70 | - | - | Total cry+fuss | 118.50 | 78.50 | 0.37 |
| Clifford | 2002 | 5-6 | 431 | - | - | Total cry+fuss | 111.50 | 65.85 | 0.3 |
| Clifford | 2002 | 13-17 | 320 | - | - | Total cry+fuss | 40.08 | 57.21 | -0.96 |
| Darlington | 2006 | 7-8 | 75 | - | - | Total cry+fuss | 110.00 | 69.12 | 0.26 |
| DeLeon | 2007 | 33-37 | 41 | 37 | Infants: 95% non-Hispanic White | Total cry+fuss | 63.60 | 57.60 | -0.53 |
| Fujiwara | 2011 | 5-6 | 1065 | - | - | Total cry+fuss | 153.90 | 67.20 | 0.99 |
| Fujiwara | 2011 | 5-6 | 1857 | - | - | Total cry+fuss | 142.90 | 60.70 | 0.86 |
| Geeraerts | 2020 | 23-27 | 132 | 46 | Infants: 93.29% White or Caucasian. 2.01% Black or African American. 1.34% Hispanic or Latino. 1.34% Asian. 1 infant Native Hawaiian/Pacific Islander. 1 American Indian/Alaskan Native. 1 infant no ethnicity provided. | Total cry+fuss | 73.00 | 39.85 | -0.43 |
| Hiscock | 2014 | 13-17 | 259 | - | - | Total cry+fuss | 90.00 | 54.00 | -0.07 |
| Hiscock | 2014 | 23-27 | 215 | - | - | Total cry+fuss | 78.00 | 54.00 | -0.29 |
| Hunziker | 1986 | 3-4 | 50 | 58 | Infants: 88% White. 6% Black. 6% Chinese/Oriental | Total cry+fuss | 108.00 | 18.00 | 0.35 |
| Hunziker | 1986 | 3-4 | 50 | 58 | Infants: 88% White. 6% Black. 6% Chinese/Oriental | Total cry+fuss | 115.80 | 24.00 | 0.52 |
| Hunziker | 1986 | 5-6 | 50 | 58 | Infants: 88% White. 6% Black. 6% Chinese/Oriental | Total cry+fuss | 137.40 | 36.00 | 0.94 |
| Hunziker | 1986 | 7-8 | 50 | 58 | Infants: 88% White. 6% Black. 6% Chinese/Oriental | Total cry+fuss | 115.80 | 24.00 | 0.52 |
| Hunziker | 1986 | 11-12 | 50 | 58 | Infants: 88% White. 6% Black. 6% Chinese/Oriental | Total cry+fuss | 78.60 | 18.00 | -0.37 |
| Jordan | 2020 | 3-4 | 10 | - | - | Total cry+fuss | 62.30 | 30.20 | -0.68 |
| Jordan | 2020 | 5-6 | 10 | - | - | Total cry+fuss | 66.40 | 31.30 | -0.58 |
| Jordan | 2020 | 7-8 | 10 | - | - | Total cry+fuss | 58.50 | 36.00 | -0.73 |
| Jordan | 2020 | 11-12 | 10 | - | - | Total cry+fuss | 50.40 | 35.60 | -0.9 |
| Keller | 1996 | 13-17 | 13 | 38 | - | Total cry+fuss | 180.80 | 119.39 | 0.91 |
| Killerby | 1992 | 7-8 | 14 | 57 | - | Total cry+fuss | 102.10 | 50.40 | 0.16 |
| Kivijarvi | 2004 | 13-17 | 56 | - | - | Total cry+fuss | 82.49 | 45.80 | -0.22 |
| Kivijarvi | 2004 | 38+ | 56 | - | - | Total cry+fuss | 48.98 | 30.58 | -1 |
| Korja | 2008 | 18-22 | 36 | 47 | - | Total cry+fuss | 64.90 | 40.30 | -0.59 |
| Kramer | 2001 | 3-4 | 91 | - | - | Total cry+fuss | 151.00 | - | 0.91 |
| Kramer | 2001 | 5-6 | 78 | - | - | Total cry+fuss | 131.00 | - | 0.64 |
| Kramer | 2001 | 9-10 | 74 | - | - | Total cry+fuss | 104.00 | - | 0.2 |
| Lam | 2010 | 5-6 | 33 | 42 | Mothers: 52% White. 25% Asian. 12% Filipino. 12% Other | Total cry+fuss | 120.00 | 53.67 | 0.48 |
| Lee | 1994 | 3-4 | 32 | - | - | Total cry+fuss | 94.20 | 79.10 | 0.01 |
| Lee | 1994 | 7-8 | 32 | - | - | Total cry+fuss | 64.90 | 56.40 | -0.51 |
| Lee | 1994 | 11-12 | 32 | - | - | Total cry+fuss | 57.50 | 41.30 | -0.74 |
| Lee | 1994 | 13-17 | 32 | - | - | Total cry+fuss | 60.80 | 63.40 | -0.55 |
| Lee | 1994 | 23-27 | 32 | - | - | Total cry+fuss | 39.40 | 29.60 | -1.22 |
| Lee | 2000 | 1-2 | 143 | - | - | Total cry+fuss | 33.20 | - | -1.5 |
| Lee | 2000 | 3-4 | 143 | - | - | Total cry+fuss | 43.00 | - | -1.23 |
| Lee | 2000 | 5-6 | 143 | - | - | Total cry+fuss | 62.10 | - | -0.72 |
| Lee | 2000 | 7-8 | 143 | - | - | Total cry+fuss | 31.15 | - | -1.56 |
| Lee | 2000 | 9-10 | 143 | - | - | Total cry+fuss | 54.50 | - | -0.91 |
| Lee | 2000 | 11-12 | 143 | - | - | Total cry+fuss | 37.77 | - | -1.37 |
| Lee | 2000 | 13-17 | 143 | - | - | Total cry+fuss | 45.94 | - | -1.15 |
| Lee | 2000 | 18-22 | 143 | - | - | Total cry+fuss | 24.60 | - | -1.74 |
| Lohaus | 2001 | 13-17 | 20 | 45 | - | Total cry+fuss | 78.82 | 59.96 | -0.25 |
| Lucas | 1998 | 1-2 | 92 | 48 | Infants: 62.89% White | Total cry+fuss | 120.07 | 9.70 | 0.67 |
| Lucas | 1998 | 5-6 | 77 | 48 | Infants: 62.89% White | Total cry+fuss | 120.23 | 10.55 | 0.68 |
| Lucassen | 2003 | 7-8 | 45 | - | - | Total cry+fuss | 99.00 | 6.30 | 0.14 |
| Meijer | 2007 | 7-8 | 86 | 49 | - | Total cry+fuss | 103.00 | 76.00 | 0.14 |
| Meijer | 2007 | 38+ | 89 | 49 | - | Total cry+fuss | 32.00 | 24.00 | -1.45 |
| Milgrom | 1995 | 13-17 | 63 | 51 | - | Total cry+fuss | 44.10 | - | -1.19 |
| Milgrom | 1995 | 23-27 | 15 | 51 | - | Total cry+fuss | 33.55 | - | -1.45 |
| Miller | 1993 | 5-6 | 88 | 50 | Mothers: 72% White | Total cry+fuss | 136.80 | 66.00 | 0.71 |
| Miller-Loncar | 2004 | 7-8 | 24 | 38 | Infants: 92% Caucasian. 8% hispanic | Total cry+fuss | 150.00 | 89.98 | 0.75 |
| Mohebati | 2014 | 1-2 | 204 | 53 | - | Total cry+fuss | 41.28 | 36.34 | -1.13 |
| Mohebati | 2014 | 3-4 | 204 | 53 | - | Total cry+fuss | 49.88 | 61.28 | -0.75 |
| Mohebati | 2014 | 5-6 | 204 | 53 | - | Total cry+fuss | 36.07 | 28.43 | -1.32 |
| Mohebati | 2014 | 7-8 | 204 | 53 | - | Total cry+fuss | 36.18 | 37.68 | -1.22 |
| Mohebati | 2014 | 11-12 | 204 | 53 | - | Total cry+fuss | 31.56 | 26.07 | -1.45 |
| Mohebati | 2014 | 13-17 | 204 | 53 | - | Total cry+fuss | 28.80 | 24.49 | -1.53 |
| Mohebati | 2014 | 18-22 | 204 | 53 | - | Total cry+fuss | 35.75 | 54.40 | -1.06 |
| Mohebati | 2014 | 23-27 | 204 | 53 | - | Total cry+fuss | 28.15 | 27.76 | -1.51 |
| Ozturk-Donmez | 2019 | 3-4 | 21 | - | - | Total cry+fuss | 138.42 | 27.09 | 1.02 |
| Ozturk-Donmez | 2019 | 7-8 | 21 | - | - | Total cry+fuss | 190.22 | 24.96 | 2.24 |
| Ozturk-Donmez | 2019 | 11-12 | 21 | - | - | Total cry+fuss | 142.91 | 25.35 | 1.14 |
| Ozturk-Donmez | 2019 | 23-27 | 21 | - | - | Total cry+fuss | 58.33 | 13.24 | -0.87 |
| Popp | 2019 | 23-27 | 13 | 56 | - | Total cry+fuss | 106.67 | 43.76 | 0.26 |
| Shinohara | 2012 | 5-6 | 31 | - | - | Total cry+fuss | 107.00 | 36.00 | 0.29 |
| Shinohara | 2012 | 9-10 | 31 | - | - | Total cry+fuss | 80.00 | 36.00 | -0.29 |
| Shinohara | 2012 | 13-17 | 31 | - | - | Total cry+fuss | 62.00 | 31.00 | -0.7 |
| St.James-Roberts | 2003 | 1-2 | 93 | - | - | Total cry+fuss | 117.54 | 55.98 | 0.43 |
| St.James-Roberts | 1993 | 5-6 | 16 | - | - | Total cry+fuss | 136.00 | 54.00 | 0.76 |
| St.James-Roberts | 1999 | 1-2 | 14 | - | - | Total cry+fuss | 170.42 | 113.88 | 0.84 |
| St.James-Roberts | 1999 | 5-6 | 20 | - | - | Total cry+fuss | 129.00 | 65.11 | 0.58 |
| St.James-Roberts | 1999 | 11-12 | 20 | - | - | Total cry+fuss | 82.25 | 45.36 | -0.22 |
| St.James-Roberts | 2001 | 1-2 | 191 | - | - | Total cry+fuss | 107.00 | 77.00 | 0.2 |
| St.James-Roberts | 2001 | 3-4 | 181 | - | - | Total cry+fuss | 121.00 | 72.00 | 0.43 |
| St.James-Roberts | 2001 | 5-6 | 173 | - | - | Total cry+fuss | 102.00 | 66.00 | 0.14 |
| St.James-Roberts | 2001 | 11-12 | 152 | - | - | Total cry+fuss | 60.00 | 42.00 | -0.69 |
| St.James-Roberts | 2006 | 1-2 | 111 | - | - | Total cry+fuss | 114.00 | 55.46 | 0.37 |
| St.James-Roberts | 2006 | 5-6 | 81 | - | - | Total cry+fuss | 121.00 | 51.61 | 0.51 |
| St.James-Roberts | 2006 | 1-2 | 70 | - | - | Total cry+fuss | 76.00 | 41.15 | -0.36 |
| St.James-Roberts | 2006 | 5-6 | 64 | - | - | Total cry+fuss | 72.00 | 43.08 | -0.44 |
| St.James-Roberts | 1994 | 1-2 | 100 | - | - | Total cry+fuss | 13.00 | - | -2.07 |
| St.James-Roberts | 1994 | 5-6 | 100 | - | - | Total cry+fuss | 32.00 | - | -1.53 |
| St.James-Roberts | 1994 | 11-12 | 99 | - | - | Total cry+fuss | 27.00 | - | -1.67 |
| St.James-Roberts | 1996 | 1-2 | 122 | - | - | Total cry+fuss | 138.00 | 77.00 | 0.66 |
| St.James-Roberts | 1996 | 5-6 | 93 | - | - | Total cry+fuss | 128.00 | 70.00 | 0.55 |
| St.James-Roberts | 1996 | 11-12 | 68 | - | - | Total cry+fuss | 97.00 | 44.00 | 0.07 |
| St.James-Roberts | 1996 | 38+ | 73 | - | - | Total cry+fuss | 50.00 | 32.00 | -0.97 |
| Stifter | 2003 | 5-6 | 128 | - | - | Total cry+fuss | 119.73 | 43.00 | 0.53 |
| Wake | 2006 | 9-10 | 446 | 49 | - | Total cry+fuss | 116.00 | 63.80 | 0.38 |
| Wake | 2006 | 13-17 | 695 | 49 | - | Total cry+fuss | 78.14 | 52.73 | -0.29 |
| Wake | 2006 | 38+ | 426 | 49 | - | Total cry+fuss | 57.89 | 44.14 | -0.72 |
| Walker | 1994 | 1-2 | 21 | - | - | Total cry+fuss | 113.69 | - | 0.37 |
| Walker | 1994 | 7-8 | 21 | - | - | Total cry+fuss | 68.35 | - | -0.55 |
| de Weerth | 2007 | 5-6 | 102 | - | - | Total cry+fuss | 150.40 | 66.30 | 0.93 |
| Wurmser | 2006 | 5-6 | 64 | - | - | Total cry+fuss | 145.60 | 84.40 | 0.73 |
| Wurmser | 2006 | 13-17 | 63 | - | - | Total cry+fuss | 107.90 | 58.20 | 0.25 |
| Wurmser | 2006 | 23-27 | 63 | - | - | Total cry+fuss | 103.40 | 62.00 | 0.17 |
